# A taxonomic revision of the whitefish of lakes Brienz and Thun, Switzerland, with descriptions of four new species (Teleostei, Coregonidae)

**DOI:** 10.3897/zookeys.989.32822

**Published:** 2020-11-09

**Authors:** Oliver M. Selz, Carmela J. Dönz, Pascal Vonlanthen, Ole Seehausen

**Affiliations:** 1 Department of Fish Ecology and Evolution, Centre for Ecology, Evolution & Biogeochemistry, Eawag: Swiss Federal Institute of Aquatic Science and Technology, 6047 Kastanienbaum, Switzerland Swiss Federal Institute of Aquatic Science and Technology Kastanienbaum Switzerland; 2 Aquatic Ecology and Evolution, Institute of Ecology and Evolution, University of Bern, 3012 Bern, Switzerland University of Bern Bern Switzerland; 3 Aquabios GmbH, Les Fermes 57, 1792 Cordast, Switzerland Aquabios GmbH Cordast Switzerland

**Keywords:** adaptive radiation, *
Coregonus
*, ecological speciation, taxonomy, whitefish

## Abstract

The alpha taxonomy of the endemic whitefish of lakes Brienz and Thun, Switzerland, is revised. We evaluate the status of seven known species: *Coregonus steinmanni***sp. nov.**, *Coregonus profundus***sp. nov.** and *Coregonus acrinasus***sp. nov.** are endemic to Lake Thun; *Coregonus Brienzii***sp. nov.** is endemic to Lake Brienz; and *C. alpinus*, *C. albellus*, and *C. fatioi* from lakes Brienz and Thun are redescribed. One of these species, *C. alpinus*, is revised, since the lectotype for this species is incongruent with the species description given by Kottelat (1997) and Kottelat and Freyhof (2007). The name *C. alpinus* is thus retained for the lectotype designated by Kottelat (1997) and a new description of this taxon provided. For the species otherwise described by Kottelat (1997) and Kottelat and Freyhof (2007) as *C. alpinus* the new name *C. profundus* is designated. *Coregonus acrinasus* is genetically partially of allochthonous origin, closely related to the radiation of Lake Constance, and we therefore compare it to all recognized species of Lake Constance, *C. wartmanni*, *C. macrophthalmus*, *C. arenicolus*, and *C. gutturosus*.

## Introduction

The European whitefish (*Coregonus* spp.) provide prime examples of postglacial adaptive radiations, with several lakes in the boreal, subarctic and prealpine climate zones harbouring multiple, often closely related and endemic species. Up to six species can occur in single lakes of the pre-alpine region (Hudson et al. 2016; Dönz et al. 2018), and perhaps even more in the largest lakes of north-east Europe (Bernatchez 2004; Hudson et al. 2007; Kottelat and Freyhof 2007). Many of these radiations diversified after the most recent retreat of the ice shields 10’000 to 15’000 years ago (Bernatchez 2004; Østbye et al. 2005; Hudson et al. 2011). Diversification was by a combination of geographically sympatric and allopatric speciation in boreal and subarctic lakes (Østbye et al. 2005; Præbel et al. 2013) and mainly by geographically sympatric speciation from an ancestral hybrid population in pre-alpine lakes (Hudson et al. 2011). Multiple axes of divergence appear to structure whitefish radiations in Europe that repeatedly and independently evolved ecologically similar sets of species ("ecomorphs" sensu William 1972) which exhibit parallel patterns of divergence in traits related to foraging (i.e., gill raker number, benthic vs. limnetic feeding ecology), physiology (i.e., growth rate, depth partitioning during feeding and breeding) and reproductive ecology (i.e., spawning season and spawning habitat varying along lake depth and along the benthic-pelagic axis) (Fatio 1890; Steinmann 1950; Østbye et al. 2005; Vonlanthen et al. 2009, 2012; Harrod et al. 2010; Lundsgaard-Hansen et al. 2013; Hudson et al. 2016; Dönz et al. 2018; Öhlund et al. 2020). The two common combinations of traits among species in European whitefish radiations are large, fast growing, sparsely gill-rakered, benthivorous fish spawning in shallow water versus small sized, slow growing, densely rakered, zooplanktivorous fish spawning in deep water, but other combinations of some of these traits can also be found in some species (Steinmann 1950; Vonlanthen et al. 2012; Hudson et al. 2016). The number of gill rakers on the first gill arch have shown to be heritable (Rogers and Bernatchez 2007; Roesch et al. 2013). Variation in this trait relates to variation in the relative efficiency of feeding on zooplankton and benthic prey items (Lundsgaard-Hansen et al. 2013; Roesch et al. 2013). Interspecific differences in body shape (Lundsgaard-Hansen et al. 2013) as well as in growth rates resulting in different body size at a given age have both been shown to be heritable too (Rogers and Bernatchez 2007; Lundsgaard-Hansen et al. 2013). A large number of molecular genetic tests of reproductive isolation among sympatric whitefish species in various Swiss lakes have confirmed that sympatric forms are generally genetically clearly differentiated species (Douglas and Brunner 2002; Douglas et al. 2003; Hudson et al. 2011; Vonlanthen et al. 2012; Hudson et al. 2016; Dönz et al. 2018; Feulner and Seehausen 2018; Jacobs et al. 2018; De-Kayne et al. unpublished).

Here, we revise the whitefish species of lakes Brienz and Thun, Switzerland. Whitefish can be found in the large pre-alpine lakes of France, Germany, Austria, and Switzerland, which historically harboured approximately 50 different species native to approximately 30 lakes in three major river drainages: the Rhine, the Danube, and the Rhone (Svärdson 1957; Kottelat and Freyhof 2007; Winkler et al. 2011; Vonlanthen et al. 2012). Part of this species diversity has been lost during increased lake eutrophication in the last century (Vonlanthen et al. 2012). Phylogeographic studies have shown that the European *C. lavaretus* species complex, which diverged from the North American *C. clupeaformis* species complex at least 500,000 years ago, comprises of two divergent mitochondrial lineages (Bernatchez and Dodson 1991, 1994; Douglas et al. 2003; Østbye et al. 2005; Kottelat and Freyhof 2007; Hudson et al. 2011; Winkler et al. 2011). The two lineages overlap in their geographical distribution, whereby the "northern lineage" (mitochondrial N clade) is predominantly found in Scandinavia and the Baltic Sea region while the "central European lineage" (C clade) has a higher frequency of occurrence in the pre-alpine and North Sea region, hence more westerly (Hudson et al. 2011). The entire pre-alpine whitefish radiation is a monophyletic radiation as revealed by genomic AFLP-markers and whole-genome resequencing data when compared to the closest relatives from northern Germany and Scandinavia (Hudson et al. 2011; De-Kayne et al. unpublished). The occurrence of both central and north mitochondrial haplotype lineages within the pre-alpine radiation, today (Hudson et al. 2011) as well as already 5000 years ago (Alonso et al. 2017), and the frequent occurrence of both lineages within species of the radiation suggest that the entire pre-alpine radiation is of hybrid origin (Hudson et al. 2011). The ancient carriers of the two divergent mitochondrial lineages probably correspond to two glacial refugial lineages that came into secondary contact and hybridized before the hybrid population spread across much of western Europe and diversified into the modern species flocks. As the Alpine ice shields retreated, this hybrid population would have colonized the pre-alpine lakes and radiated within each of the larger lakes into several endemic species (Hudson et al. 2011). As a result, both haplotypes are shared among many of the approx. 25 contemporary endemic species that are native to 17 Swiss lakes (Steinmann 1950; Kottelat and Freyhof 2007; Hudson et al. 2011; Vonlanthen et al. 2012). Only one century ago Swiss lakes harboured approximately 35 endemic species of whitefish, but one third of this original diversity has been lost in the middle of the 20^th^ century due to a combination of speciation reversal through hybridization and demographic declines, both driven by loss of habitat for foraging and spawning and possibly of selective regimes, associated with anthropogenic eutrophication of lakes (Vonlanthen et al. 2012; Hudson et al. 2013; Alexander et al. 2017).

In this paper we compile and review morphological, genetic and ecological data for seven species of whitefish from the connected lakes Thun and Brienz, three of which are found in both lakes. Three of the species were previously described as *C. alpinus* Fatio, 1885, *C. albellus* Fatio, 1890, and *C. fatioi* Kottelat, 1997. We describe four new species that are endemic to one of the two lakes. Three of them are endemic to Lake Thun, *C. steinmanni*, *C. profundus* and *C. acrinasus*. One is endemic to Lake Brienz, *C. brienzii*.

One of the previously described species, *C. alpinus* was designated a lectotype by Kottelat (1997) for which the species description (biology and morphology) in Kottelat (1997) and Kottelat and Freyhof (2007) is incongruent and not that of this species. We show this by tracing back Fatio’s description of *C. alpinus* in his compendium on Swiss fauna (1890) and comparing it to the lectotype of *C. alpinus* and to contemporary samples of this species.

One of the newly described species, *C. acrinasus*, shows ancestry contributions from whitefish of Lake Constance, besides its Lake Thun ancestry (Hudson et al. 2011, 2016; Dönz et al. 2018). We therefore do not only compare it to the five other species from Lake Thun but also to the four described species from Lake Constance, namely *C. wartmanni* Bloch, 1784, *C. macrophthalmus* Nüsslin, 1882, *C. arenicolus* Kottelat, 1997, and the extinct *C. gutturosus* Gmelin, 1818.

We studied the type material designated by Kottelat (1997) in his systematic revision of the nomenclature of European freshwater fishes for the three described species from lakes Thun and Brienz, *C. alpinus*, *C. albellus*, and *C. fatioi* and the four described species from Lake Constance, *C. wartmanni*, *C. macrophthalmus*, *C. arenicolus*, and the extinct *C. gutturosus*. Altogether, we compared 240 of our own contemporary samples from lakes Thun and Brienz to these type series.

## Materials and methods

### Study lakes and fish collection

Type material of all currently valid species (based on Kottelat’s (1997) systematic revision of the nomenclature of Swiss whitefish) was inspected in the collections of the Natural History Museum of Geneva and Bern (**MHNG** and **NMBE**, respecitively), Switzerland and in the Steinmann collection of Eawag, Switzerland, that has recently been transferred together with the Seehausen-Eawag collection to the Natural History Museum of Bern (**NMBE**), Switzerland. All contemporary specimens are part of the Seehausen-Eawag collection. In some cases, more than one fish is stored in the same jar and thus we provide next to the NMBE number in brackets the individual labels of each fish with Eawag followed by the individual number.

The different whitefish species in this study derive from different lakes, namely Lake Thun (46°40'N, 7°46'E, surface area 48 km^2^, max depth 217m), Lake Brienz (46°43'N, 7°57'E, surface area 30 km^2^, max depth 261 m), Lake Biel (47°5'N, 7°10'E, surface area 39.3 km^2^, max depth 74 m) and Lake Constance (47°38'N, 9°22'E, surface area and max depth of Upper Lake Constance 473 km^2^ and 251 m and of lower Lake Constance 63 km^2^ and 46 m depth). Lakes Thun and Brienz are among the deepest and most oligotrophic lakes of the northern pre-alpine region. Lake Constance was historically also among the most deep and oligotrophic lakes of the northern pre-alpine region but is today a mesotrophic lake (Vonlanthen and Périat 2013; Vonlanthen et al. 2015; Alexander et al. 2016). Lakes Thun and Brienz are connected through a short stretch of river (the Bödeli Aare) forming a super-lake system, and used to be part of a much larger postglacial lake, Lake Wendel, before high bed rock load from the river Lütschine separated the basin into the current two lake basins several thousand years ago (Steinmann 1950; Ammann et al. 1991; Hantke and Scheidegger 2007). The species flock of the two lakes, except for *C. acrinasus*, forms a monophyletic group based on independent multilocus microsatellite, large AFLP and whole genome datasets (Douglas et al. 2003; Hudson et al. 2011; Hudson et al. 2016; De-Kayne et al. unpublished). Furthermore, within the super-lake system, the populations of each species from the two sister lakes, Thun and Brienz, are more closely related to each other than the different species from the same lake (i.e., species monophyly), suggesting that the origin of the radiation predates separation of Lake Wendel into lakes Brienz and Thun (Hudson et al. 2011; Hudson et al. 2016; Dönz et al. 2018).

Contemporary samples of whole specimens from lakes Thun and Brienz were collected in the course of many projects of the Seehausen research group (Eawag and the University of Bern). Contemporary material (whole specimens and tissue samples) used here was collected in the years 2005, 2011, 2013, 2014 and 2015 in lakes Thun and Brienz, and in 2016 in Lake Biel. Some of the fish were obtained from commercial fisheries catches. Additionally, in lakes Thun and Brienz fishing was done with monofilament bottom- and pelagic gill nets of various mesh size ranging from 5 to 60 mm, and across many depth ranges in the limnetic and benthic habitats of the lakes (details on net fishing protocols can be found in Alexander et al. (2015)). The fish come from three different sampling methods: targeted fishing on known spawning grounds of the different species at the respective spawning season and water depth (Hudson et al. 2011; Hudson 2011; Vonlanthen et al. 2012), targeted fishing each at one spawning site in lakes Thun and Brienz along a depth gradient four times during the whole spawning season of all species (this study), and habitat-stratified fishing of the whole lake during the summer months (Vonlanthen and Périat 2013; Vonlanthen et al. 2015; Dönz et al. 2018). Additionally, individuals of some species were retrieved from local fisherwomen or fishermen. The sampling locations of all contemporary specimens are plotted on a map in the Suppl. material 1: Figure S10.

### Sample processing

Sampling details for the fish collected in the years 2005, 2011, 2013, and 2014 can be found in the corresponding publications (Bittner 2009; Hudson 2011; Vonlanthen et al. 2012; Dönz et al. 2018). For the fish collected in the year 2015 and 2016 the procedure was as follows: upon capture, fish were anaesthetised and subsequently euthanised with appropriate concentrations of MS222 solutions. Muscle tissue and scales below the dorsal fin, as well as a part of the pectoral fin on the right side of the body, were taken for genetic and isotopic analysis and to determine the age of each fish. The left side of each fish was photographed in two ways: once in water in a custom-made photo cuvette and once on a flat surface with the fins spread. Fish were then fixed in 4% formalin solution for at least 1 month and afterwards transferred through a series of ethanol of increasing concentration (30%, 50%) to the final concentration of 70% for storage. Permits for collecting fish in the lakes were issued by the canton of Bern.

In the field the fish were identified to species level as good as possible. Sex, fresh mass (to the nearest 0.1g), ripeness (4 = not ripe; 5 = partially ripe, i.e., slow flow of egg sand sperm when stripped; 6 = ripe, i.e. eggs and sperm flow easily when stripped) and the presence of tubercles on the scales (modified from Kekäläinen et al. 2015: 0 = not present; 1 = small to medium-sized tubercles; 2 = large tubercles) were noted in some but not all of the field campaigns. Fish which were not ripe, and thus where the sex could not be determined externally, were examined internally by opening the abdominal cavity and inspected for the presence of testis or ovaries.

The age of the specimens that were used in this study was determined in the lab by counting the annual growth rings of four scales under a confocal microscope following Lehtonen and Nylund (1995). If the ages differed between the four scales and three out of four scales did not correspond to the same age, further scales were measured to acquire the same age in 75% of the scales.

### Morphological and meristic characters

Morphological measurements and counts on the old type material (N = 31) and on contemporary specimens (N = 340) were taken of 25 body, 19 head, and 4 gill characters with adigital calliper to the nearest 0.1 mm. Twelve meristic characters were counted. The measurements and counts were taken on the left body side of the fish, unless a specific character was missing or deformed, in which case that character was measured or counted on the right side of the fish. The mean of two measurements were taken for each character, whereby the difference between two measurements had to be less than 5%. If agreement was less good, the distance was measured again two times. The average inaccuracy between two measurements taken over all morphological characters was 1.4%. Not all measurements could be taken for several specimens since characters where damaged or absent, and we thus sometimes report incomplete character lists for certain specimens. This results in varying sample sizes for each character. All characters for which we had missing values were not retained in the multivariate ratio analyses (see below). The number of characters used for each analysis is explicitly mentioned in the results section. Most of the morphological and meristic characters follow Hubbs and Lagler (1964). However, we also included some additional characters and refined the measurement of some characters found in Hubbs and Lagler (1964). A brief description of each character can be found in Table 1 for the morphological characters and in Table 2 for the meristic characters. Furthermore, illustrations in Suppl. material 1: Figures S1, S2 depict the measurements of the morphological characters. For all morphological characters the mean and for all meristic characters the mode are reported together with the standard deviation and the range for each species, lake population, and sex. Some times if the sample sizes were too small, no mode could be calculated and thus, we report "na". For the four newly described species, the holotype is included in the range. Both sexes are included for the full range of each character of each species from both lakes Thun and Brienz.

**Table 1.** Morphological characters, their acronyms and a brief description of each character.

Morphological characters	Acronym	Description
**Body**		
Pelvic fin base	PelvFB	Length between insertions of fin
Pelvic fin "spine" length	PelvFS	Length from upper insertion point of fin to tip of spine; the spine is actually an elongated scale structure
Pelvic fin length	PelvF	Length from upper insertion point of fin to tip of longest branched ray
Pectoral fin base	PecFB	Length between insertions of fin
Pectoral fin 1 length	PecF1	Length from upper insertion point of fin to tip of unbranched ray
Pectoral fin 2 length	PecF2	Length from upper insertion point of fin to tip of longest branched ray
Dorsal fin base	DFB	Length between insertions of fin
Length of anterior part of dorsal fin erected	DFAe	Length from anterior insertion point of fin to tip of longest unbranched ray, when fin is fully erected
Length of anterior part of dorsal fin depressed	DFAd	Length from anterior insertion point of fin to tip of longest unbranched ray, when fin is depressed
Length of posterior part of dorsal fin erected	DFPe	Length from posterior insertion point of fin to tip of most posterior branched ray, when fin is erected
Anal fin base	AFB	Length between insertions of fin
Length of anterior part of the anal fin	AFAe	Length from anterior insertion point of fin to tip of longest branched ray, when fin is fully erected
Adipose fin base	AdFB	Length between insertions of fin
Caudal fin length	CF	Length from the middle of hypural plate of the caudal fin (internally this is the expanded bones at the end of the backbone that support the caudal fin, externally where the lateral line scales end) to the tip of the longest unbranched ray either being on the dorsal or ventral part of the caudal fin
Caudal peduncle depth	CD	Vertical distance between dorsal and ventral margins of the caudal peduncle at its narrowest part
Caudal peduncle length	CL	Length from posterior insertion point of anal fin to the middle of the hypural plate of the caudal fin
Length from anterior part of adipose fin to caudal fin base	PAdC	Length from anterior insertion point of adipose fin to the middle of the hypural plate of the caudal fin
Dorsal head length	DHL	Length from tip of snout to most posterior part of the frontal head bone
Prepelvic length	PreP	Length from tip of snout to anterior insertion point of pelvic fin
Preanal length	PreA	Length from tip of snout to anterior insertion point of anal fin
Standard length	SL	Length from tip of snout to the middle of the hypural plate of the caudal fin
Total length	TL	Length from tip of snout to the tip of longest unbranched ray either being on the dorsal or ventral part of the caudal fin
Predorsal length	PreD	Length from tip of snout to anterior insertion point of dorsal fin
Body depth	BD	Vertical distance between dorsal and ventral margins of body from anterior insertion point of dorsal fin to anterior insertion of pelvic fin: not necessarily the greatest body depth
Postdorsal length	PostD	Length from posterior insertion point of dorsal fin to middle of hypural plate of the caudal fin
**Head**		
Eye diameter	ED	Horizontal distance across the midline of the eye from the anterior to the posterior margin of the soft eye tissue
Eye cavity	EC	Horizontal distance across the midline of the eye from the anterior margin of the eye socket to the posterior margin of the eye cavity
Eye height	EH	Vertical distance across the midline of the eye from the dorsal margin of the eye cavity to the ventral margin of the eye cavity
Eye socket	ES	Horizontal distance from the anterior margin of the eye socket to the most anterior point of the the posterior margin of the eye socket
Postorbital length	PostO	Length from posterior margin of the eye to the most posterior point of the operculum
Head length	HL	Length from the tip of snout to most posterior point of the operculum margin
Head depth	HD	The transverse distance between margins at the widest point of the head.
Head width	HW	Distance between the posterior margins of the left and right operculum
Mouth width	MW	The transverse distance between margins of the upper and lower jaw
Upper jaw length	UJ	Length from the tip of the snout to most posterior point of the upper jaw
Lower jaw length	LJ	Length from the most anterior point of the lower jaw to the lower jaw insertion
Lower jaw width	LJW	Length between the anterior left and right side of the lower jaw
Uperr jaw width	UJW	Length between the posterior left and right point of the upper jaw
Length of maxilla	M	Length from the most anterior point of the maxilla to the most posterior point of the maxilla
Snout length	SN	Length from tip of snout to anterior margin of the eye
Snouth depth	SD	Vertical distance from the upper to the lower margin of the rostral plate
Snouth width	SW	Horizontal distance from the left to the right margin of the rostral plate
Interorbital width	IOW	Distance between the anterior margin of the left and right eye cavity
Internarial width	INW	Distance between the right and left nostrils
**Gill**		
Upper arch length	UA	Length of the first hypobranchial (upper arch) from the most anterior point to the joint of the hypo- and ceratobranchial where the middle raker emerges
Lower arch length	LA	Length of the first ceratobranchial (lower arch) from the most anterior point to the joint of the hypo- and ceratobranchial where the middle raker emerges
Middle gill raker length	MGR	Length of the gill raker directly at the joint of the the upper and lower first arch, from the insertion of the gill raker to the tip of the gill raker
Longest gill raker length	LGR	Length of the longest gill raker either on the upper and lower first arch, from the insertion of the gill raker to the tip of the gill raker

**Table 2.** Meristic characters, their acronyms and a brief description of each character.

Mersitic characters	Acronym	Description
Pelvic fin rays	PelvFR	Number of unbranched and branched rays
Pectoral fin rays	PecFR	Number of unbranched and branched rays
Dorsal fin rays	DFR	Number of unbranched and branched rays; the posteriormost dorsal rays are often borne from a single pterygiophore (the bones on which the rays articulate), in such a case the two rays are acounted as 2 rays, rudimentary unbranched rays in front of the fin are counted
Anal fin rays	AFR	Number of unbranched and branched rays; the posteriormost anal rays are often borne from a single pterygiophore (the bones on which the rays articulate), in such a case the two rays are acounted as 2 rays, rudimentary unbranched rays in front of the fin are counted
Lateral line scales	LS	Scales bearing the lateral-line column canal from the head to the end of the hybpural plate of the caudal peduncle
Predorsal scales	PDS	Dorsal scales starting from the posterior end of the head to the anterior insertion of the dorsal fin
Transverse dorsal scales	TDS	"Number of scale rows between anterior insertion of the dorsal fin and the lateral line, not accounting for the lateral line scale and the scale on the dorsal midline (in front of the dorsal fin) "
Transverse anal scales	TAS	"Number of scale rows between anterior insertion of the anal fin and the lateral line, not accounting for the lateral line scale and the scale on the ventral midline (in front of the anal fin) "
Transverse pelvic scales	TPS	"Number of scale rows between anterior insertion of the pelvic fin and the lateral line, not accounting for the lateral line scale and the scale on the ventral midline (in front of the pelvic fin)"
Upper arch gill raker number	UGR	Number of gill rakers on first upper arch; all rakers including rudimentary developed rakers
Lower arch gill raker number	LGR	Number of gill rakers on first lower arch; all rakers including rudimentary developed rakers and the middle raker
Total gill raker number	total GR	Gill raker number of upper and lower arch combined

### Analysis of morphological data

The average sizes of fish from each species differ between lakes enough that for certain species such as for *C. albellus* the average size and the maximum size of adult fish of the population in Lake Brienz do not overlap with the average size and the minimum size of adult fish of the population in Lake Thun (Suppl. material 1: Figures S4–S6). The lakes differ naturally in several abiotic factors (max lake depth, bathymetric slope, average lake temperature, water turbidity; see Alexander et al. 2015) that may be related to the different growth rates of conspecific populations of several species and thus different size-at-age between the lakes (Kirchhofer 1995; Müller et al. 2007). Comparisons with multivariate statistical methods (PCA, LDA see below) are difficult in such cases, when size differences between populations or species are large and there is little to no overlap (Baur et al. 2014). We thus performed separate multivariate ratio analysis (see below for details) on the species from lakes Thun and Brienz. In the Lake Thun dataset the partially allochthonous species *C. acrinasus* was not included in the comparison between the types of the previously described species of *C. alpinus*, *C. albellus*, and *C. fatioi* (type locality Lake Thun) and the contemporary specimens, because the introduction of whitefish from Lake Constance, from which *C. acrinasus* shares genetic ancestry contributions, postdates the collection year of the types. Furthermore, in Lake Brienz we divided the data into two subsets to avoid allometry issues; a subset containing individuals smaller than 163.5 mm SL and one subset containing individuals larger than 163.5 mm SL. This threshold was chosen to retain several small individuals of the three larger whitefish species, *C. alpinus*, *C. brienzii*, and *C. fatioi* for the analysis with all individuals of the small whitefish species *C. albellus*. All four species of Lake Brienz are represented in both subsets albeit unequally distributed.

Multivariate ratio analysis is a method that performs principal component analysis (PCA) and linear discriminant analysis (LDA) on morphological ratios (Baur and Leuenberger 2011; Baur et al. 2014). Analysis of morphological ratios are especially well suited in a taxonomic context (László et al. 2013). A scree plot was used to identify the number of PC-axes that should be retained and plotted. In most cases the first two PC axes were retained, and, in a few cases, the third PC axis was also retained. We thus use the first three axes to visualize shape variation between the species. The eigenvalues of the PC-axes and the loadings of each trait can be found in the Suppl. material 1: Tables S1–S4. We further plotted the scores of each PC-axis against isosize to investigate the contribution of allometry to individual shape PC-axes. Isosize is an isometric size axis defined as the geometric mean of all characters used in the PCA. We report the linear regression coefficient *R* as a metric of the contribution of allometry to each PC-axes (Baur and Leuenberger 2011) (Suppl. material 1: Tables S1–S4). If the relationship between size and shape is strong, then such PC-axes are not informative to distinguish species based on shape itself.

For the development of a species identification key we used LDA analysis on all characters together and on subsets of only head or only body characters for all contemporary specimens from lakes Thun and Brienz separately to calculate the first two ratios of characters that best separate each of the species in each lake. This method also allows to estimate the extent of shape change with size (i.e., the contribution of allometry to these ratios) which is given as δ and describes how good shape discriminates in comparison to size (see Baur and Leuenberger 2011: Page 818, formula 14). In several pairwise species comparisons, we had more variables than individuals which will not allow to calculate the best LDA ratios. In such cases we used a subset of the variables to match the number of individuals. The variables that were retained in this subset were chosen such that possibly informative characters in each pairwise comparison were kept. All the comparisons with a subset of characters are marked in the table and the respective characters that were excluded are listed (Tables 10, 11). Due to large size differences between the species of Lake Brienz the LDA ratios were calculated with three different datasets; once each with individuals larger or smaller than 163.5 mm SL and once with the full-size ranges of all species. Ratios marked in the table with an asterisk (*) have very little or no overlap with other species and were thus used in the identification key and the species diagnoses. All analyses were performed in RStudio v1.0.143 (R Studio Team 2015).

### Genetics

Genetic analysis of ten microsatellite loci were used for the Bayesian clustering algorithm program STRUCTURE (Pritchard et al. 2000) to assign all contemporary specimens of lakes Thun and Brienz to the different whitefish species present in either lake. DNA was extracted from fin tissue using Chelex and Proteinase-K following the manufacturer’s standard protocol. All individuals were genotyped at ten microsatellite loci that were combined into two multiplex sets: CoCl49, CoCl68, CoCl6, C2–157, CoCl61, CoCl45 and BWF-2, CoCl4, CoCl18, CoCl10 (Patton et al. 1997; Turgeon et al. 1999; Rogers et al. 2004). DNA fragments were resolved on an automated DNA sequencer (ABI 3130xl) and genotypes were determined with the software Gene Mapper (ver. 4.0) with the same scoring-panel as in Dönz et al. (2018). Individuals that had missing data at more than two loci were excluded from further analysis.

From the targeted spawning fisheries (each at one spawning site in lakes Thun and Brienz along a depth gradient) a total of 663 individuals from Lake Thun had complete genotypes, ten individuals had one missing locus, and four individuals had two missing loci. A total of 284 individuals from Lake Brienz had complete genotypes, eighteen individuals had one missing locus, and four individuals had two missing loci. These individuals were assigned to the different species using the program STRUCTURE with reference populations of each species deriving from the study by Dönz et al. (2018). A detailed description of the assignment procedure can be found in Dönz et al. (2018). In brief, Dönz et al. (2018) had a dataset comprising 2388 fish from both lakes with the same set of ten microsatellite loci and the same scoring-panel as in our study. To find the most likely number of genetic clusters (K), they conducted a hierarchical cluster analysis (Coulon et al. 2008; Roy et al. 2015) using the individual-based Bayesian clustering algorithm implemented in STRUCTURE (Pritchard et al. 2000). They determined the most likely K for the full dataset of 2388 individuals, then the most likely K within each of the data subsets suggested by the previous analysis, and so forth until all subsets supported a value of K = 1. To determine correspondence of genetic clusters to known species, they assessed how individuals from targeted samplings of known species were distributed among the clusters. They then chose the 50 individuals with highest assignment likelihood to the corresponding clusters at each previous step in the hierarchical analysis and designated them as a reference panel for the six clusters. Afterwards this method can be used to obtain individual genetic assignment proportions to the six clusters inferred in the hierarchical analysis.

We used the reference panel from Dönz et al. (2018) as reference populations and assigned all the individuals from our data set to the six species clusters with the function PopFlag in STRUCTURE. Subsets of 50 individuals out of the 973 genotyped individuals (from the depth gradient data set) were run in separate assignment runs to avoid issues with unequal sample sizes. For each of these analyses, we performed 10 replicates of K = 6 with 200’000 burn-in steps and 200’000 MCMC steps using the admixture and correlated allele frequency model. We used Structure Harvester to generate input files for CLUMPP (ver. 1.1.2, Jakobsson and Rosenberg 2007), which we used to generate consensus percentages of assignment proportions from the 10 structure runs. We first retained all individuals with assignment probabilities higher than 70% to one cluster and chose among these individual’s specimens for the taxonomic work (Dönz et al. 2018). This resulted in a total of 244 out of 677 (36%) individuals for Lake Thun and a total of 147 out of 296 (50%) individuals for Lake Brienz. We aimed at obtaining for each lake a total of ca. 20 specimens of each species for the taxonomic work. In cases where this number was not reached with specimens that had assignment probabilities higher than 70%, we supplemented the data set with individuals with lower assignment probabilities and checked if they cluster in morphospace with the respective specimens with higher assignment probabilities. A few additional specimens were taken from previous sampling campaigns, which were assigned by Dönz et al. (2018). We also took into consideration – next to the genetic species assignment – information regarding catch date and depth (reflecting spawning season and habitat). The percentage of assignment proportions for each contemporary specimen can be found together with all the other data underpinning the analyses reported in this paper in the online Dryad Data Repository (http://doi.org/10.5061/dryad.pd2tq5g).

Newer genomic findings by De-Kayne et al. (unpublished) suggest that whitefish from Lake Brienz, that have previously been assigned based on genetic analysis (see above; Dönz et al. 2018) to *C. steinmanni* and have also been selected based on these assignments for our taxonomic work, actually comprise of an endemic species in Lake Brienz, *C. brienzii*. The assignment probability to *C. steinmanni* of specimens of *C. brienzii*, which is based on the assignment method by Dönz et al. (2018), are for the sake of completeness still reported in the online dataset.

## Results

### Summary

**Figure 1.**
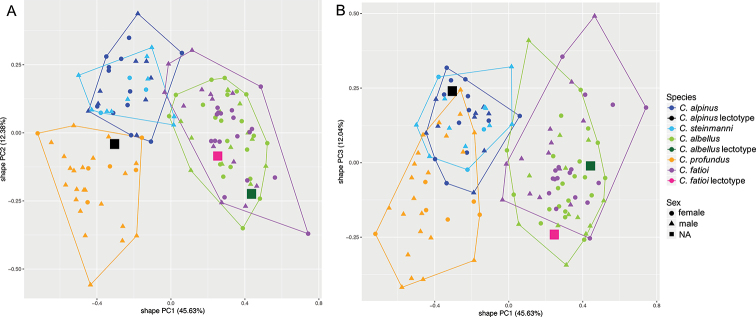
Principal Component Analysis showing that the types of the previously described species *C. alpinus*, *C. albellus* and *C. fatioi* (type locality: Lake Thun) lie within or adjacent to the ranges of the contemporary species of Lake Thun **A, B** shape PCA of the first vs. the second or third PC-axes explain together 70.05% of the variation in shape and are based on a subset (Suppl. material 1: Table S1) of 30 out of a total of 48 measured linear morphological characters (Table 1), since the type material lacked certain characters. Name-bearing types of the formerly described species are highlighted with enlarged symbols in the plots. The proportion of variance explained by each shape PC is given in brackets in the axis legend. PC-loadings and amount of shape variation explained by size are reported in Suppl. material 1: Table S1.

**Figure 2.**
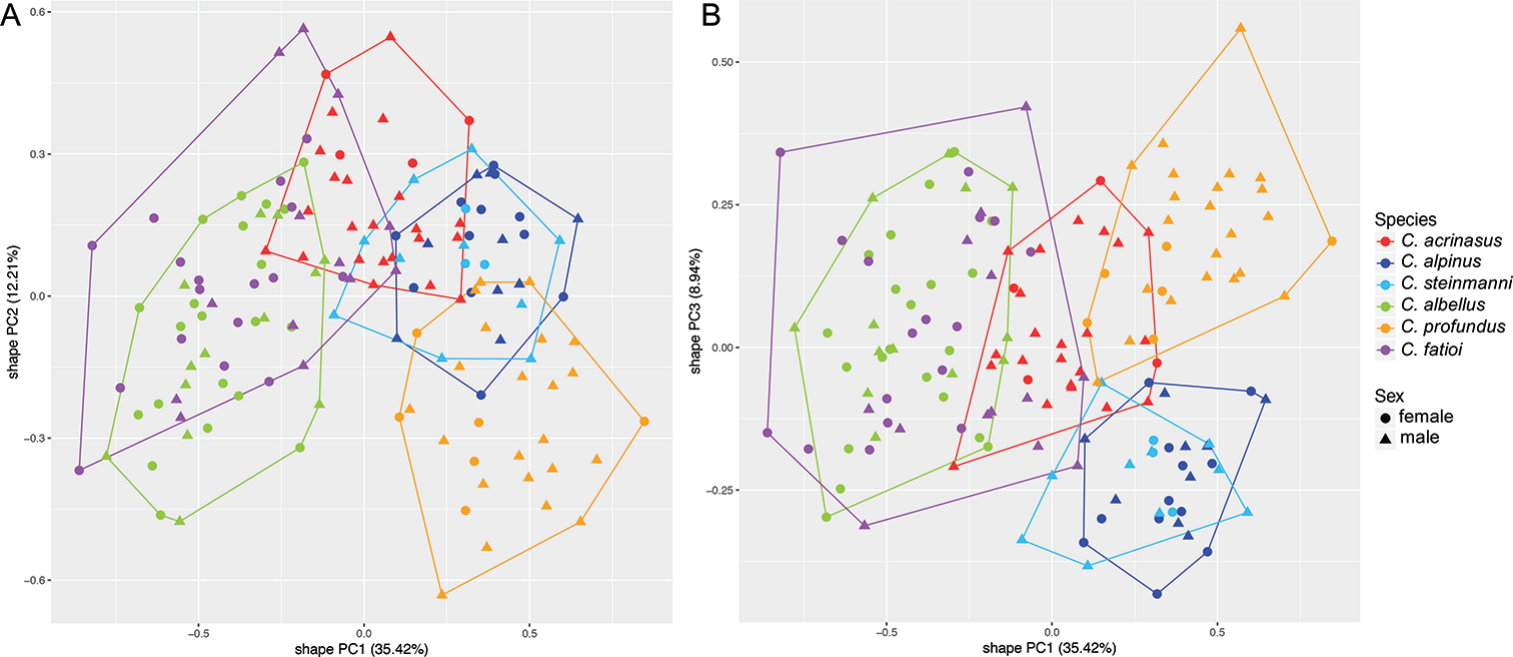
Principal Component Analysis showing the morphospace of the contemporary whitefish species *C. acrinasus*, *C. alpinus*, *C. steinmanni*, *C. albellus*, *C. profundus* and *C. fatioi* from Lake Thun **A, B** shape PCA of the first vs. the second or third PC-axes explain together 56.5% of the variation in shape and are based on all 48 measured linear morphological characters (Table 1). The proportion of variance explained by each shape PC is given in brackets and the PC-loadings and amount of shape variation explained by size in Suppl. material 1: Table S2.

**Figure 3.**
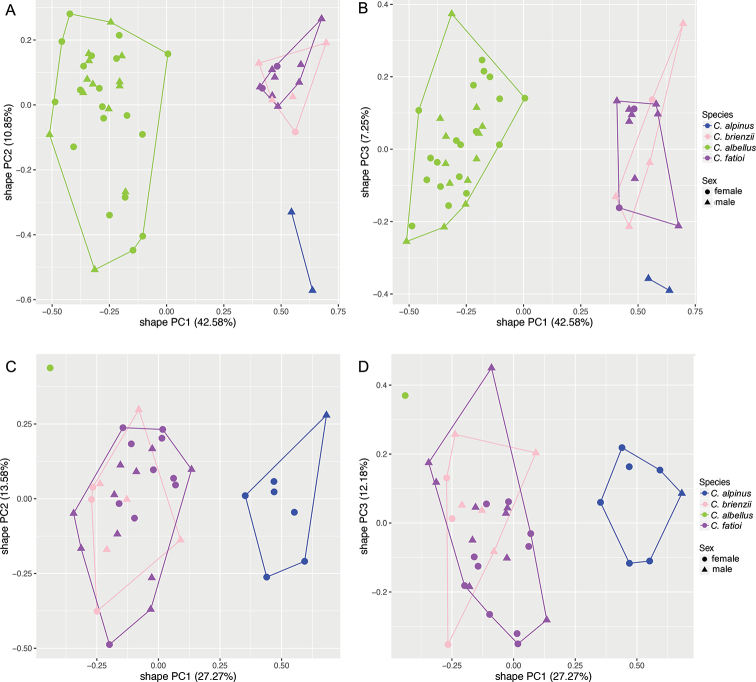
Principal Component Analysis showing the morphospace of the contemporary whitefish species *C. alpinus*, *C. brienzii*, *C. albellus* and *C. fatioi* from Lake Brienz (**A–D**) **A, B** shape PCA of the first vs. the second or third PC-axes explain together 53–60.7% of the variation in shape and are based on all 48 measured linear morphological characters (Table 1), with a dataset containing specimens once smaller (**A, B**) and once larger (**C, D**) than 163.5 mm standard length (SL) to avoid allometry issues. The proportion of variance explained by each shape PC is given in brackets and the PC-loadings and amount of shape variation explained by size in Suppl. material 1: Table S3 (< 163.5 mm) and Suppl. material 1: Table S4 (> 163.5 mm).

The principal component analyses (shapePCA) on the morphological characters show that the type specimens of all previously described species *C. alpinus*, *C. albellus*, and *C. fatioi* group in morphospace within the ranges or adjacent to the ranges of the respective contemporary specimens of these species in Lake Thun (Figure 1a, b; Suppl. material 1: Figure S11a, b). The types of each of the three previously described species further mostly overlap within the ranges of each of the contemporary specimens of the three species from Lake Thun (Tables 3–5). We thus use the contemporary specimens of the previously described species *C. alpinus*, *C. albellus*, and *C. fatioi*, together with the holo- and paratypes of the newly described species *C. steinmanni*, *C. brienzii*, *C. profundus*, and *C. acrinasus* to delineate the species. The shape PCA on the contemporary specimens of Lake Thun shows three main clusters, one containing the species *C. alpinus* and *C. steinmanni*, one containing *C. albellus* and *C. fatioi*, and a third cluster with *C. profundus*, while *C. acrinasus* lies intermediate between these three clusters (Figure 2a, b). The shape PCA on the contemporary specimens of *C. alpinus*, *C. albellus*, *C. fatioi*, and *C. brienzii* of Lake Brienz reveals three clusters, one cluster containing *C. alpinus*, one containing *C. fatioi* and *C. brienzii* , and the third cluster containing *C. albellus* (Figure 3a–d). Some species cluster together in morphospace: In Lake Thun *C. steinmanni* groups with *C. alpinus* and *C. fatioi* with *C. albellus* and in Lake Brienz *C. brienzii* groups with *C. fatioi*. The species can be delineated further by several morphological and meristic characters as well as by morphological ratios extracted from linear discriminant analyses (Tables 3–11). *Coregonus acrinasus* can also be distinguished from all four described Lake Constance whitefish species *C. wartmanni*, *C. macrophthalmus*, *C. arenicolus*, and *C. gutturosus* (Tables 9, 12).

### Species descriptions

#### Coregonus albellus

Taxon classificationAnimaliaTeleosteiCoregonidae

Fatio, 1890

199F0029-D15C-589A-B4D9-4F376F675BB7

Coregonus exiguus albellus : Fatio 1890
Coregonus "Brienzlig": Surbeck 1917; Steinmann 1950, Rufli 1978, 1979; Kirchhofer and Tschumi 1986; Kirchhofer 1995; Bittner et al. 2010
Coregonus "Brienzlig", "Winterbrienzlig": Kirchhofer 1990; Kirchhofer 1995
Coregonus "Small type": Maurer and Guthruf 2005; Müller et al. 2007
Coregonus sp. "winter spawning": Kottelat and Freyhof 2007
Coregonus "Sommerbrienzlig", "BRI2": Douglas et al. 1999; Douglas and Brunner 2002
Coregonus "Sommerbrienzlig", "THU5": Douglas et al. 2003
Coregonus "Sommerbrienzlig", "Winterbrienzlig", "THU4", "THU5": Douglas and Brunner 2002
Coregonus sp. "Brienzlig": Vonlanthen and Périat 2013
Coregonus "Kropfer": Heuscher 1901 (see also synonymy of C. profundus)
Coregonus lavaretus natio *arurensis*, oekot. *nanus*: Steinmann 1950
Coregonus "Zwergalbock": Steinmann 1950

##### Material examined.

***Lectotype*.** MHNG-816.022, Switzerland, Lake Thun (46°40'N, 7°46'E), 165 mm SL, sex unknown.

***Non-types*.** NMBE-1077186–1077202, NMBE-1077221–1077237, Switzerland, Lake Thun (46°40'N, 7°46'E), N = 34, 177–271 mm SL; NMBE-1059754; 1059768; 1059791; 1059801; 1059814, NMBE-1077129–1077131, NMBE-1077318–1077341, Switzerland, Lake Brienz (46°43'N, 7°57'E), N = 32, 101–164 mm SL.

##### Diagnosis.

*Coregonus albellus* is a very small whitefish species in Lake Brienz and a small whitefish species in Lake Thun with weak pigmentation of all fins and body; the colouration on the flanks above the lateral line of specimens from Lake Thun are pale rose to brown and from Lake Brienz pale brown to light green; no or few small pigmented dots on the edge of the scales along the flank for specimens from Lake Thun and specimens from Lake Brienz sometimes have rather large pigmented dots more or less in a row on the upper dorsum; elongate slender body; large eye with a thin and roundish eye socket; tip of snout fleshy and roundish; many and long gill rakers.

##### Differential diagnosis.

No single character was sufficient to distinguish *C. albellus* against all the five other species from Lake Thun and the species is diagnosed by a combination of characters. Based on ratios for the subset of whitefish from Lake Brienz smaller than 163.5 mm, *C. albellus* can be distinguished from the other three species from Lake Brienz by a smaller "postdorsal length / eye height" ratio (PostD/EH: 5.47–6.93 vs. 7.5–8.9). Also, when taking the full-size range (100–290 mm) of all species from Lake Brienz *C. albellus* can be distinguished from the three other species by a smaller "predorsal length / eye height" ratio (PreD/EH: 6.1–7.58 vs. 8.12–10.5) (Table 11).

***Coregonus albellus*-*Coregonus alpinus***

The specimens from lakes Thun and Brienz of *C. albellus* differ from those of *C. alpinus* of both lakes in having a higher number of gill rakers (UGR#: 9–17, mode = 13 vs. 8–11, mode = 10; LGR#: 20–29, mode = 25 vs. 15–23, mode = 19; total GR: 32–44, mode = 38 vs. 25–34, mode = 29), a longer longest gill raker (14.1–21.8% HL, mean = 17.7 vs. 10–15.2% HL, mean = 11.9), a deeper adipose fin (4.5–9.2% SL, mean = 6.5 vs. 3.4–5.5% HL, mean = 4.4), a longer lower jaw (38.4–49.2% HL, mean = 43.6 vs. 33.8–41.4% HL, mean = 38.4) and a thinner eye socket (2– 4.9% HL, mean = 3.4 vs. 3.3–6.3% HL, mean = 5).

In Lake Brienz *C. albellus* further differs from *C. alpinus* by having translucent pelvic and anal fins compared to the moderately to strongly pigmented pelvic and anal fins of *C. alpinus*, a longer pectoral fin (Pectoral fin 1 length: 15.7–22.6% SL, mean = 18 vs. 13.9– 17.9% SL, mean = 16.3; Pectoral fin 2 length: 16.9–23.8% SL, mean = 19.4 vs. 14.4–17.7% SL, mean = 16.9), a longer distance from the anal fin to the hypural plate of the caudal peduncle (17.7– 24.2% SL, mean = 20.7 vs. 15.3–19.5% SL, mean = 17.6), a longer head (16.1– 23.1% SL, mean = 17.9 vs. 14–16.3% SL, mean = 15.4), a larger eye and eye cavity (eye diameter: 26.1–32% HL, mean = 29.3 vs. 21.8–27.2% HL, mean = 24.3; eye height: 26.5– 30.6% HL, mean = 28.7 vs. 22.4– 27.1% HL, mean = 23.9; eye cavity: 30.4–36.8% HL, mean = 33.3 vs. 26.4–31.5% HL, mean = 29), and a longer upper jaw (28.6–34.9% HL, mean = 32.1 vs. 25.4–29.1% HL, mean = 26.8). Finally, *C. albellus* smaller than 163.5mm SL can be distinguished from *C. alpinus* by a smaller "preanal length / lower jaw" ratio (PreA/LJ: 6.33–7.44 vs. 9.24–9.97) and a larger "pectoral fin 2 length / length of the depressed anterior part of the dorsal fin" ratio (PecF2/DFAd: 0.81–1.06 vs. 0.78–0.8). With the full size range of Lake Brienz specimens, *C. albellus* can be distinguished from *C. alpinus* by the smaller "predorsal length / lower jaw" ratio (PreD/LJ: 3.99–4.68 vs. 5.6–6.81), "erected anterior part of the dorsal fin / upper jaw" ratio (DFAe/UJ: 2.14–2.79 vs. 3.25–4.1), "head depth / upper jaw" (HD/UJ: 1.87–2.2 vs. 2.38–2.78) and a larger "lower jaw / interorbital width" ratio (LJ/IOW: 1.53–1.99 vs. 1.33–1.57). (Tables 3, 4, 11).

In Lake Thun *C. albellus* can further be distinguished from *C. alpinus* by having a less deep caudal peduncle (6.4–7.9% SL, mean = 7.1 vs. 7.6–8.9% SL, mean = 8.2) and a longer upper jaw (28.8–34.7% HL, mean = 31.2 vs. 24.3–30.1% HL, mean = 27.7). Based on pigmentation of the fins *C. albellus* can be distinguished from *C. alpinus* from Lake Thun by having translucent to weakly pigmented fins compared to strongly pigmented fins, respectively. In Lake Thun *C. albellus* can further be distinguished from *C. alpinus* by the smaller "caudal peduncle depth / upper jaw length" ratio (CD/UJ: 0.96–1.29 vs. 1.36–1.65) and "caudal peduncle depth / dorsal head length" ratio (CD/DHL: 0.44–0.54 vs. 0.54–0.62) (Tables 3, 4, 10).

***Coregonus albellus*-*Coregonus fatioi***

In Lake Brienz *C. albellus* can be distinguished from *C. fatioi* by having a larger head (16.1–23% SL, mean = 17.9, vs. 14.5–16.8% SL, mean = 15.7), a larger eye and eye cavity (eye diameter: 26.1–32% HL, mean = 29.4 vs. 21.2–27.6% HL, mean = 24.8; eye cavity: 30.4– 36.8% HL, mean = 33.3 vs. 25.3–33% HL, mean = 29; eye height: 26.5–30.6% HL, mean = 28.7 vs. 22.1–26.3% HL, mean = 24.4), a longer maxilla (22.6–26.9% HL, mean = 24.7 vs. 18.7–24.2% HL, mean = 21.7) and longer gill rakers (middle gill raker: 13.7–19.4% HL, mean = 16.5 vs. 10.5–15% HL, mean = 13.2; longest gill raker: 14.9–21.8% HL, mean = 18.2 vs. 12.3–16.4% HL, mean = 14.3). Based on ratios *C. albellus* smaller than 163.5 mm SL can be distinguished from *C. fatioi* by a larger "pectoral fin 2 length / preanal length" ratio (PecF2/PreA: 0.22–0.28 vs. 0.2–0.22), "upper jaw length / eye socket width" ratio (UJ/ES: 6.81–12.42 vs. 4.51–6.15) and "eye socket width / head length" ratio (ES/HL: 0.27–0.31 vs. 0.23–0.27). With the full-size range of Lake Brienz specimens (100–290 mm), *C. albellus* can be distinguished from *C. fatioi* by a smaller "prepelvic length / eye height" ratio (PreP/EH: 6.56–7.98 vs. 8.94–11.43) (Tables 3, 5, 11).

In Lake Thun *C. albellus* can be distinguished from *C. fatioi* by its live colouration above the lateral line on the dorsum ranging from a pale rose to a pale brown compared to a light to dark green colouration in *C. fatioi. C. albellus* can further be differentiated from *C. fatioi* by having no or few small pigmented dots on the edge of the scales or on the boundary of two scales on the flank and dorsum compared to moderate or many dots on the flanks and dorsum in *C. fatioi*.

***Coregonus albellus*-*Coregonus brienzii***

*Coregonus albellus* from Lake Brienz differs from *C. brienzii* by having a longer longest gill raker (14.9–21.8% HL, mean = 18.2 vs. 12.1–16.8% HL, mean = 14.7), a longer maxilla (22.6–26.9% HL, mean = 24.7 vs. 15.4–24% HL, mean = 21), anterior a longer dorsal fin (anterior dorsal fin erected: 17.3–24.7% SL, mean = 19.7 vs. 15.5– 19.8% SL, mean = 17.9; anterior dorsal fin depressed: 18.3–26.6% SL, mean = 20.6 vs. 15.3–20.8% SL, mean = 18.6), a longer head (16.1–23.1% SL, mean = 17.9 vs. 14.6–16.8% SL, mean = 15.6) and a larger eye and eye cavity (eye diameter: 26.1–32% HL, mean = 29.3 vs. 23.1–28.3% HL, mean = 25.3; eye height: 26.5–30.6% HL, mean = 28.7 vs. 22–27.2% HL, mean = 24.4; eye cavity: 30.4–36.8% HL, mean = 33.3 vs. 25.6– 32.9% HL, mean = 29). Based on ratios *C. albellus* smaller than 163.5 mm SL can be distinguished from *C. brienzii* by a larger "maxilla length / eye socket width" ratio (M/ES: 5.35–9.76 vs. 3.31–4.37), "pectoral fin 2 length / predorsal length" ratio (PecF2/PreD: 0.36–0.45 vs. 0.29–0.32), "lower jaw length / eye socket width" ratio (LJ/ES: 9.62–17.28 vs. 6.01–6.49) and a smaller "predorsal length / lower jaw length" ratio (PreD/LJ: 3.99–4.68 vs. 5.05–5.57). With the full size range (100–290 mm) of Lake Brienz specimens, *C. albellus* can be distinguished from *C. brienzii* by a larger "eye height / head length" ratio (EH/HL: 0.27–0.31 vs. 0.22–0.27) and a smaller "predorsal length / eye height" ratio (PreD/EH: 6.1–7.58 vs. 8.12–10.32) (Tables 3, 7, 11).

***Coregonus albellus*-*Coregonus steinmanni***

*Coregonus albellus* from Lake Thun can be distinguished from *C. steinmanni* by having a longer longest gill raker (14.1–20.3% HL, mean = 17.2 vs. 10–14.4% HL, mean = 12.1), a longer maxilla (20.1–26.8% HL, mean = 22.4 vs. 18.1–21.8% HL, mean = 19.7), a less deep caudal peduncle (6.4–7.9% SL, mean = 7.1 vs. 7.5–8.6% SL, mean = 8.0) and a deeper adipose fin (4.5–7.7% SL, mean = 5.8 vs. 3.7–5.4% HL, mean = 4.5). Based on ratios *C. albellus* can be distinguished from *C. steinmanni* by a smaller "caudal peduncle depth / upper jaw length" ratio (CD/UJ: 0.96–1.29 vs. 1.36–1.55) (Tables 3, 6, 10).

***Coregonus albellus*-*Coregonus profundus***

*Coregonus albellus* from Lake Thun differs from *C. profundus* by having more and longer gill rakers (upper arch gill raker number: 9–17, mode = 13 vs. 5–10, mode = 9; lower arch gill raker number: 20–28, mode = 24 vs. 10–18, mode = 14; total number of gill rakers: 32–44, mode = 38 vs. 15–27, mode = 21; middle gill raker length: 11.7– 18.3% HL, mean = 15.6 vs. 7.6–11.7% HL, mean = 9.2; longest gill raker length: 14.1–20.3% HL, mean = 17.2 vs. 7.8–12.4% HL, mean = 10.1). Based on ratios *C. albellus* can be distinguished from *C. profundus* by a larger "caudal peduncle length / eye cavity length" ratio (CL/EC: 1.97–2.87 vs. 1.56–2.09) (Tables 3, 8, 10).

***Coregonus albellus*-*Coregonus acrinasus***

*Coregonus albellus* from Thun can be distinguished from *C. acrinasus* by having a deeper adipose fin (4.5–7.7% SL, mean = 5.8 vs. 3.7–6.2% SL, mean = 4.7), a thinner eye socket (ES: 2–4.9% HL, mean = 3.2 vs. 3.2–6.4% HL, mean = 4.7) and a longer longest gill raker (14.1–20.3% HL, mean = 17.2 vs. 11.4–16.9% HL, mean = 14.5) (Tables 3, 9).

##### Description.

**Figure 4.**
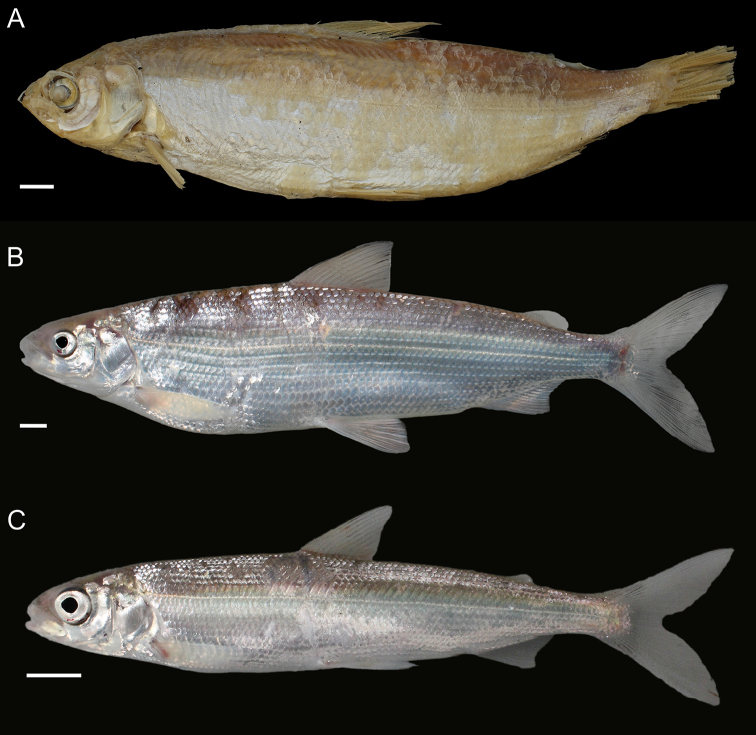
*
Coregonus albellus*, lakes Thun and Brienz, Switzerland **A** lectotype, MHNG-816.022, Lake Thun, 165 mm SL, sex unknown **B** non-type, Eawag-123825, Lake Thun, 221 mm SL, male **C** non- type, NMBE-1077320, Lake Brienz, 115.5 mm SL, male. The white scale (1cm) below each fish acts as a reference for the actual size of the specimen.

General appearance is shown in Figure 4. Morphological and meristic characters of both sexes can be found in Table 3 and Suppl. material 1: Table S6 and first- and second-best ratios for both sexes combined can be found in Tables 10, 11. The description is valid for both sexes and both lakes; differences between the populations of lakes Thun and Brienz are mentioned.

***Shape***: Body elongate, slender. Greatest body depth anterior of the dorsalfin. Ventral profile and dorsal profile similar and slightly arched. Dorsal and ventral profile from tip of snout to interorbital mostly straight and then slightly convex to dorsal and pelvic fin origin respectively. Head long. Snout often 40–50° angle to the body axis anterior of the eye, such that the profile from the tip of the snout to the vertical projection where the anterior part of the eye crosses the dorsal profile is straight and afterwards slightly convex. Mouth (i.e., width of upper and lower jaw) wide, long and often terminal and only rarely slightly sub-terminal. Snout mostly wider than deep, not strongly pronounced, since the tip of the snout is often fleshy and roundish. Large eye, which is more pronounced in specimens from Lake Brienz. Individuals from both lakes have a thin and roundish eye-socket from the middle to the outer margins. Pectoral fin long and moderately tapered. Anterior unbranched ray of the erected dorsal fin range from almost vertically straight to an approx. 70–80° angle to body axis and only bent slightly posteriorly at the end of the ray. Caudal peduncle narrow and elongated with caudal fin forked and sometimes moderately to strongly asymmetrical with either the ventral or dorsal part being longer. Unbranched ray of anal fin straight and rarely bent posteriorly at the end of the ray. Anal fin longest anteriorly and progressively shortening posteriorly with the outer margin of the anal fin slightly concave.

***Meristics ***: Many and long gill rakers.

***Colour***: Pigmentation of fins and body overall weak in live specimens. In specimens from Lake Thun the pectoral fin is translucent, sometimes yellowish with faint pigmentation at the median to distal parts of the fin. Pelvic fin is translucent and only weakly to moderately pigmented. Dorsal, adipose, anal and caudal fins are moderately pigmented. In specimens from Lake Brienz all fins are translucent, with the dorsal, anal and caudal fins sometimes showing some very faint pigmentation. In both lakes fish have a silvery appearance along the flanks and dorsally above the lateral line the silvery appearance changes to a pale rose colouration (e.g., RGB (247, 187, 175)) and then to a pale brown (e.g., RGB (230, 202, 110)). In specimens from Lake Thun the flanks very rarely have few pigmented small dots on the scales. Distribution of dots are bound to the scale patterning (i.e., at the edge of the scales or at the boundary point of two scales. In specimens from Lake Brienz the upper dorsum ranges from pale brown (e.g., RGB (230, 202, 110)) to a light green colouration (e.g., RGB (136, 245, 205)) and sometimes has pigmented dots more or less in a row on the upper dorsum that are rather large ("cheetah look") (Suppl. material 1: Figure S7). Distribution of the dots not restricted to the scale patterning (i.e., at the edge of the scales or at the boundary point of two scales), as can be found for the species of *C. alpinus*, *C. steinmanni*, *C. brienzii* and *C. fatioi*. For a comparison to the main colouration found in the other species see Suppl. material 1: Figure S8. Dorsal part of head of specimens of Lake Brienz is weakly pigmented, whereas that of specimens from Lake Thun is moderately pigmented. Snout around the nostrils is weakly (Lake Brienz) to moderately (Lake Thun) pigmented with a gap of little pigmentation posteriorly of the nostrils up to the height of the middle of the eyes. Operculum and pre-operculum are silvery with one black dot on the lower margin of the pre-operculum. Preserved specimens are pale in colouration with similar pigmentation as described for live specimens. The silvery,translucent,not coloured or unpigmented parts of the body become brown-yellowish (e.g., RGB (239, 210, 40)), whereas the pigmented parts are conserved and the coloured parts (dorsally above the lateral line) become brownish (e.g., RGB (186, 140, 100)).

##### Distribution and notes on biology.

*Coregonus albellus* is found in the lakes Thun (46°40'N, 7°46'E) and Brienz (46°43'N, 7°57'E) that are connected by the short river Bödeli Aare at Interlaken. It is believed to have been endemic to these lakes yet,individual fish have been caught in Lake Biel (47°05'N, 7°10'E) in recent years (since 2005), after it was artificially connected with Lake Thun through the river Aare during the Jura water correction project dating back to 1868–1878. Individuals of *C. albellus* were first identified by local fishermen and fisherwomen, which reported that they had caught small, ripe fish during the summer months (Bittner 2009). The native whitefish species of Lake Biel only spawn in the winter months (Fatio 1885; Steinmann 1950; Rufli 1978). Genetic analysis has shown that these summer-ripe individuals belong to the species *C. albellus* (Bittner 2009). We show for two ripe specimens caught in summer in Lake Biel, genetically assigned based on the assignment method of Dönz et al. (2018) to *C. albellus* with 84% and 94% probability, that they can also be assigned to *C. albellus* based on their morphology (gill raker number, morphological characters) (Suppl. material 1: Figure S9). The species may have established an independent population in Lake Biel, since ripe fish have now been caught for several years in reasonable numbers during the usual spawning period known for this species from Lake Thun (Bittner 2009; Vonlanthen and Périat 2018). *Coregonus albellus* feeds predominantly on zooplankton (stomach content for Lake Brienz: Maurer and Guthruf 2005; Müller et al. 2007; isotopic signature for both lakes: Selz 2008; Hudson 2011; Ingram et al. 2012) and has a slow growth rate (Kirchhofer 1995; Müller et al. 2007; Bittner et al. unpublished). The gill raker number and length of *C. albellus* (many and long gill rakers) also suggests that, based on the functional properties of the number of gill rakers (experimentally tested with specimens of this species and other whitefish species from lakes Thun and Lucerne) (Lundsgaard-Hansen et al. 2013; Roesch et al. 2013), that *C. albellus* feeds predominantly on zooplankton. Habitat-stratified random sampling of lakes Thun (mid-October 2013: Vonlanthen et al. 2015) and Brienz (mid-September 2011: Vonlanthen et al. 2013) show for a snapshot of a few months in summer, that *C. albellus* in Lake Thun occupies the moderately shallow to the deepest benthic waters (approx. 30–217 m; *N* = 29) and the moderately shallow to moderately deep pelagic waters (approx. 10–70 m; *N* = 44) (Dönz et al. 2018). In Lake Brienz *C. albellus* occupies the very shallow (few meters) to the deepest waters (260 m) of the benthic habitat (*N* = 78) and the very shallow to the deeper waters of the pelagic habitat (few meters down to approx. 60 m and exceptionally down to 130 m; *N* = 47) (Dönz et al. 2018). It is to note that the habitat-stratified random sampling data for both lakes only covers a short period of time (one month in late summer) and it is thus not clear how the species are distributed spatially through the rest of the year. Furthermore, the habitat-stratified random sampling in both lakes did not distinguish between ripe and unripe specimens, and thus in the case of *C. albellus* the distribution pattern along the depth in the benthic zone is biased by the spawning aggregation of this species since the sampling period in both lakes coincides with the main spawning season of this species. Most of the whitefish that were phenotypically assigned as *C. albellus* and that were caught in deeper waters during habitat stratified sampling of lakes Brienz and Thun were ripe (PV pers. obs.). In Lake Thun *C. albellus* phenotypically resembles *C. fatioi* and to some extent *C. profundus*. Interestingly, Steinmann (1950) already mentioned for Lake Thun that *C. albellus* (Steinmann, 1950: *Coregonus lavaretus L. nat. arurenis*, *oekot. nanus*; common name: "Zwergalbock" or "Brienzlig") resembles morphologically *C. fatioi* (Steinmann, 1950: *Coregonus lavaretus L. nat. arurenis*, *oekot. pelagicus*; common name: "Schwebalbock" or "Albock"). The average size (total length) at 3 years of age for specimens in this study is 258±13 mm (mean and standard deviation, N = 9) and 152±8 mm (N = 14) for lakes Thun and Brienz, respectively (Suppl. material 1: Figures S4–S6). In Lake Brienz the size of 3-year old specimens of *C. albellus* is considerably smaller than that of the other three whitefish species (*C. alpinus*, *C. brienzii*, *C. fatioi*), whereas in Lake Thun it is similar to that of *C. profundus* and *C. fatioi* (Suppl. material 1: Figure S6) and smaller than that of *C. alpinus*, *C. steinmanni*, and *C. acrinasus*. *Coregonus albellus* has a long spawning season with two peaks. The main spawning peak is in late summer/early autumn from August to October (Locally known as "Sommer-Brienzlig") and the second peak is in early to late winter from December to March (locally known as "Winter-Brienzlig") (Suppl. material 1: Figure S3; Bittner 2009; Dönz et al. 2018). Spawning depth varies with spawning season and can range from approx. 30 m to max. lake depth at 217 m in Lake Thun and approx. 50 m to max. lake depth at 261 m in Lake Brienz (Suppl. material 1: Figure S3; Bittner 2009; Dönz et al. 2018). The spawning season and depth of *C. albellus* partially overlaps with that of *C. steinmanni*, *C. fatioi*,and *C. profundus* in Lake Thun and with that of *C. brienzii* and *C. fatioi* in Lake Brienz.

##### Common names.

Brienzlig, Brienzling; often the time of the year the fish is caught on the spawning grounds is added to the name and shows that this species has a very wide spawning season: Sommer-Brienzlig (for summer) or Winter-Brienzlig (for winter). This species was historically known by local fishermen and fisherwomen as white whitefish (German: "Weissfelchen", but also Albele and Albuli). The common name for this species today is Brienzling which has an ending that is known as a diminutive suffix.

#### Coregonus alpinus

Taxon classificationAnimaliaTeleosteiCoregonidae

Fatio, 1885

C16FB704-958A-59E7-B9F5-A267D349A919

Coregonus "Albock": Rufli 1978, 1979; Kirchhofer and Tschumi 1986; Kirchhofer 1995 (see also synonymy of C. steinmanni and C. acrinasus)
Coregonus balleus : Fatio 1885
Coregonus "Balchen", "THU2": Douglas et al. 1999, 2003; Douglas and Brunner 2002 (see also synonymy of C. steinmanni)
Coregonus "Balchen": Heuscher 1901; Surbeck 1917 (see also synonymy of C. steinmanni)
Coregonus "Felchen": Kirchhofer 1990; Kirchhofer 1995 (see also synonymy of C. fatioi and C. brienzii)
Coregonus "Large type": Maurer and Guthruf 2005; Müller et al. 2007 (see also synonymy of C. fatioi and C. brienzii)
Coregonus lavaretus natio *arurensis*, oekot. *litoralis*: Steinmann 1950
Coregonus lavaretus natio *arurensis*, oekot. *primigenius*: Steinmann 1950 (see also synonymy of C. fatio and C. steinmanni)
Coregonus schinzii alpinus : Fatio 1885
Coregonus schinzii helveticus : Fatio 1890
Coregonus schinzii helveticus var. Thunensis: Fatio 1890
Coregonus sp. "Balchen": Hudson et al. 2011, 2013, 2016; Ingram et al. 2012; Vonlanthen et al. 2012, 2015; Lundsgaard-Hansen et al. 2013; Roesch et al. 2013; Vonlanthen and Périat 2013 (see also synonymy of C. steinmanni and C. brienzii)
Coregonus sp. "Balchen 1": Dönz et al. 2018
Coregonus "Albock", "Uferalbock": Steinmann 1950 (see also synonymy of C. steinmanni and C. fatioi)

##### Material examined.

***Lectotype*.** MHNG-717.045, Switzerland, Lake Thun (46°40'N, 7°46'E), 283 mm SL, sex unknown.

***Non-types*.** NMBE-1077241–1077261, Switzerland, Lake Thun (46°40'N,7°46'E), N = 21, 210–364 mm SL; NMBE-1059817; 1059821; 1077134, NMBE-1077110– 1077115, Switzerland, Lake Brienz (46°43'N, 7°57'E), N = 9, 147–290 mm SL.

##### Diagnosis.

*Coregonus alpinus* is a large whitefish with strong pigmentation of all fins and the body; greenish blue colour on the flanks above the lateral line; moderate to many pigmented small dots on the scales along the flank and the dorsum; deep bodied; truncated blunt snout; short head; sub-terminal mouth; small eye with a thick and triangular-shaped eye socket; short and stout caudal peduncle; few and short gill rakers.

##### Differential diagnosis.

Differential diagnosis against *C. albellus* is given under that species account. The total number of gill rakers of 25 to 34 with mode-values of 28, 29, and 30 distinguishes *C. alpinus* from all other six whitefish species of lakes Thun and Brienz, by either having more gill rakers than the species *C. profundus* (total GR: 15–27, mode = 21) or fewer gill rakers than *C. fatioi* (total GR: 32–43, mode = 38), *C. albellus* (32–44, mode = 38), *C. steinmanni* (30–35, mode = 31), *C. brienzii* (32–39, mode = 37) and *C. acrinasus* (30–40, mode = 36) (Suppl. material 1: Table S6). The contemporary gill raker range is congruent with the historical gill raker range (23–27) given in Fatio (1890).

For specimens in Lake Brienz smaller than 163.5 mm SL *C. alpinus* can be distinguished from the other three whitefish species by a larger "length of the depressed anterior part of the dorsal fin / lower jaw length" ratio (DFAd/LJ: 2.57–2.58 vs. 1.6–2.1). For fish larger than 163.5 mm SL, *C. alpinus* can be distinguished from *C. brienzii* and *C. fatioi* by a larger "length of the erected anterior part of the dorsal fin / upper jaw length" ratio (DFAe/UJ: 3.28–4.1 vs. 2.58–3.19). With the full-size range (100–290 m) of Lake Brienz specimens, *C. alpinus* can be distinguished from the other three whitefish species by a larger "length of the erected anterior part of the dorsal fin / upper jaw length" ratio (DFAe/UJ: 3.25–4.1 vs. 2.14–3.19) (Table 11).

***Coregonus alpinus* - *Coregonus fatioi***

The specimens from lakes Thun and Brienz of *C. alpinus* can be distinguished from those of *C. fatioi* by having a shorter under jaw (24.3–30.1% HL, mean = 27.4 vs. 27.6–34.1% HL, mean = 30), and a shorter longest gill raker (10–15.2% HL, mean = 11.9 vs. 12.3–22.6, mean = 15.6).

In Lake Brienz *C. alpinus* can be distinguished from *C. fatioi* by having a shorter caudal peduncle (11.3–13.9% SL, mean = 12.5 vs. 13.1–16.1% SL, mean = 14.2) and a shorter and narrower lower jaw (lower jaw length: 33.8–39.4% HL, mean = 38.2 vs. 37.6–48.4% HL, mean = 42.6; lower jaw width: 7.3–10.6% HL, mean = 8.8vs. 8.6– 13.3% HL, mean = 11.6). For fish from Lake Brienz larger than 163.5 mm SL, *C. alpinus* can be distinguished based on ratios from *C. fatioi* by having a larger "length of the erected anterior part of the anal fin / upper jaw length" ratio (AFAe/UJ: 1.96–2.5 vs. 1.66–1.96) and a larger "head length / upper jaw length" ratio (HL/UJ: 3.55–3.93 vs. 3.13–3.55). With the full size range (100–290 mm) of Lake Brienz specimens, *C. alpinus* can be distinguished from *C. fatioi* by having a larger "length of the erected anterior part of the dorsal fin / upper jaw length" ratio (DFAe/UJ: 3.25–4.1 vs. 2.14–3.19) (Table 11).

In Lake Thun *C. alpinus* can be further distinguished from *C. fatioi* by having a shorter postdorsal length (38.3–43.9% SL, mean = 42.7 vs. 41.6–50.7% SL, mean = 44.9) and a thicker eye socket (3.4–6.3% HL, mean = 5.1 vs. 1.7–5.9% HL, mean = 3.6). Based on ratios *C. alpinus* can be distinguished from *C. fatioi* by having a larger "caudal peduncle depth / postdorsal length" ratio (CD/PostD: 0.17–0.21 vs. 0.14–0.17) (Tables 4, 5, 10).

***Coregonus alpinus*-*Coregonus brienzii***

*C. alpinus* from Lake Brienz can be differentiated from *C. brienzii* by having a shorter caudal peduncle (11.3–13.9% SL, mean = 12.5 vs. 12.2–15.8% SL, mean = 13.8), a shorter upper and lower jaw (upper jaw: 25.4–29.1% HL, mean = 26.8 vs. 27.1–32% HL, mean = 29.5; lower jaw: 33.8–39.4% HL, mean = 38.2 vs. 40.5–45.7% HL, mean = 42.2), a narrower snout (14.6–17.6% HL, mean = 15.7 vs. 15.7–20.2% HL, mean = 17.8), a narrower lower jaw (7.3–10.6% HL, mean = 8.8 vs. 10.1–14.1% HL, mean = 11.5) and shorter gill rakers (middle gill raker length: 8.3–11.2% HL, mean = 9.8 vs. 10.9–15.1% HL, mean = 13.5; longest gill raker length: 10–12.3% HL, mean = 10.8 vs. 12.1–16.8% HL, mean = 14.7). For fish larger than 163.5 mm SL, *C. alpinus* from Lake Brienz can be distinguished based on ratios from *C. brienzii* by having a larger "caudal peduncle depth / snout width" ratio (CD/SW: 2.25–2.64 vs. 1.82–2.04), "length of the erected anterior part of the dorsal fin / length from the adipose fin to the caudal fin base" ratio (DFAe/PAdC: 1.11–1.32 vs. 0.96–1.16) and by having a smaller "lower jaw width / upper jaw width" ratio (LJW/UJW: 0.33–0.44 vs. 0.45–0.55). With the full size range (100– 290 mm) of Lake Brienz specimens, *C. alpinus* can be distinguished from *C. brienzii* by having a larger "length of the depressed anterior part of the dorsal fin / lower jaw width" ratio (DFAd/LJW: 9.84–14.82 vs. 6.05–8.91), "dorsal head length / lower jaw length" ratio (DHL/LJ: 1.84–2.22 vs. 1.63–1.82), "head depth / lower jaw width" ratio (HD/LJW: 6.72–9.39 vs. 5.23–6.66), "head length / lower jaw length" ratio (HL/LJ: 2.54–2.96 vs. 2.19–2.47) and a smaller "length of the pectoral fin 2 / length of the depressed anterior part of the dorsal fin" ratio (PecF2/DFAd: 0.74–0.85 vs. 0.85–1.03) (Tables 4, 7, 11).

***Coregonus alpinus* - *Coregonus profundus***

*Coregonus alpinus* from Thun differs from *C. profundus* by having shorter pectoral fins (pectoral fin 1 length: 13.6–18.7% SL, mean = 16.2 vs. 16.6–21% SL, mean = 18.4; pectoral fin 2 length: 15.3–19.7% HL, mean = 17 vs. 17.7–23.2% SL, mean = 20.2), a deeper caudal peduncle (7.6–8.9% SL, mean = 8.2 vs. 6.5–7.9% SL, mean = 7.3), a shorter head (12.6–15.6% SL, mean = 14.2 vs. 15.5– 18.4% SL, mean = 16.4) and longer gill rakers (middle gill raker length: 9.3–15.2% HL, mean = 11.3 vs. 7.6–11.7% HL, mean = 9.2; longest gill raker length: 10.6–15.2% HL, mean = 12.3 vs. 7.8–12.4% HL, mean = 10.1). Based on ratios *C. alpinus* can be distinguished from *C. profundus* by having a larger "caudal peduncle depth / dorsal head length" ratio (CD/DHL: 0.54–0.62 vs. 0.4–0.49) (Tables 4, 8, 10).

***Coregonus alpinus* - *Coregonus acrinasus***

*Coregonus alpinus* can further be differentiated from *C. acrinasus* by having a shorter lower jaw (36.6–41.4% HL, mean = 38.6 vs. 38.6–47% HL, mean = 40.9). Based on ratios *C. alpinus* can be distinguished from *C. acrinasus* by having a larger "caudal fin length / maxilla length" ratio (CF/M: 5.55–6.55 vs. 4.4–5.57) (Tables 4, 9, 10).

##### Description.

**Figure 5.**
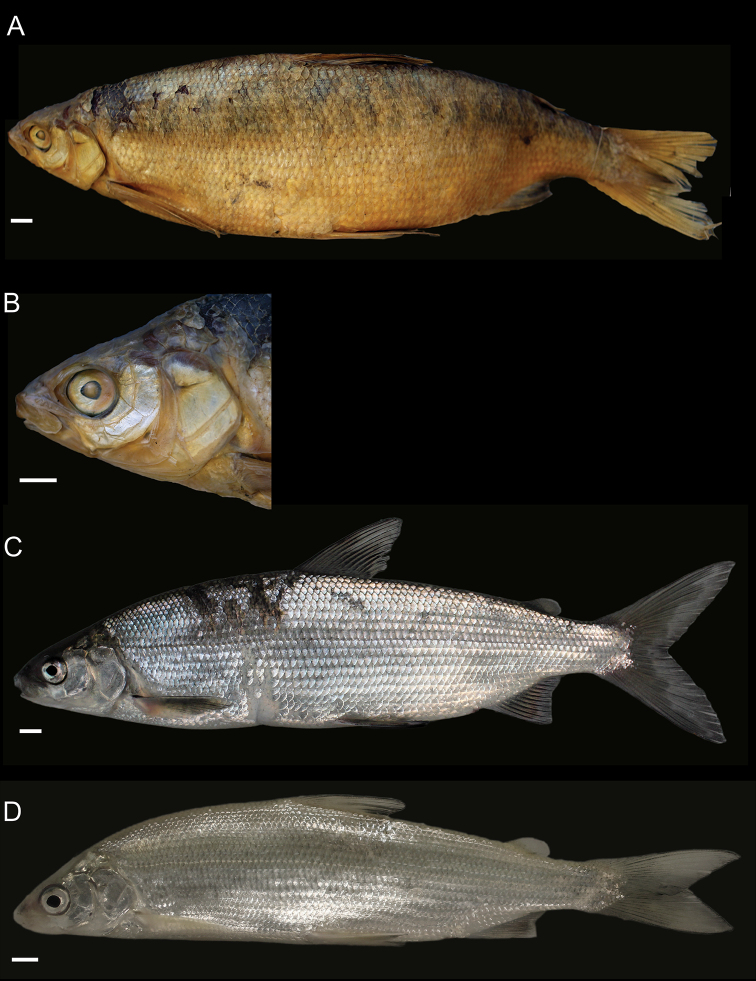
*
Coregonus alpinus*, lakes Thun and Brienz, Switzerland **A** lectotype, MHNG-717.045, Lake Thun, 283 mm SL, sex unknown **B** close-up of head of lectotype MHNG-717.045 **C** non-type, NMBE-1077246, Lake Thun, 251.5 mm SL, male, freshly caught specimen **D** non-type, NMBE-1077115, Lake Brienz, 253 mm SL, female, frozen and defrosted specimen. The white scale (1cm) below each fish acts as a reference for the actual size of the specimen.

General appearance is shown in Figure 5. Morphological and meristic characters of both sexes can be found in Table 4 and Suppl. material 1: Table S6 and first- and second-best ratios for both sexes combined can be found in Tables 10, 11. The description is valid for both sexes and both lakes; differences between the populations of lakes Thun and Brienz are mentioned.

***Shape***: Generally deep bodied with greatest body depth anterior of the dorsal fin. Dorsal profile strongly arched compared to ventral profile such that the dorsal profile from the tip of snout to the anterior origin of dorsal fin is moderate to strongly convex. Ventral profile slightly arched such that almost straight or slightly convex from the interorbital area to the pelvic fin origin. Head short. Mouth thin (i.e., width of upper and lower jaw), short and sub-terminal. Rostral plate pronounced and almost equally wide as deep resulting in an almost square shape. Tip of the snout often blunt. Small eye, which is less pronounced in specimens from Lake Brienz. Eye-socket thick and triangular (i.e., sickle-shaped). Pectoral fin moderately tapered. Dorsal fin long with the anterior unbranched ray of the erected dorsal fin approx. 60–70° angle to body axis and only slightly bent posteriorly at the end of the ray. Caudal peduncle stout and short with the caudal fin forked and sometimess lightly asymmetrical with either the ventral or dorsal part being longer. Unbranched ray of anal fin slightly bent posteriorly. Anal fin longest anteriorly and progressively shortening posteriorly with the outer margin of the anal fin mostly straight and only rarely slightly concave.

***Meristics ***: Few short gill rakers, which are shorter for specimens from Lake Brienz.

***Colour***: Pigmentation of fins and body over all strong in live specimens. In specimens from Lake Thun the pectoral fin is moderately to strongly pigmented. Dorsal, adipose, pelvic, anal and caudal fins are strongly pigmented. In specimens from Lake Brienz all fins are less pigmented. The pectoral fin is sometimes yellowish and ranges from translucent to moderately pigmented at the median to distal parts of the fin. Dorsal, adipose, pelvic, anal, and caudal fins are moderately pigmented. In both lakes fish have a silvery appearance along the flanks with few to many pigmented small dots on the scales along the flank and the dorsum (as can be found for the species of *C. fatioi*, *C. steinmanni*, *C. brienzii*). The distribution of the dots is bound to the scale patterning such that the dots are found at the edge of the scales or at the boundary point of two scales. Dorsally above the lateral line the silvery appearance changes to a light (e.g., RGB (135, 236, 179)) or darker greenish blue colour (e.g., RGB (7,168,125)). The dorsal part of the head of specimens of Lake Brienz is moderately pigmented, whereas that of specimens from Lake Thun is strongly pigmented. The snout around the nostrils is moderately (Lake Brienz) to strongly (Lake Thun) pigmented. Specimens in Lake Brienz have a gap of very weak pigmentation posteriorly of the nostrils up to the height of the middle of the eyes. The pre-operculum and operculum are silvery with one black dot on the lower margin of the pre-operculum. In some specimens of Lake Thun, the pre-operculum and operculum has some pigmented dots, similar to those found on the scales and extending also to the dorsal part of the head. For a comparison to the main colouration found in the other species see Suppl. material 1: Figure S8. Preserved specimens are pale in colouration with similar pigmentation as described for live specimens. The silvery, translucent, not coloured or unpigmented parts of the body become brown-yellowish (e.g., RGB (239, 210, 40)), whereas the pigmented parts are conserved and the coloured parts (dorsally above the lateral line) become brownish (e.g., RGB (186, 140, 100)).

##### Distribution and notes on biology.

*Coregonus alpinus* is found in the lakes Thun (46°40'N, 7°46'E) and Brienz (46°43'N, 7°57'E) that are connected through the river Aare at Interlaken. *Coregonus alpinus* feeds predominantly on benthic prey and parts of the year on zooplankton (stomach content for Lake Brienz: Maurer and Guthruf 2005; Müller et al. 2007; isotopic signature for both lakes: Selz 2008; Hudson 2011; Ingram et al. 2012) and has a rapid growth rate (Lake Brienz: Müller et al. 2007; both lakes: Kirchhofer 1995; Bittner et al. unpublished). It has to be noted that the stomach content and isotopic work did not distinguish between all species in lakes Thun or Brienz and thus in some cases lumped different species together into few groups. The stomach content work by Maurer and Guthruf (2005) and Müller et al. (2007) differentiated between "small-type" and "large-type" whitefish based on cohort-specific threshold values for length-at-age. Based on morphology and ecology Kirchhofer (1995) differentiated in Lake Thun between "Albock" (comprising most likely of *C. alpinus*, *C. steinmanni* and *C. acrinasus*), "Brienzlig" (comprising most likely of *C. albellus* and *C. fatioi*) and "Kropfer" (*C. profundus*) and in Lake Brienz between "Felchen" (comprising most likely of *C. alpinus*, *C. fatioi* and *C. brienzii*) and "Brienzlig" and "Winter-Brienzlig" (comprising of summer- and winter-spawning specimens of *C. albellus*). Finally, Selz (2008), Hudson (2011) and Ingram et al. (2012) did not yet differentiate between *C. alpinus* and *C. steinmanni* in Lake Thun, which were most likely both grouped under *C.* "Balchen". The gill raker number and length of *C. alpinus* (few and short gill rakers) suggests, based on the functional properties of the number of gill rakers experimentally tested with specimens of this species and other whitefish species from lakes Thun and Lucerne (Lundsgaard-Hansen et al. 2013; Roesch et al. 2013), that *C. alpinus* feeds more on benthic prey and less on zooplankton. However, this assumption needs to be verified with stomach content analysis that distinguish between the different species within a lake.The relative species abundances in the pelagic and benthic habitat from a habitat-stratified random sampling of Lake Thun (mid-October 2013: Vonlanthen et al. 2015) and Brienz (mid-September 2011: Vonlanthen et al. 2013) shows, that *C. alpinus* can only be found in shallow water in the benthic habitat (first 15 m; *N* = 1 each for lakes Thun and Brienz) and is completely absent from the pelagic habitat in Lake Thun, while in Lake Brienz it can be also found in the very shallow waters (approx. first 5 m; *N* = 2) of the pelagic habitat (Dönz et al. 2018). It is noteworthy that the habitat-stratified random sampling data for both lakes only covers a short period of time (one month in late summer) and it is thus not clear how the species are distributed spatially throughout the rest of the year. In Lake Thun *C. alpinus* resembles phenotypically *C. steinmanni* and to some extent *C. acrinasus*. The average size (total length) at 3 years of age for specimens in this study is 321±20 mm (mean and standard deviation, N = 8) and 273 + 14 mm (N = 4) for lakes Thun and Brienz respectively (Suppl. material 1: Figures S4–S6). The average size at 3 years of age for the specimens of *C. alpinus* from Lake Thun from this study are similar to those for the years 1969–1970 (333.8±mm, *N* = 13) and 2004–2005 (342.8±21 mm, *N* = 14) (Bittner et al. unpublished; Vonlanthen et al. unpublished). In Lake Thun the size at 3 years of age of *C. alpinus* is similar to that of *C. steinmanni*, larger than that of *C. acrinasus* and considerably larger than that of *C. albellus*, *C. fatioi* and *C. profundus* (Suppl. material 1: Figure S6). In Lake Brienz the size at 3 years of age of *C. alpinus* is larger than that of *C. fatioi* and *C. brienzii* and considerably larger than that of *C. albellus* (Suppl. material 1: Figure S6). However, the size-at-age comparisons should be treated with some caution since the sample size for *C. alpinus* is rather small. *Coregonus alpinus* has a short spawning season in late December and spawns mostly in very shallow water (1–2 m) and to a lesser extent down to 10 m and very seldom down to 30 m or more (Suppl. material 1: Figure S3; Bittner 2009; Dönz et al. 2018). *Coregonus alpinus* spawns earlier in Lake Brienz than in Lake Thun (Fatio 1890; Dönz et al. 2018). The spawning season and depth of *C. alpinus* overlaps largely with that of *C. acrinasus*, *C. fatioi* and *C. steinmanni* in Lake Thun and with that of *C. fatioi* and *C. brienzii* in Lake Brienz.

Kottelat (1997) has designated a lectotype as *C. alpinus* which is incongruent with his description of the species (with the common name "Kropfer": Kottelat (1997) and Kottelat and Freyhof (2007)). Fatio (1885, 1890) was unaware of this species (the "Kropfer") as it is not considered in his compendium of the Swiss fauna (Fatio 1890) nor in his earlier work on the Swiss whitefish (Fatio 1885). The lectotype designated by Kottelat (1997) to *C. alpinus* clearly and correctly resembles the description given by Fatio (Fatio 1885, 1890) for the species-group *Coregonus schinzii alpinus* (Fatio, 1885) and later *Coregonus schinzii helveticus* (Fatio, 1890), known then and today by its local name as "Balchen". Fatio (1890) describes the "Balchen"-type whitefish as a relatively large whitefish,with few and short gillrakers, a short and stout head with a thick and squared snout, mouth inferior and often subterminal especially for specimens from Lake Thun, a more or less small eye, caudal peduncle short and stout, long pectoral fin, all fins more or less strongly pigmented, colour of live specimens generally olive or grey-olive with greenish or blueish reflections on the back, head more or less strongly pigmented, black pigmented dots more or less abundant on the dorsum, and on the margins of the scales, spawning season in winter (November-December) and spawning depth rather shallow at the shoreline over boulders or stones (Fatio 1885: Page 663, Tables 1, 2; Fatio 1890: Pages 222–234). This description is very clearly and accurately that of a "Balchen", and very different from "Kropfer". We thus identify *C. alpinus* as the species from lakes Thun and Brienz known under the common name "Balchen". The description of *C. alpinus* in Kottelat (1997) and Kottelat and Freyhof (2007) and the photograph in Kottelat and Freyhof (2007) does not describe and depict "Balchen" but *C. profundus*, the species from Lake Thun known by the common name "Kropfer".

##### Common name.

Balchen.

#### Coregonus fatioi

Taxon classificationAnimaliaTeleosteiCoregonidae

, Kottelat, 1997

25F5D317-28F5-5A22-B2B2-1C9938656B6D

Coregonus "Albock": Heuscher 1901
Coregonus "Albock", "BRI1": Douglas et al. 1999, 2003; Douglas and Brunner 2002 (see also synonymy of C. acrinasus)
Coregonus "Felchen": Kirchhofer 1990; Kirchhofer 1995 (see also synonymy of C. alpinus and C. brienzii)
Coregonus "Large type": Maurer and Guthruf 2005; Müller et al. 2007 (see also synonymy of C. alpinus and C. brienzii)
Coregonus lavaretus wartmanni natio fatioi: Berg 1932
Coregonus lavaretus natio *arurensis*, oekot. *pelagicus*: Steinmann 1950 (see also synonymy of C. steinmanni)
Coregonus lavaretus natio *arurensis*, oekot. *primigenius*: Steinmann 1950 (see also synonymy of C. steinmanni and C. alpinus)
Coregonus "Bodenalbock", "Albock", "Schwebalbock", "Wanderalbock": Steinmann 1950 (see also synonymy of C. alpinus, C. steinmanni)
Coregonus sp. "Felchen": Hudson et al. 2011, 2013, 2016; Ingram et al. 2012
Coregonus sp. "Tiefenalbock": Vonlanthen et al. 2015
Coregonus wartmanni alpinus : Fatio 1890

##### Material examined.

***Lectotype*.** MHNG-809.059, Switzerland, Lake Thun (46°40'N, 7°46'E), 154.5 mm SL, sex unknown.

***Non-types*.** NMBE-1077133, NMBE-1077180–1077185, NMBE-1077135– 1077157, Switzerland, Lake Thun (46°40'N, 7°46'E), N = 30, 191–288 mm SL; NMBE-1077342, NMBE-1077291–1077317, NMBE-1077266, NMBE-1077267, Switzerland, Lake Brienz (46°43'N, 7°57'E), N = 30, 132–244 mm SL.

##### Diagnosis.

*Coregonus fatioi* is a medium-sized whitefish with weak pigmentation of all fins and body; light to dark green colour on the flanks above the lateral line; moderate to many pigmented small dots on the scales along the flank and the dorsum; slender, elongated and slightly torpedo-like body; long head; tip of snout is fleshy and roundish; small eye with a thin and triangular to roundish eye socket for individuals from Lake Thun and a thick and triangular shaped eye socket for individuals from Lake Brienz; many and long gill rakers.

##### Differential diagnosis.

Differential diagnoses against *C. albellus* and *C. alpinus* are given under those species’ accounts.

***Coregonus fatioi*-*Coregonus brienzii***

In Brienz *C. fatioi* can be differentiated from *C. brienzii* by being deeper bodied (22.1–26.2% SL, mean = 23.9 vs. 19.6–25.1% SL, mean = 22.6) and having a smaller eye (eye depth: 21.2–27.6% HL, mean = 24.8 vs. 23.1–28.3% SL, mean = 25.3) (Tables 5, 7, 11).

***Coregonus fatioi*-*Coregonus steinmanni***

The specimens of *C. fatioi* from Lake Thun differ from those of *C. steinmanni* by having longer gill rakers (middle gill raker length: 12.5–21.3% HL, mean = 15.8 vs. 9.1–14.3% HL, mean = 11.5; longest gill raker length: 12.8–22.6% HL, mean = 16.9 vs. 10–14.4% HL, mean = 12.1), a longer and wider underjaw (under jaw length: 28–34.1% HL, mean = 30.5 vs. 25.2–30% HL, mean = 27.3; under jaw width: 21–30.3% HL, mean = 24.7 vs. 19.3–25% HL, mean = 23). Based on ratios *C. fatioi* can be differentiated from *C. steinmanni* by having a smaller "caudal peduncle depth / upper jaw length" ratio (CD/UJ: 1.02–1.34 vs. 1.36–1.55) and "caudal peduncle depth / postdorsal length" ratio (CD/PostD: 0.14–0.17 vs. 0.17–0.20 (Tables 5, 6, 10).

***Coregonus fatioi–Coregonus profundus***

*Coregonus fatioi* from Lake Thun can be distinguished from *C. profundus* by having more and longer gill rakers (upper arch gill raker number: 10– 16, mode = 14 vs. 5–10, mode = 9; lower arch gill raker number: 22– 27, mode = 24 vs. 10–18, mode = 14; total gill raker number: 32–43, mode = 38 vs. 15–27, mode = 21; middle gill raker length: 12.5– 21.3% HL, mean = 15.8 vs. 7.6–11.7% HL, mean = 9.2; longest gill raker length: 12.8–22.6% HL, mean = 16.9 vs. 7.8–12.4% HL, mean = 10.1), shorter pectoral fin (pectoral fin 1 length: 13.3–18.9% SL, mean = 16.5 vs. 16.6–21% SL, mean = 18.4; pectoral fin 2 length: 13.8–20.6% SL, mean = 17.7 vs. 17.7–23.2% SL, mean = 20.2), a shorter head (13.6–16.2% SL, mean = 14.8 vs. 15.5–18.4% SL, mean = 16.4), a longer postdorsal length (41.6–50.7% SL, mean = 44.9 vs. 38.9–44.5% SL, mean = 42.5), and a longer upper jaw (28–34.1% HL, mean = 30.5 vs. 26.4–30.6% HL, mean = 28.7) (Tables 5, 8, Suppl. material 1: Table S6).

***Coregonus fatioi*-*Coregonus acrinasus***

*Coregonus fatioi* can be distinguished from *C. acrinasus* by having a longer postdorsal length (41.6–50.7% SL, mean = 44.9 vs. 40.3– 45.6% SL, mean = 43) and longer gill rakers (middle gill raker length: 12.5–21.3% HL, mean = 15.8 vs. 9.1–16.6% HL, mean = 13.4; longest gill raker length: 12.8–22.6% HL, mean = 16.9 vs. 11.4–16.9% HL, mean = 14.5) (Tables 5, 9).

##### Description.

**Figure 6.**
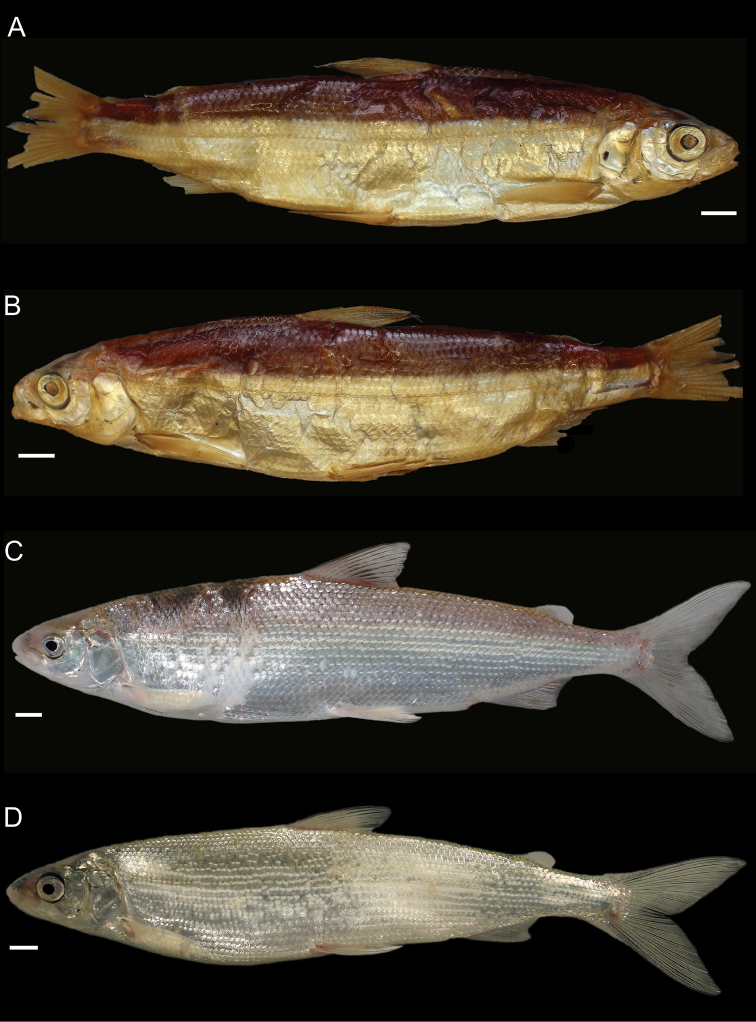
*
Coregonus fatioi*, lakes Thun and Brienz, Switzerland **A, B** lectotype, MHNG-809.059, Lake Thun, 154.5 mm SL, sex unknown, left and right side of the specimen **C** non-type, NMBE-1077139, Lake Thun, 240 mm SL, male, freshly caught specimen **D** non-type, NMBE-1077317, Lake Brienz, 202 mm SL, male, frozen and defrosted specimen. The white scale (1 cm) below each fish acts as a reference for the actual size of the specimen.

General appearance is shown in Figure 6. Morphological and meristic characters of both sexes can be found in Table 5 and Suppl. material 1: Table S6 and first- and second-best ratios for both sexes combined can be found in Tables 10, 11. The description is valid for both sexes and both lakes; differences between the populations of lakes Thun and Brienz are mentioned.

***Shape***: Elongated. Slender bodied with greatest body depth anterior of the dorsal fin resulting in a slightly torpedo-like form. Dorsal and ventral profile similar and slightly arched. Dorsal and ventral profile from tip of snout to interorbital area mostly straight and then slightly convex to dorsal and pelvic fin origin respectively. Head long. Very rarely does the snout have an approx. 40–50° angle to the body axis anterior of the eye, such that the profile from the tip of the snout to the vertical projection where the anterior part of the eye crosses the dorsal profile is straight and afterwards slightly convex. Mouth thick (i.e., width of upper and lower jaw), long and often terminal and only rarely slightly sub-terminal. Snout mostly wider than deep, not strongly pronounced, since the tip of the snout is often fleshy and roundish. Specimens from Lake Thun have a thin, roundish and rarely triangular shaped eye-socket, whereas specimens from Lake Brienz have an eye-socket that is thick and triangular (i.e., sickle-shaped). Pectoral fin moderately tapered. Anterior unbranched ray of the erected dorsal fin ranges from almost vertically straight to an approx. 60–80° angle to body axis and only bent slightly posteriorly at the end of the ray. Caudal peduncle narrow and elongated with caudal fin forked in specimens from both lakes and sometimes moderately asymmetrical (mostly the ventral part is longer) in specimens from Lake Thun but very rarely in specimens from Lake Brienz. Unbranched ray of anal fin straight and rarely bent posteriorly at the end of the ray. Anal fin longest anteriorly and progressively shortening posteriorly with the outer margin of the anal fin ranging from being straight to slightly concave.

***Meristics ***: Specimens of Lake Thun have many and long gill rakers, whereas specimens from Lake Brienz have a bit less and moderately long gill rakers.

***Colour***: Pigmentation of fins and body overall weak in live specimens. In specimens from Lake Thun the pectoral fin is translucent, sometimes yellowish with faint pigmentation at the median to distal parts of the fin. In Thun the pelvic fin ranges from completely translucent to moderately pigmented and the dorsal, adipose, anal and caudal fins are moderately pigmented. Specimens from Lake Brienz have a fully translucent pectoral fin that sometimes has a faint pigmentation on the unbranched ray. Pelvic and anal fins range from fully transparent to moderately pigmented and dorsal, adipose and caudal fins are moderately pigmented. In both lakes fish have a silvery appearance along the flanks. Specimens from both lakes sometimes have many pigmented small dots on the scales along the flank and the dorsum, which is rare in specimens from Lake Thun and common in specimens from Lake Brienz. Distribution of the dots is bound to the scale patterning such that the dots are found at the edge of the scales or at the boundary point of two scales (as can be found for the species of *C. alpinus*, *C. brienzii* and *C. steinmanni*). Colouration on the dorsum above the lateral line of specimens from Lake Thun ranges from a light green colouration (e.g., RGB (136, 245, 205)) to an olive-green colouration (e.g., RGB (176, 192, 125)), where the former is more common. In specimens from Lake Brienz the upper dorsum is light greenish in colouration (e.g., RGB (136, 245, 205)). For a comparison to the main colouration found in the other species see Suppl. material 1: Figure S8. The dorsal part of the head of specimens of Lake Brienz is weakly pigmented, whereas that of specimens from Lake Thun is moderately pigmented. The snout around the nostrils is weakly (Lake Brienz) to moderately (Lake Thun) pigmented with a gap of very weak (Brienz) to moderate (Thun) pigmentation posteriorly of the nostrils up to the height of the middle of the eyes. Operculum and pre-operculum are silvery with one black dot on the lower margin of the pre-operculum. Preserved specimens are pale in colouration with similar pigmentation as described for live specimens. Silvery, translucent, not coloured or unpigmented parts of the body become brown-yellowish (e.g., RGB (239, 210, 40)), whereas the pigmented parts are conserved and the coloured parts (dorsally above the lateral line) become brownish (e.g., RGB (186, 140, 100)).

##### Distribution and notes on biology.

*Coregonus fatioi* is found in the lakes Thun (46°40'N, 7°46'E) and Brienz (46°43'N, 7°57'E) that are connected through the river Aare at Interlaken. Based on isotopic signatures *C. fatioi* feeds predominantly on zooplankton (Selz 2008; Hudson 2011; Ingram et al. 2012). Stomach content analyses of specimens of *C. fatioi* from Lake Brienz suggest that *C. fatioi* feeds on a mix of zooplankton and benthic prey (Maurer and Guthruf 2005; Müller et al. 2007). *Coregonus fatioi* has a moderately fast growth rate (Both lakes: Kirchhofer 1995; Bittner et al. unpublished; Lake Brienz: Müller et al. 2007). It has to be noted that the work by Kirchhofer (1995), Maurer and Guthruf (2005) and Müller et al. (2007) did not distinguish between all species in lakes Thun or Brienz and thus lumped different species together into few groups. Maurer and Guthruf (2005) and Müller et al. (2007) differentiated between "small-type" and "large-type" whitefish based on cohort-specific threshold values for length-at-age. Based on morphology and ecology Kirchhofer (1995) differentiated in Lake Thun between "Albock" (comprising most likely of *C. alpinus*, *C. steinmanni* and *C. acrinasus*), "Brienzlig" (comprising most likely of *C. albellus* and *C. fatioi*) and "Kropfer" (*C. profundus*) and in Lake Brienz between "Felchen" (comprising most likely of *C. alpinus*, *C. fatioi* and *C. brienzii*) and "Brienzlig" and "Winter-Brienzlig" (comprising of summer- and winter-spawning specimens of *C. albellus*). The gill raker number and length of *C. fatioi* (many and long gill rakers) suggests, based on the functional properties of the number of gill rakers (Lundsgaard-Hansen et al. 2013; Roesch et al. 2013), that *C. fatioi* feeds more on zooplankton and less on benthic prey. However, this assumption needs to be verified with stomach content analyses that distinguish between the different species within a lake. Habitat stratified random sampling of Lake Thun (mid-October 2013; Vonlanthen et al. 2015) and Brienz (mid-September 2011; Vonlanthen et al. 2013) shows, that *C. fatioi* occupies the moderately shallow (Brienz: approx. 1–48 m, *N* = 9; Thun: approx. 25–140 m, *N* = 4) to the deepest waters of the benthic habitat in both lakes (down to 217 m and 261 m in lakes Thun and Brienz, respectively) (Dönz et al. 2018). In the pelagic habitat *C. fatioi* aggregates in lakes Thun and Brienz in moderate water depths (Brienz: approx. 1–100 m, *N* = 10; Thun: approx. 10–40 m, *N* = 9) (Dönz et al. 2018). Note that the habitat-stratified random sampling data for both lakes only covers a short period of time (one month in late summer) and it is thus not clear how the species are distributed spatially through the rest of the year. Furthermore, the habitat-stratified random sampling in the both lakes did not distinguish between ripe and unripe specimens, and thus in the case of *C. fatioi* the distribution pattern along the depth in the benthic zone is biased by the spawning aggregation of this species since the sampling period in both lakes coincides partially with the spawning season of this species. *Coregonus fatioi* resembles phenotypically *C. albellus* and to some extent *C. profundus*. Interestingly, Steinmann (1950) already mentioned for Lake Thun that the ecotype "*Coregonus lavaretus L. nat. arurenis*, *oekot. nanus*" (most likely *C. albellus*) should be grouped based on its ecology closely to the ecotype "*Coregonus lavaretus L. nat. arurenis*, *oekot. pelagicus*" (most likely *C. fatioi*). Steinmann mentions the German name "Schwebalbock" for the ecotype "pelagicus", which means verbally translated the "floating whitefish" and mentions that the "nanus" ecotype seems to be a small species with similar ecological properties. For the large whitefish species in Lake Thun, Steinmann (1950) defined one central ecotype, the "primigenius" ecotoype, which he places – based on the size – with two other ecotypes namely the "litoralis" ecotoype (most likely *C. alpinus*) and the "pelagicus" ecotype (most likely *C. fatioi*). Besides referring to a "primigenius" ecotype, Steinmann (1950) also refers to a "primigenius"-group, which most likely comprises of the "pelagicus" and "litoralis" ecotypes. A further indication of this is that he also mentions that yet another ecotype, namely the "profundus" ecotype, can be directly deduced from the "primigenius" ecotype. Steinmann (1950) further mentions that specimens, which he places in the "primigenius"-group, used to migrate before the construction of water gates (see below) upstream from Lake Thun into the river Aare, which connects Lake Thun with Lake Brienz. Steinmann (1950) mentions that these fish belong to the "primigenius"-group, but did not specify if the migrating population constituted of individuals of the "litoralis" or the "pelagicus" ecotype or both. This migrating population was referred to as "Wanderalbock" (i.e. migrating whitefish) in German and historically migrated from Lake Thun into Lake Brienz during the spawning season, before migration became impossible due to the construction of water gates in 1856 (Fatio 1890; Dönz et al. 2018). Fatio (1890) mentioned that a large part of the population of *C. fatioi* "disappeared" at the beginning of the spawning season in late August and was caught by fishermen in the river Aare downstream (near the city of Thun or Bern) or upstream (near the city of Interlaken) of Lake Thun before and after the construction of the water gate. We compared six whitefish specimens from the museum collections of the MHNG and NMBE, which had no species designation but where it was mentioned that they were caught in the river Aare near the city of Bern (in the years 1881 and 1895), Thun (in the year 1950) and Interlaken (in the year 1945), to the contemporary specimens of Lake Thun including the type specimens of *C. albellus*, *C. fatio*i and *C. alpinus*. All the specimens were caught after the construction of the water gate, when free movement between the lakes was already constrained. All six specimens from the river Aare group in morphospace within the range or adjacent to the range of the contemporary specimens of *C. fatioi* including the type specimen (Suppl. material 1: Figure S11a–c), suggesting that the historically migrating population of whitefish from Lake Thun most likely belonged to the species *C. fatioi*. Bittner (2009) sampled and genotyped individuals of a population of whitefish spawning in the river Aare near Interlaken. Dönz et al. (2018) re-analysed those individuals and was able to assign 4 individuals with high assignment probability (>70%) to several different contemporary species of Lake Thun, namely *C. alpinus* (individual assignment probability of THL15N18 = 86%), *C. acrinasus* (ind. assign. prob. of THL15N07 and THL15N23 = 77% and 80%, respectively) and *C. fatioi* (ind. assign. prob. of THL15NfS1124 = 92%). This suggest either that historically more species than just *C. fatioi* migrated to the river Aare for spawning and were missed both by Fatio (1890) and Steinmann (1950) and are thus not represented in our PCA morphospace of Aare river whitefish (Suppl. material 1: Figure S11 a–c). Or the historical migratory population consisted – as has been suggested by Fatio (1890) and Steinmann (1950) – of individuals of *C. fatioi*. The average size (total length) at 3 years of age for specimens in this study is 266±15 mm (mean and standard deviation, N = 14) and 244±14 mm (N = 16) for lakes Thun and Brienz respectively (Suppl. material 1: Figures S4–S6). In Lake Brienz the size of 3-year-old specimens of *C. fatioi* is considerably larger than that of *C. albellus* and similar to that of *C. alpinus* and *C. brienzii*, whereas in Lake Thun it is similar to that of *C. profundus* and *C. albellus* and smaller than that of *C. alpinus*, *C. steinmanni*, and *C. acrinasus* (Suppl. material 1: Figure S6). *Coregonus fatioi* has a long spawning season with two peaks. One spawning peak is in late summer/early autumn from August to October, which seems more common in Lake Thun than Lake Brienz, and the second peak is in early to late winter from December to March (Suppl. material 1: Figure S3; Bittner 2009; Dönz et al. 2018). Spawning depth varies with spawning season and can range from approx. 40 m down to the max. depth of 210 m and 261 m in lakes Thun and Brienz, respectively (Suppl. material 1: Figure S3; Bittner 2009; Dönz et al. 2018). Occasionally *C. fatioi* can be found spawning shallower (up to 10 m), but generally it spawns in deeper waters. The spawning season and depth of *C. fatioi* partially overlaps with that of *C. steinmanni*, *C. albellus*, C. acrinasus, and *C. profundus* in Lake Thun and with that of *C. albellus* and *C. brienzii* in Lake Brienz.

##### Etymology.

The name given to this species by Fatio (1890) was preoccupied by another species described by Fatio (1885). Kottelat (1997) proposed *C. fatioi* as a replacement name. The specific epithet *fatioi* is the genitive of *Fatio*. It was named by Kottelat (1997) after the late researcher Viktor Fatio, a zoologist from Switzerland who wrote a standard reference work on the Swiss vertebrates entitled "Faune des Vertébrés de la Suisse Partie 1–3"and in which he also described part of the whitefish species diversity of Switzerland.

##### Common name.

Tiefenalbock in Lake Thun and Felchen in Lake Brienz.

#### Coregonus steinmanni

Taxon classificationAnimaliaTeleosteiCoregonidae

Selz, Dönz, Vonlanthen & Seehausen sp. nov.

193D6FF5-D17A-5D00-81E7-D511DA826487

http://zoobank.org/C03A9DA8-8492-4CBF-B87B-406D72594530

Coregonus "Albock": Rufli 1978, 1979; Kirchhofer and Tschumi 1986; Kirchhofer 1995 (see also synonymy of C. alpinus and C. acrinasus)
Coregonus "Balchen": Heuscher 1901; Surbeck 1917 (see also synonymy of C. alpinus)
Coregonus "Balchen", "THU2": Douglas et al. 1999, 2003; Douglas and Brunner 2002 (see also synonymy of C. alpinus)
Coregonus lavaretus natio *arurensis*, oekot. *primigenius*: Steinmann 1950 (see also synonymy of C. fatio and C. alpinus)
Coregonus sp. "Balchen": Hudson et al. 2011, 2013, 2016; Ingram et al. 2012; Vonlanthen et al. 2012, 2015; Vonlanthen and Périat 2013 (see also synonymy of C. alpinus and C. brienzii)
Coregonus sp. "Balchen 2": Dönz et al. 2018 (see also synonymy of C. brienzii)
Coregonus "Wanderalbock", "Bodenalbock", "Albock": Steinmann 1950 (see also synonymy of C. alpinus, C. fatioi, C. steinmanni and C. brienzii)

##### Material examined.

***Holotype*.** NMBE-1077219, Switzerland, Lake Thun (46°40'N, 7°46'E), 301 mm SL, female.

***Paratypes*.** NMBE-1077132, NMBE-1077212–1077218, NMBE-1077220, NMBE-1077262–1077265, Switzerland, Lake Thun (46°40'N, 7°46'E), N = 13, 211–323 mm SL.

##### Diagnosis.

*Coregonus steinmanni* is a large whitefish with moderate pigmentation of all fins and body; light to dark greenish blue colour on the flanks above the lateral line; moderate to many pigmented small dots on the scales along the flank and the dorsum; deep bodied; stout caudal peduncle; short head; sub-terminal mouth; small eye with a thick and triangular shaped eye socket.

##### Differential diagnosis.

*Coregonus steinmanni* occurs only in Lake Thun and we therefore compare the characters of this species specifically with the species of Lake Thun. Differential diagnoses against *C. albellus*, *C. alpinus*, and *C. fatioi* are given under those species’ accounts.

***Coregonus steinmanni*-*Coregonus profundus***

*Coregonus steinmanni* can be distinguished from *C. profundus* by having more and longer gill rakers (upper arch gill raker number: 10–12, mode = 11 vs. 5–10, mode = 9; lower arch gill raker number: 19–23, mode = 20 vs. 10–18, mode = 14; total gill raker number: 30–35, mode = 31 vs. 15–27, mode = 21; middle gill raker length: 9.1–14.3% HL, mean = 11.5 vs. 7.6–11.7% HL, mean = 9.2; longest gill raker length: 10–14.4% HL, mean = 12.1 vs. 7.8–12.4% HL, mean = 10.1), shorter pectoral fin (pectoral fin 1 length: 13.9–18.2% SL, mean = 16.2 vs. 16.6–21% SL, mean = 18.4; pectoral fin 2 length: 15.2–19.1% SL, mean = 17 vs. 17.7–23.2% SL, mean = 20.2), a shorter head (13.2–15.1% SL, mean = 14 vs. 15.5– 18.4% SL, mean = 16.4), a smaller eye cavity (24.2–27.8% HL, mean = 26.2 vs. 26.2–32.1% HL, mean = 29.2), a narrower underjaw (19.3– 25, mean = 23% HL vs. 22.7–29.2% HL, mean = 26), and a shorter prepelvic distance (48.6–54.3% SL, mean = 51.7 vs. 51.2–58.1% SL, mean = 54.2). Based on ratios *C. steinmanni* can be differentiated from *C. profundus* by having a larger "caudal fin depth / dorsal head length" ratio (0.53–0.63 vs. 0.4–0.49) (Tables 6, 8, 10, Suppl. material 1: Table S6).

***Coregonus steinmanni*-*Coregonus acrinasus***

*Coregonus steinmanni* differs from *C. acrinasus* by having a shorter maxilla (18.1–21.8% HL, mean = 19.7 vs. 19.4–23.8% HL, mean = 21.8) (Tables 6, 8) and can be differentiated based on ratios from *C. acrinasus* by having a larger "caudal peduncle depth / maxilla length" ratio (1.86–2.24 vs. 1.4–1.9) (Tables 6, 9, 10).

##### Description.

**Figure 7.**
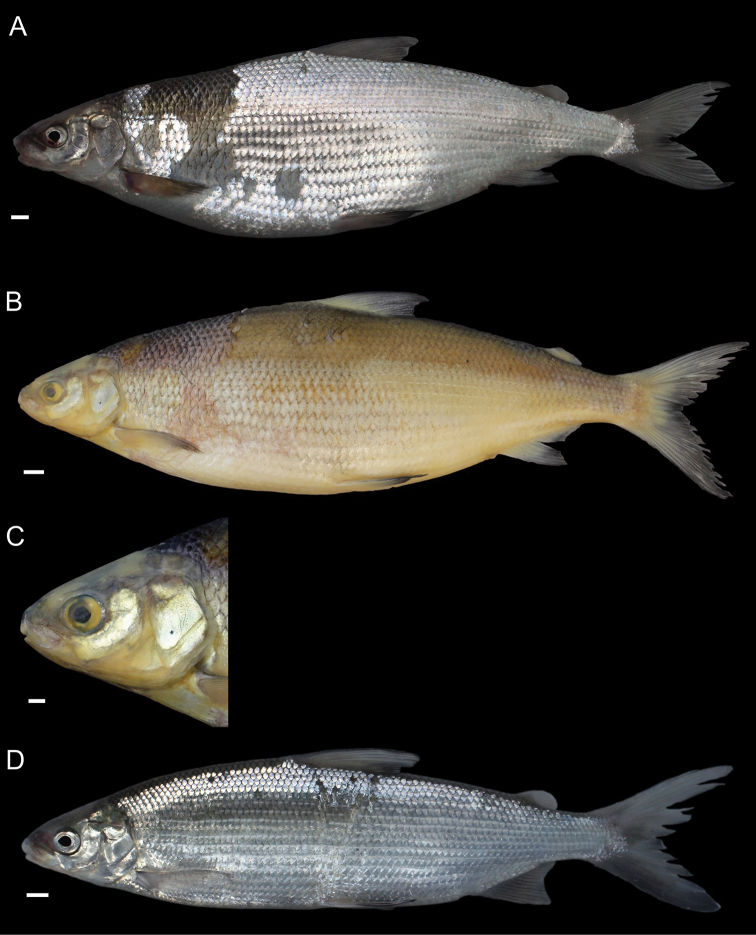
*
Coregonus steinmanni*, Lake Thun, Switzerland **A** holotype, NMBE-1077219, Lake Thun, 301 mm SL, female, freshly caught specimen **B, C** NMBE-1077219, holotype, preserved specimen **D** paratype, NMBE-1077214, Lake Thun, 234 mm SL, female, freshly caught specimen. The white scale (1cm) below each fish acts as a reference for the actual size of the specimen.

General appearance is shown in Figure 7. Morphological and meristic characters of both sexes can be found in Table 6 and Suppl. material 1: Table S6 and first- and second-best ratios for both sexes combined can be found in Table 10. The description is valid for both sexes.

***Shape***: Generally deep bodied with greatest body depth anterior of the dorsal fin. Dorsal profile strongly arched compared to ventral profile. Dorsal profile from the tip of snout to the anterior origin of dorsal fin moderate to strongly convex, whereas the ventral profile is slightly arched such that it is almost straight or slightly convex from the interorbital area to the pelvic fin origin. Mouth is rather thin (i.e., width of upper and lower jaw), short and sub-terminal. Snout is pronounced and almost equally wide as deep resulting in an almost square shape. Small eye. Eye-socket is thick and triangular (i.e., sickle-shaped). Pectoral fin moderately tapered. The anterior unbranched ray of the erected dorsal fin has an approx. 60° angle to body axis and at the end of the ray it is bent posteriorly. Caudal peduncle is stout and short. Caudal fin forked and sometimes slightly asymmetrical with the dorsal part being longer. Un-branched ray of anal fin mostly straight and only sometimes slightly bent posteriorly. Anal fin longest anteriorly and progressively shortening posteriorly with the outermargin of the anal fin mostly slightly concave and only rarely straight.

***Meristics ***: Few and short gill rakers.

***Colour***: Pigmentation of fins and body overall moderately strong in live specimens. Pectoral fin is moderately to strongly pigmented. Dorsal, adipose, pelvic, anal, and caudal fins are moderately to strongly pigmented. Silvery appearance along the flanks with moderate to many pigmented small dots on the scales. The dots are found along the flank and the dorsum. Distribution of the dots is bound to the scale patterning such that the dots are found at the edge of the scales or at the boundary point of two scales (as can be found for the species of *C. fatioi*, *C. alpinus* and *C. brienzii*). Dorsally above the lateral line the silvery appearance changes to a light (e.g., RGB (135, 236, 179)) or darker greenish blue colour (e.g., RGB (7,168,125)). Dorsal part of the head strongly pigmented. Snout around the nostrils strongly pigmented with a gap of moderate pigmentation posteriorly of the nostrils up to the height of the middle of the eyes. Pre-operculum and operculum are silvery with one black dot on the lower margin of the pre-operculum. For a comparison to the main colouration found in the other species see Suppl. material 1: Figure S8. Preserved specimens are pale in colouration with similar pigmentation as described for live specimens. Silvery, translucent, not coloured or unpigmented parts of the body become brown-yellowish (e.g., RGB (239, 210, 40)), whereas the pigmented parts are conserved and the coloured parts (dorsally above the lateral line) become brownish (e.g., RGB (186, 140, 100)).

##### Distribution and notes on biology.

*Coregonus steinmanni* is found in Lake Thun (46°40'N, 7°46'E), which is connected to Lake Brienz through the river Aare at Interlaken. Based on isotopic signatures *C. steinmanni* feeds on a mix of benthic prey and zooplankton (Selz 2008; Hudson 2011; Ingram et al. 2012) and has a fast growth rate (Bittner 2009). It has to be noted that the work by Selz (2008), Hudson (2011) and Ingram et al. (2012) did not yet separate *C. alpinus* from *C. steinmanni*, which are phenotypically difficult to distinguish. Only recently has genetic work by Dönz and colleagues (2018) clearly resolved that these are two distinct species. Thus, the isotopic work by Selz (2008), Hudson (2011) and Ingram et al. (2012) most likely comprises of specimens of both species. The gill raker number of *C. steinmanni* (more gill rakers) and *C. alpinus* (fewer gill rakers) suggests – based on the functional properties of the number of gill rakers on feeding on different prey items (Lundsgaard-Hansen et al. 2013; Roesch et al. 2013) – that *C. steinmanni* feeds more on zooplankton and less on benthic prey than *C. alpinus*, but this assumption needs to be verified in the future with stomach content analyses. Interestingly, the relative species abundances in the pelagic and benthic habitat from a habitat stratified random sampling in Lake Thun (mid-October 2013: Vonlanthen et al. 2015) shows, that *C. steinmanni* is occupying the moderately deep waters of the benthic habitat (76 m; *N* = 1) and the shallow waters of the pelagic habitat (8 m; *N* = 1) (Dönz et al. 2018). *Coregonus alpinus* on the other hand can exclusively be found in shallow water in the benthic habitat (first 13 m; *N* = 1) and is completely absent from the pelagic habitat in Lake Thun (Dönz et al. 2018). It is to note that the habitat-stratified random sampling data only covers a short period of time (one month in late summer) and it is thus not clear how the species are distributed spatially through the rest of the year.

*Coregonus steinmanni* resembles phenotypically *C. alpinus* and to some extent *C. acrinasus*. The average size (total length) at 3 years of age for specimens in this study is 328±23 mm (mean and standard deviation, N = 11) (Suppl. material 1: Figures S4–S6). The average size at 3 years of age for the specimens of *C. steinmanni* from this study is similar to that for the years 2004–2005 (338.5±19 mm, *N* = 8) (Bittner et al. unpublished; Vonlanthen et al. unpublished). The size of 3-year-old specimens of *C. steinmanni* is similar to that of *C. alpinus*, larger than that of *C. acrinasus* and considerably larger than that of *C. albellus*, *C. fatioi* and *C. profundus* (Suppl. material 1: Figure S6). *Coregonus steinmanni* has a short spawning season in late December and only rarely can be found spawning in late autumn (Suppl. material 1: Figure S3; Dönz et al. 2018). *Coregonus steinmanni* spawns mostly in moderately shallow waters of 10 m down to approx. 120 m (Suppl. material 1: Figure S3; Bittner 2009; Dönz et al. 2018). The spawning season and depth of *C. steinmanni* overlaps largely with that of *C. acrinasus* and *C. alpinus* and partially with that of *C. fatioi*. To a much lesser extent the spawning depth and time of *C. steinmanni* also overlaps with that of *C. albellus* and *C. profundus*.

##### Etymology.

The specific epithet *steinmanni* is the genitive of *Steinmann*. We name this species after the high school teacher and researcher Paul Steinmann, a zoologist from Switzerland who wrote the most comprehensive compendium on Swiss whitefish to date and compiled throughout his lifetime a large collection of preserved specimens of Swiss, but also European, fishes (Steinmann 1950). This collection and his work on the revision of Swiss whitefish together with work by Fatio (1890) has been essential to describe the whitefish diversity that was present in Switzerland just before or at the beginning of the strong anthropogenic-induced eutrophication of many Swiss lakes which was accompanied by population collapse, speciation reversals, and extinction of Swiss whitefish (Vonlanthen et al. 2012). For example, the only existing specimens of a now-extinct whitefish species, *C. gutturosus* Gmelin 1818, can only be found in the collection of Paul Steinmann.

##### Common name.

None; this species was not recognized by local fishermen or fisherwomen as distinct from *C. alpinus* and was thus also called "Balchen". We suggest the German name "Steinmann’s Balchen".

#### C. brienzii

Taxon classificationAnimaliaTeleosteiCoregonidae

Selz, Dönz, Vonlanthen & Seehausen sp. nov.

372C01C3-636C-5ECD-9A70-8ED8FB1B139B

http://zoobank.org/C42663B8-4D34-4499-85D9-259AB7DA204B

Coregonus "Felchen": Kirchhofer 1990; Kirchhofer 1995 (see also synonymy of C. alpinus and C. fatioi)
Coregonus "Large type": Maurer and Guthruf 2005; Müller et al. 2007 (see also synonymy of C. fatioi and C. alpinus)
Coregonus sp. "Balchen": Hudson et al. 2011, 2013, 2016; Ingram et al. 2012; Vonlanthen et al. 2012, 2015; Vonlanthen and Périat 2013 (see also synonymy of C. alpinus and C. steinmanni)
Coregonus sp. "Balchen 2": Dönz et al. 2018 (see also synonymy of C. steinmanni)

##### Material examined.

***Holotype*.** NMBE-1077126, Switzerland, Lake Brienz (46°43'N, 7°57'E), 223 mm SL, female.

***Paratypes*.** NMBE-1077116–1077125, NMBE-1077127–1077128, Switzerland, Lake Brienz (46°43'N, 7°57'E), N = 12, 118–226 mm SL.

##### Diagnosis.

*Coregonus brienzii* is a medium-sized whitefish with moderate pigmentation of all fins and body; light to dark greenish blue colour on the flanks above the lateral line; moderate to many pigmented small dots on the scales along the flank and the dorsum; deep bodied; stout caudal peduncle; short head; moderately large eye with a moderately thick and triangular shaped eye socket.

##### Differential diagnosis.

*Coregonus brienzii* occurs only in Lake Brienz and we therefore compare the characters of this species specifically with the species of Lake Brienz. Differential diagnoses against *C. albellus*, *C. alpinus*, and *C. fatioi* are given under those species’ accounts.

##### Description.

**Figure 8.**
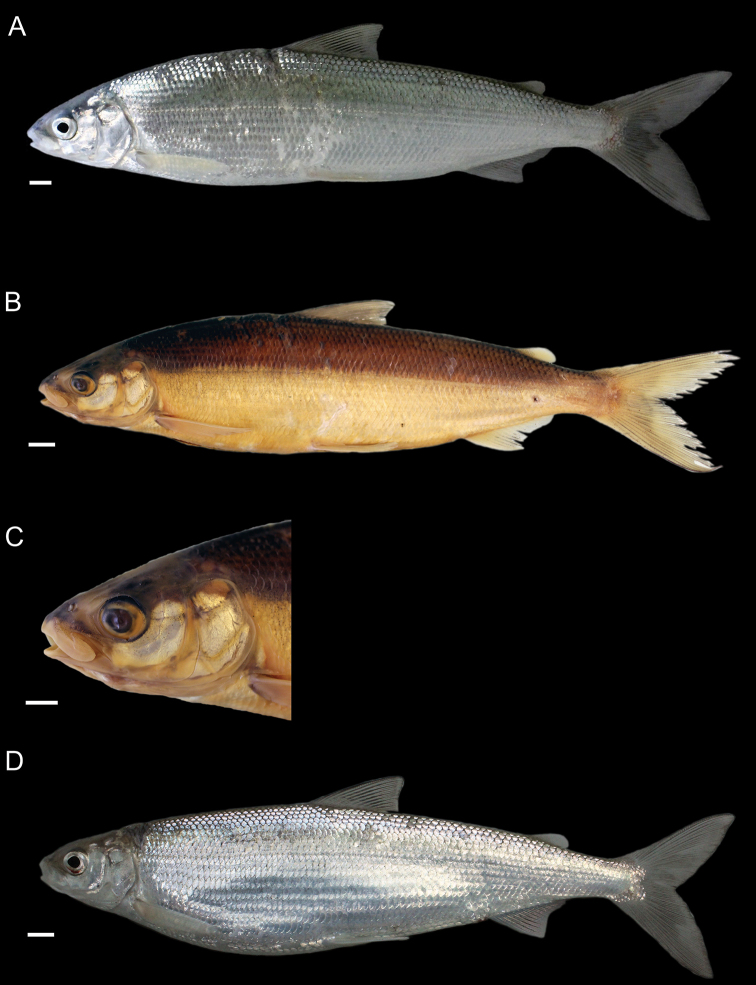
*
Coregonus brienzii*, Switzerland, Lake Brienz **A** holotype, NMBE-1077126, 223 mm SL, female, freshly caught specimen **B, C** holotype, NMBE-1077126, preserved specimen **D** paratype, NMBE-1077116, 210.5 mm SL, female.

General appearance is shown in Figure 8. Morphological and meristic characters of both sexes can be found in Table 7 and Suppl. material 1: Table S6 and first- and second-best ratios for both sexes combined can be found in Table 11. The description is valid for both sexes.

***Shape***: Moderately deep bodied with greatest body depth anterior of the dorsal fin. Dorsal profile moderately arched compared to ventral profile. The dorsal profile from the tip of snout to the anterior origin of dorsal fin is moderately convex, whereas the ventral profile is slightly arched such that is almost straight or slightly convex from the interorbital area to the pelvic fin origin. In some specimens the ventral profile and dorsal profile are similar and only slightly arched. Head moderately short. Mouth is rather thin (i.e., width of upper and lower jaw), moderately short and terminal to sub-terminal. The snout can range from almost equally wide as deep to wider than deep, and is only moderately pronounced, since the tip of the snout can sometimes be fleshy and roundish. Moderately large eye. The eye-socket is thick and triangular (i.e., sickle-shaped). Pectoral fin moderately tapered. The anterior unbranched ray of the erected dorsal fin is almost vertically straight with an approx. 70–80° angle to the body axis and is only bent slightly posteriorly at the end of the ray. Caudal peduncle is moderately stout and short. Caudal fin forked and sometimes slightly asymmetrical with the dorsal part being longer. Unbranched ray of anal fin mostly straight and only sometimes slightly bent posteriorly. Anal fin longest anteriorly and progressively shortening posteriorly with the outer margin of the anal fin mostly slightly concave and only rarely straight.

***Meristics ***: Many gill rakers that are moderately long.

***Colour***: Pigmentation of fins and body overall moderate in live specimens. The pectoral fin is mostly translucent and only rarely moderately pigmented at the median to distal parts of the fin. The dorsal, adipose, pelvic, anal, and caudal fins are moderately pigmented. Silvery appearance along the flanks with moderate to many pigmented small dots on the scales. The dots are found along the flank and the dorsum. The distribution of the dots is bound to the scale patterning such that the dots are found at the edge of the scales or at the boundary point of two scales (as can be found for the species of *C. alpinus* and *C. fatioi* from both lakes and *C. steinmanni* from Lake Thun). Dorsally above the lateral line the silvery appearance changes to a light (e.g., RGB (135, 236, 179)) or darker greenish blue colour (e.g., RGB (7,168,125)). The dorsal part of the head is moderately pigmented. The snout around the nostrils is moderately pigmented with a gap of very weak pigmentation posteriorly of the nostrils up to the height of the middle of the eyes. The pre-operculum and operculum are silvery with one black dot on the lower margin of the pre-operculum. For a comparison to the main colouration found in the other species see Suppl. material 1: Figure S8. Preserved specimens are pale in colouration with similar pigmentation as described for live specimens. The silvery, translucent, not coloured or unpigmented parts of the body become brown-yellowish (e.g., RGB (239, 210, 40)), whereas the pigmented parts are conserved and the coloured parts (dorsally above the lateral line) become brownish (e.g., RGB (186, 140, 100)).

##### Distribution and notes on biology.

*Coregonus brienzii* is found in Lake Brienz (46°43'N, 7°57'E) which is connected with Lake Thun through the river Aare at Interlaken. Our previous genetic work (Dönz et al. 2018) suggested that *C. brienzii* is the same species as *C. steinmanni* and that it together with the other three species, *C. alpinus*, *C. fatioi*, and *C. albellus*, is present in both lakes. All four species displayed the same genetic relationships in both lakes (i.e., the same hierarchical grouping into distinct genotypic clusters and similar extends of genetic divergence). However, recent analyses of whole-genome data (De-Kayne et al. unpublished) revealed, that specimens of *C. steinmanni* from Lake Thun do not group with those of *C. brienzii*, whereas those of the other three species from both lakes do cluster together. Instead the whole genome data suggests that *C. steinmanni* clusters closer to *C. alpinus* from Lake Thun – as has previously been shown with genetic data (Dönz et al. 2018) – and that *C. brienzii* clusters closer to *C. fatioi* from Lake Brienz. Interestingly, we also find morphological relationships to differ between the lakes; in Lake Thun *C. steinmanni* groups in morphospace with *C. alpinus*, whereas in Lake Brienz *C. brienzii* groups in morphospace with *C. fatioi*.

*Coregonus brienzii* most likely feeds on a mix of benthic prey and zooplankton (stomach content: Maurer and Guthruf 2005; Müller et al. 2007; isotopic signatures: Selz 2008; Hudson 2011) and has a moderatly fast growth rate (Müller et al. 2007). It has to be noted that the work by Kirchhofer (1995), Maurer and Guthruf (2005) and Müller et al. (2007) did not distinguish between all species in Lake Brienz and thus lumped different species together into few groups. Maurer and Guthruf (2005) and Müller et al. (2007) differentiated between "small-type" and "large-type" whitefish based on cohort-specific threshold values for length-at age. Based on morphology and ecology Kirchhofer (1995) differentiated in Lake Brienz between "Felchen" (comprising most likely of *C. alpinus*, *C. fatioi* and *C. brienzii*) and "Brienzlig" and "Winter-Brienzlig" (comprising of summer- and winter-spawning specimens of *C. albellus*). Also, the isotopic work by Selz (2008), and Hudson (2011) did not yet differentiate between *C. fatioi* and *C. brienzii*. The relative species abundances in the pelagic and benthic habitat from a habitat-stratified random sampling of Lake Brienz (mid-September 2011: Vonlanthen et al. 2013) shows, that *C. brienzii* is absent from the benthic habitat and is present in the moderately deep pelagic waters (30 m; *N* = 1) (Dönz et al. 2018). It is to note that the habitat-stratified random sampling data only covers a short period of time (one month in late summer) and it is thus not clear how the species is distributed spatially through the rest of the year. *Coregonus brienzii* resembles phenotypically *C. fatioi*. The average size (total length) at 3 years of age for specimens in this study is 254 + 14 mm (N = 8) (Suppl. material 1: Figures S5, S6). The size at 3 years of age of *C. brienzii* is similar to that of *C. fatioi*, slightly smaller than that of *C. alpinus* and considerably larger than that of *C. albellus* (Suppl. material 1: Figure S6). *Coregonus brienzii* has a short spawning season in late December (Suppl. material 1: Figure S3; Dönz et al. 2018). *Coregonus brienzii* spawns mostly in moderately shallow waters of 10 m down to 60 m and rarely to 100 m (Suppl. material 1: Figure S3; Bittner 2009; Dönz et al. 2018). The spawning season and depth of *C. brienzii* overlaps largely with that *C. fatioi*.

##### Etymology.

The specific epithet *brienzii* is the genitive of *Brienz*. We name this species after Lake Brienz, as it is the only endemic whitefish species known for Lake Brienz.

##### Common name.

None. We suggest the German name «Brienzer Kleinbalchen»

#### Coregonus profundus

Taxon classificationAnimaliaTeleosteiCoregonidae

Selz, Dönz, Vonlanthen & Seehausen sp. nov.

A7E464BB-B907-5E06-A9C4-E5BAA1944AC7

http://zoobank.org/6B17CFFD-08A3-4A6E-A4AA-CAE0678370FF

Coregonus alpinus : Kottelat 1997; Kottelat and Freyhof 2007; Hudson et al. 2011, 2013, 2016; Ingram et al. 2012; Vonlanthen et al. 2012, 2015; Dönz et al. 2018
Coregonus lavaretus natio *arurensis*, oekot. profundus: Steinmann 1950
Coregonus "Tiefenalbock", "Kropfer": Steinmann 1950
Coregonus "Kropfer": Heuscher 1901
Coregonus "Kropfer": Rufli 1978, 1979; Kirchhofer and Tschumi 1986; Kirchhofer 1995; Bittner et al. 2010 (see also synonymy of C. albellus)
Coregonus "Kropfer", "THU3": Douglas et al. 1999, 2003; Douglas and Brunner 2002

##### Material examined.

***Holotype*.** NMBE-1077208, Switzerland, Lake Thun (46°40'N, 7°46'E), 194 mm SL, male.

***Paratypes*.** NMBE-1077161–1077179, NMBE-1077203–1077207, NMBE-1077209–1077211, Switzerland, Lake Thun (46°40'N, 7°46'E), N = 27, 188–316 mm SL.

##### Diagnosis.

*Coregonus profundus* is a small whitefish species with moderate pigmentation of all fins and the body; brown-orange colouration on the flanks above the lateral line; elongate slender body; long head; large eye with a thick and triangular shaped eye socket; tip of snout is fleshy and roundish; few (15–27) and short gill rakers.

##### Differential diagnosis.

*Coregonus profundus* occurs only in Lake Thun and we therefore compare the characters of this species specifically with the species of Lake Thun. The differential diagnoses against *C. albellus*, *C. alpinus*, *C. fatioi*, and *C. steinmanni* are given under those species’ accounts. The lower number of gill rakers of *C. profundus* (total gill raker number: 15–27, mode = 21) distinguishes this species from all other 5 whitefish species, *C. albellus* (32–44, mode = 38), *C. alpinus* (25–34, mode = 30), *C. fatioi* (32–43, mode = 38), *C. steinmanni* (30–35, mode = 31), and *C. acrinasus* (30–40, mode = 36) (Suppl. material 1: Table S6).

***Coregonus profundus–Coregonus acrinasus***

*Coregonus profundus* can be distinguished from *C. acrinasus* by having shorter gill rakers (middle gill raker length: 7.6–11.7% HL, mean = 9.2 vs. 9.1–16.6% HL, mean = 13.4; longest gill raker length: 7.8–12.4% HL, mean = 10.1 vs. 11.4–16.9% HL, mean = 14.5) and a longer head (15.5–18.4% HL, mean = 16.4 vs. 13.8–16.1% HL, mean = 15.2) (Tables 8, 9).

##### Description.

**Figure 9.**
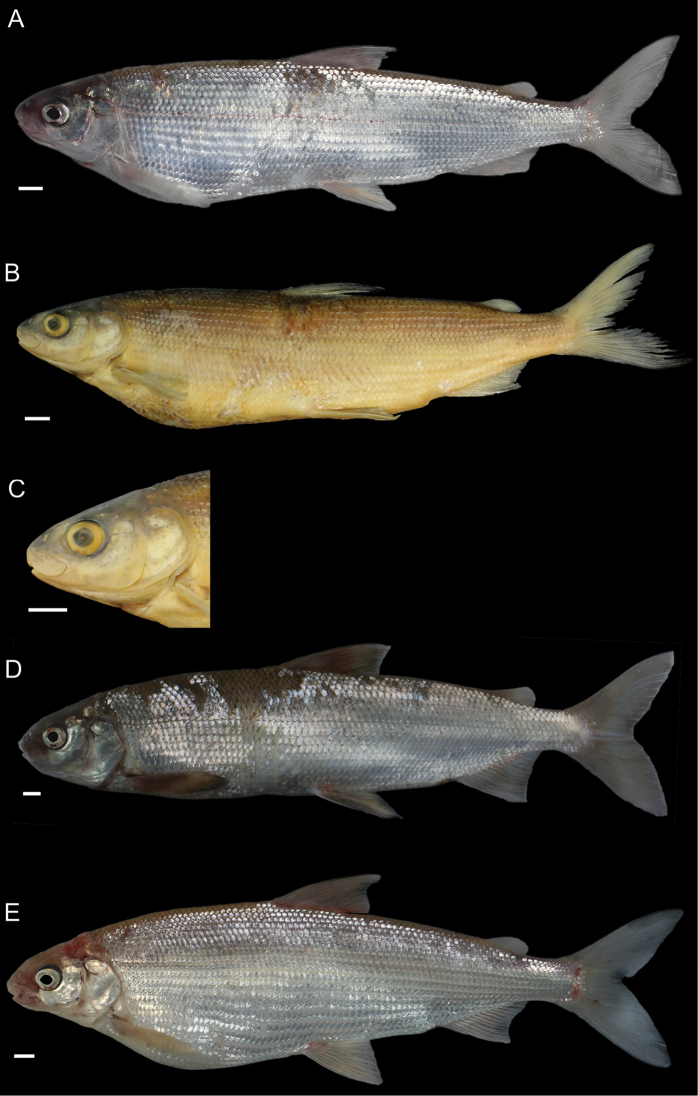
*
Coregonus profundus*, Lake Thun, Switzerland **A** holotype, NMBE-1077208, Lake Thun, 194 mm SL, male, freshly caught specimen **B, C** holotype, NMBE-1077208, preserved specimen **D** paratype, NMBE-1077203, Lake Thun, 315.5 mm SL, male **E** paratype, NMBE-1077166, Lake Thun, 253.5 mm SL, female. The white scale (1cm) below each fish acts as a reference for the actual size of the specimen.

General appearance is shown in Figure 9. Morphological and meristic characters of both sexes can be found in Table 8 and Suppl. material 1: Table S6 and first- and second-best ratios for both sexes combined can be found in Table 10. The description is valid for both sexes.

***Shape***: Body elongate. Slender bodied with greatest body depth anterior of the dorsal fin. Dorsal and ventral profile similar and slightly arched. Dorsal and ventral profile from tip of snout to interorbital area mostly straight and then slightly convex to dorsal and pelvic fin origin respectively. Head long. Snout often 60° angle to the body axis anterior of the eye, such that the profile from the tip of the snout to the vertical projection where the anterior part of the eye crosses the dorsal profile is straight and afterwards slightly convex. Mouth is wide (i.e., width of upper and lower jaw), rather short and mostly strongly sub-terminal and only rarely terminal. Snout is weakly pronounced, since the tip of the snout is often fleshy and roundish. Eye rather large with a large eye cavity and a thick and triangular eye-socket (i.e., sickle-shaped). Pectoral fin long and moderately tapered. Dorsal fin long with the anterior unbranched ray of the erected dorsal fin approx. 70–80° angle to body axis and only slightly bent posteriorly at the end of the ray. Caudal peduncle narrow and short with caudal fin forked and sometimes moderately to strongly asymmetrical with either the ventral or dorsal part being longer. Unbranched ray of anal fin straight and rarely bent posteriorly at the end of the ray. Anal fin is longest anteriorly and progressively shortening posteriorly with the outer margin of the anal fin slightly concave and only rarely straight.

***Meristics ***: Very few and very short gill rakers.

***Colour***: Pigmentation of fins and body is overall moderate in live specimens. Pectoral fin is translucent or yellowish in colouration with moderate pigmentation at the median to distal parts of the fin. Dorsal, adipose, pelvic, anal and caudal fins are moderately pigmented. Silvery appearance along the flanks and dorsally above the lateral line the silvery appearance changes to a pale brown-orange colouration (e.g., RGB (232, 172, 52)) and very rarely the brown-orange colouration can have a hint of light greenish colour (e.g., RGB (136, 245, 205)). Sometimes the colouration above the lateral line is pale rose (e.g., RGB (247, 187, 175)) and then towards the dorsum becomes a brown-orange. This transition from one colouration to another can also be observed in *C. albellus*. For a comparison to the main colouration found in the other species see Suppl. material 1: Figure S8. Dorsal part of the head is moderately pigmented. Snout around the nostrils is moderately pigmented and rarely with a gap of less pigmentation posteriorly of the nostrils up to the height of the middle of the eyes. The operculum and pre-operculum are silvery with one black dot on the lower margin of the pre-operculum. Preserved specimens are pale in colouration with similar pigmentation as described for live specimens. Silvery, translucent, not coloured or unpigmented parts of the body become brown-yellowish (e.g., RGB (239, 210, 40)), whereas the pigmented parts are conserved and the coloured parts (dorsally above the lateral line) become brownish (e.g., RGB (186, 140, 100)).

**Distribution and notes on biology.***Coregonus profundus* is found in Lake Thun (46°40'N, 7°46'E). It is believed to have been endemic to this lake. Yet, based on matching genetic (microsatellite) and morphological (gill raker number, morphological characters) evidence one ripe specimen of *C. profundus* has been caught by a local fisherman, Stefan Dasen, in 2016 in Lake Biel (47°05'N, 7°10'E) (Suppl. material 1: Figure S9). Lake Biel has been artificially connected with Lake Thun through the river Aare since the Jura water correction from 1868–1878, where the river Aare was artificially bypassed downstream from Lake Thun into Lake Biel. For another Lake Thun species, *C. albellus*, it had been known since at least 2004 that it can be found in Lake Biel (see details in the note on biology for *C. albellus*) (Bittner 2009; this study Suppl. material 1: Figure S9).

It is important to note that native whitefish species of Lake Biel were only known to spawn in the winter months (Fatio 1885; Steinmann 1950; Rufli 1978), whereas *C. profundus* as well as *C. albellus* spawn in late summer and winter. Our study reports the first record of *C. profundus* in Lake Biel. It is unclear though if *C. profundus* has established as a self-sustaining population in Lake Biel. So far, we only know of one ripe specimen of *C. profundus* from Lake Biel, whereas for *C. albellus* reasonable numbers of ripe specimen have been caught for several years in Lake Biel during what is the normal spawning period (late summer) of this species in lakes Thun and Brienz (Bittner 2009; 2016: Suppl. material 1: Figure S9). Based on isotopic signatures *C. profundus* feeds on benthic prey items (Selz 2008; Hudson 2011; Ingram et al. 2012) and has a slow growth rate (Bittner et al. unpublished). Interestingly specimens of *C. profundus* that have been caught on the spawning grounds of *C. albellus* were often in past-spawning condition and occasional stomach content analysis revealed that these fish had been heavily preying on whitefish eggs (Bittner 2009). Earlier stomach content analysis of *C. profundus* from the months of October and February of 1971 and 1972, respectively, showed that *C. profundus* mainly feed on chironomid larvae and occasionally on fish eggs (Rufli 1979). Even earlier stomach content analysis by Steinmann (1950) also show that they feed on chironomid larvae, but also on pisidium and other benthic invertebrates. Habitat-stratified random sampling of Lake Thun (mid-October 2013: Vonlanthen et al. 2015) shows that *C. profundus* occupies mostly the moderately deep to the deepest waters in the benthic habitat (approx. 15 – 210 m; *N* = 16) and rarely the moderately deep pelagic waters (approx. 15 – 45 m; *N* = 3)(Dönz et al. 2018). The habitat-stratified random sampling did not distinguish between ripe and unripe specimens, and thus in the case of *C. profundus*, the distribution pattern along the depth in the benthic zone is biased by the spawning aggregation of this species since the sampling period coincides partially with the spawning season of this species. *Coregonus profundus* phenotypically resembles superficially *C. albellus*. The average size (total length) at 3 years of age for specimens used in this study is 263±16 mm (mean and standard deviation, N = 11) (Suppl. material 1: Figures S4, S6). The size of 3-year-old specimens of *C. profundus* is similar to that of *C. albellus* and *C. fatioi*, but smaller than that of *C. acrinasus* and considerably smaller than that of *C. alpinus* and *C. steinmanni* (Suppl. material 1: Figure S6). *Coregonus profundus* has a moderately long spawning season from August to December with one major peak from late August to late September / early October (Suppl. material 1: Figure S3; Bittner 2009; Dönz et al. 2018). Spawning depth varies with spawning season and can range from approx. 30 m to 150 m (Suppl. material 1: Figure S3; Bittner 2009; Dönz et al. 2018). The spawning season and depth of *C. profundus* partially overlaps with that of *C. steinmanni*, *C. fatioi*, and *C. albellus* (Suppl. material 1: Figure S3; Bittner 2009; Dönz et al. 2018).

*Coregonus profundus* is known by the common name "Kropfer" and has previously been described under the name *C. alpinus* (Kottelat (1997) and Kottelat and Freyhof (2007)). As we explain in detail under the species account of *C. alpinus*, the designated lectotype of *C. alpinus* is incongruent with the description of the species (with the common name "Kropfer": Kottelat (1997) and Kottelat and Freyhof (2007)). We have thus retained the name *C. alpinus* for the lectotype designated by Kottelat (1997) and provided a new description of this taxon. For the species otherwise described by Kottelat (1997) and Kottelat and Freyhof (2007) as *C. alpinus* (with the common name "Kropfer") we designated a new name, *C. profundus*.

##### Etymology.

The adjective *profundus* means deep in Latin and is used for *C. profundus* to describe the species unique ecology of living and breeding in great depths in Lake Thun.

##### Common name.

Kropfer.

#### Coregonus acrinasus

Taxon classificationAnimaliaTeleosteiCoregonidae

Selz, Dönz, Vonlanthen & Seehausen sp. nov.

940B9032-9659-5B10-BEB9-6D28A49C2853

http://zoobank.org/FEB8CAC5-E55D-4A8C-8E21-94E4DB0E77B2

Coregonus "Albock": Kirchhofer 1995 (see also synonymy of C. alpinus and C. steinmanni)
Coregonus "Albock", "THU1": Douglas et al. 1999; Douglas and Brunner 2002; Douglas et al. 2003 (see also synonymy of C. fatioi)
Coregonus fatioi : Hudson et al. 2011, 2013, 2016; Ingram et al. 2012; Vonlanthen et al. 2012
Coregonus sp. "Albock": Doenz et al. 2018

##### Material examined.

***Holotype*.** NMBE-1077271, Switzerland, Lake Thun (46°40'N, 7°46'E), 239.5 mm SL, male.

***Paratypes*.** NMBE-1077238–1077240, NMBE-1077268–1077270, NMBE-1077272–1077290, Switzerland, Lake Thun (46°40'N, 7°46'E), N = 25, 197–278 mm SL.

##### Diagnosis.

*Coregonus acrinasus* is a medium-sized whitefish with moderate pigmentation of all fins and body; dark greenish blue colour on the flanks above the lateral line; moderate to many pigmented small dots on the scales; tip of the snout pointy; long head; small eye with a thick and triangular shaped eye socket; many and moderately long gill rakers.

##### Differential diagnosis.

*Coregonus acrinasus* only occurs in Lake Thun and shows ancestry contributions from whitefish of Lake Constance, besides its Lake Thun ancestry. These derive from historically documented introductions of at least two whitefish species (*C. wartmanni* and *C. macrophthalmus*) into Lake Thun. Since, historically undocumented introductions of other whitefish from Lake Constance cannot be excluded and since there is no clear genetic assignment of *C. wartmanni* or *C. macrophthalmus* as likely source of the allochthonous introgression we compare the characters of this species with those of all whitefish species from Lake Constance and all other whitefish species from Lake Thun. The differential diagnoses against *C. albellus*, *C. alpinus*, *C. fatioi*, *C. steinmanni* and *C. profundus* are given under those species’ accounts.

**Lake Constance comparison.**

***Coregonus acrinasus–all four Lake Constance species***

The wider underjaw of *C. acrinasus* (9.2–14.3% HL, mean = 12.2) differentiates it from all other species from Lake Constance, *C. gutturosus* (6.8–9.9% HL, mean = 7.7), *C. arenicolus* (7.8–8.5% HL, mean = 8.1), *C. macrophthalmus* (6.4–8.8% HL, mean = 8) and *C. wartmanni* (8.1% HL) (Tables 9, 12).

***Coregonus acrinasus–Coregonus wartmanni***

*Coregonus acrinasus* differs from *C. wartmanni* by having a larger eye and eye cavity (eye diameter: 21.6–25.5% HL, mean = 23.7 vs. 18.9% HL; eye cavity: 26–29.6% HL, mean = 27.7 vs. 23.9% HL; eye height: 21.7–24.8% HL, mean = 22.9 vs. 19% HL) (Tables 9, 12).

***Coregonus acrinasus–Coregonus macrophthalmus***

*Coregonus acrinasus* differs from *C. macrophthalmus* by having a wider head (43.9– 56.2% HL, mean = 49.6 vs. 39.3–43.3% HL, mean = 41.6) (Tables 9, 12).

***Coregonus acrinasus–Coregonus gutturosus***

*Coregonus acrinasus* differs from *C. gutturosus* by having more and longer gill rakers (upper arch gill raker number: 10–15, mode = 13 vs. 7–9, mode = 7; lower arch gill raker number: 20–26, mode = 24 vs. 9–12, mode = 10; total gill raker number: 30–40, mode = 36 vs. 16– 21, mode = 19; middle gill raker length: 9.1–16.6% HL, mean = 13.4 vs. 4.1–8.7% HL, mean = 6.9; longest gill raker length: 11.4– 16.9, mean = 14.5 vs. 6.7–10.6% HL, mean = 8.2), a longer lower jaw (38.6–47% HL, mean = 40.9 vs. 34.3–39.1% HL, mean = 36.6) and a shorter head (13.8–16.1% HL, mean = 15.2 vs. 15.4–18.1% HL, mean = 16.8) (Tables 9, 12, Suppl. material 1: Table S7).

***Coregonus acrinasus–Coregonus arenicolus***

*Coregonus acrinasus* can be differentiated from *C. arenicolus* by having more and longer gill rakers (lower arch gill raker number: 20–26, mode = 24 vs. 13–19; total gill raker number: 30–40, mode = 36 vs. 22–31; middle gill raker length: 9.1–16.6% HL, mean = 13.4 vs. 9.8– 10.6% HL, mean = 10.2; longest gill raker length: 11.4–16.9, mean = 14.5 vs. 10.9–12% HL, mean = 11.5), a larger eye (eye diameter: 21.6–25.5% HL, mean = 23.7 vs. 17.3–19.6% HL, mean = 17.7; eye cavity: 26–29.6% HL, mean = 27.7 vs. 24.1–25.7% HL, mean = 25; eye height: 21.7–24.8% HL, mean = 22.9 vs. 18.8–20.8% HL, mean = 19.6) and a shorter anal fin (9.2–13% HL, mean = 11.6 vs. 12.9–13.8 % HL, mean = 13.3) (Tables 9, 12, Suppl. material 1: Table S7).

##### Description.

**Figure 10.**
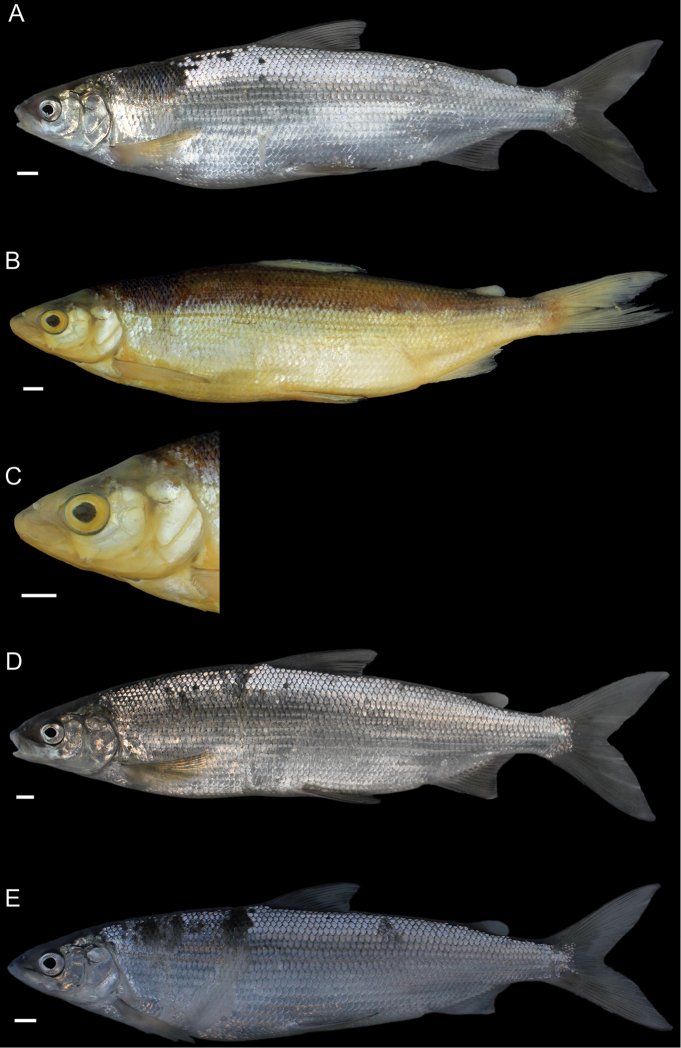
*
Coregonus acrinasus*, Lake Thun, Switzerland **A** holotype, NMBE-1077271, Lake Thun, 239.5 mm SL, male, freshly caught specimen **B, C** holotype, NMBE-1077271, preserved specimen **D** paratype, NMBE-1077270, Lake Thun, 270 mm SL, male, freshly caught specimen **E** paratype, NMBE-1077279, Lake Thun, 234 mm SL, male, freshly caught specimen. The white scale (1cm) below each fish acts as a reference for the actual size of the specimen.

General appearance is shown in Figure 10. Morphological and meristic characters of both sexes can be found in Tables 9, 12, and Suppl. material 1: Tables S6, S7 and first- and second-best ratios for both sexes combined can be found in Table 10. The description is valid for both sexes.

***Shape***: Only slightly deep bodied with greatest body depth anterior of the dorsal fin. Dorsal and ventral profile equally arched such that both the dorsal profile from the tip of snout to the anterior origin of dorsal fin and the ventral profile from the interorbital area to the pelvic fin origin are moderately convex. Head long. Mouth (i.e., width of upper and lower jaw) is thick, moderately long and often sub-terminal and only rarely terminal. Rostral plate is mostly wider than deep, not strongly pronounced and the tip of the snout is often pointy in the sagittal plane. Eye-socket thick and triangular (i.e., sickle-shaped). Pectoral fin moderately tapered. Anterior unbranched ray of the erected dorsal fin has an approx. 40–60° angle to body axis and from the middle to the end of the ray it is moderately bent posteriorly. Caudal peduncle stout and moderately long. Caudal fin forked and sometimes slightly asymmetrical with the ventral part being longer. Unbranched ray of anal fin mostly straight and only sometimes slightly bent posteriorly. Anal fin is longest anteriorly and progressively shortening posteriorly with the outer margin of the anal fin slightly concave.

***Meristics ***: Many and moderately long gill rakers.

***Colour***: Pigmentation of fins and body overall moderately strong in live specimens. Pectoral fin is mostly transparent to moderately pigmented with a yellowish faint pigmentation and only very rarely strongly pigmented. Dorsal, adipose, pelvic, anal, and caudal fins are moderately to strongly pigmented. Fish have a silvery appearance along the flanks with moderate to many pigmented small dots on the scales. Dots along the flank and the dorsum. Distribution of the dots is bound to the scale patterning such that the dots are found at the edge of the scales or at the boundary point of two scales. Dorsally above the lateral line the silvery appearance changes to dark greenish blue colour (e.g., RGB (7,168,125)). The snout around the nostrils is strongly pigmented with a gap of very little pigmentation posteriorly of the nostrils up to the height of the middle of the eyes. Pre-operculum and operculum are silvery with one black dot on the lower margin of the pre- operculum. For a comparison to the main colouration found in the other species see Suppl. material 1: Figure S8. Preserved specimens are pale in colouration with similar pigmentation as described for live specimens. Silvery, translucent, not coloured or unpigmented parts of the body become brown-yellowish (e.g., RGB (239, 210, 40)), whereas the pigmented parts are conserved and the coloured parts (dorsally above the lateral line) become brownish (e.g., RGB (186, 140, 100)).

##### Distribution and notes on biology.

*Coregonus acrinasus* is found in Lake Thun (46°40'N, 7°46'E). Based on isotopic signatures *C. acrinasus* most likely feeds on a mix of benthic prey and zooplankton (Selz 2008; Hudson 2011; Ingram et al. 2012) and based on the size-at-age data *C. acrinasus* must have a rather fast growth rate (Suppl. material 1: Figures S4–S6). The gill raker number and length of *C. acrinasus* (many gill rakers and moderately long gill rakers) suggests, based on the functional properties of the number of gill rakers on feeding on different prey items (Lundsgaard-Hansen et al. 2013; Roesch et al. 2013), that *C. acrinasus* feeds more on zooplankton and less on benthic prey, but this assumption needs to be verified in the future with stomach content analyses. The relative species abundances in the pelagic and benthic habitat from a habitat-stratified random sampling of Lake Thun (mid-October 2013: Vonlanthen et al. 2015) also points to this.*Coregonus acrinasus* occupies only the shallow waters of the benthic habitat (15 m; *N* = 1) and the moderately deep pelagic waters (approx. 10–35 m; *N* = 9) (Dönz et al. 2018). However, the habitat-stratified sampling needs to be treated with caution since it only shows a snapshot in time (one month) of the spatial distribution of this and the other species. *Coregonus acrinasus* phenotypically resembles to some extent *C. alpinus* and *C. steinmanni*. The average size (total length) at three years of age for specimens in this study of *C. acrinasus* is 304±21 mm (mean and standard deviation, N = 9) (Suppl. material 1: Figures S4, S6).The average size at 3 years of age for the specimens of *C. acrinasus* from this study is similar to that for the years 2004–2005 (322.8±18 mm, *N* = 50) (Bittner et al. unpublished; Vonlanthen et al. unpublished). The size of 3-year-old specimens of *C. acrinasus* is smaller to that of *C. alpinus* and *C. steinmanni* and considerably larger than that of *C. albellus*, *C. fatioi*, and *C. profundus* (Suppl. material 1: Figure S6). *Coregonus acrinasus* has a short spawning season in late December and very rarely have ripe individuals been caught in late autumn or winter (Suppl. material 1: Figure S3; Dönz et al. 2018). *Coregonus acrinasus* spawns mostly in moderately shallow waters of 10m down to approx. 100 m (Suppl. material 1: Figure S3; Bittner 2009; Dönz et al. 2018). The spawning season and depth of *C. acrinasus* overlaps largely with that of *C. alpinus*, *C. steinmanni*, and *C. fatioi* and to a much lesser extent with that of *C. albellus* and *C. profundus*.

*Coregonus acrinasus* appears to be a species of partially allochthonous origin, closely related to the radiation of Lake Constance with genetic contributions from Lake Thun. Indications of this situation were seen in several earlier genetic studies (Douglas and Brunner 2002; Douglas et al. 2003; Bittner 2009; Hudson et al. 2011) and this was clearly confirmed with large sample sizes recently (Hudson et al. 2016; Dönz et al. 2018). Historical records mention the stocking of alevins of the Lake Constance endemics *C. wartmanni* and *C. macrophthalmus* into Lake Thun. To fully understand the relationship of *C. acrinasus* to the Lake Constance species, we compared the morphology of *C. acrinasus* with that of all four described species of Lake Constance, *C. wartmanni*, *C. macrophthalmus*, *C. arenicolus*, and the now extinct *C. gutturosus*. Our data clearly reveal *C. acrinasus* as distinct from all Lake Constance species based on morphological characters. Historical records from Fatio (1890) in his book on Swiss fish (Fatio 1890: Page 123) and from Heuscher (1901) in his report on the biology of lakes Thun and Brienz (Heuscher 1901: Pages 69–70, 103) report several incidences of introductions of whitefish from other lakes. Evidence for additional introductions comes from historical records from a fisheries club that was responsible for the propagation of whitefish in lakes Thun and Brienz before stocking with allochthonous fish was forbidden in Lake Thun (nothing is stated regarding Lake Brienz) in 1946 by the local fisheries authorities (Douglas et al. 2003; Dönz et al. 2018). Since 1991 such introductions were banned in all of Switzerland through federal law (BGF 6 I b). These historical records reveal that in 1888, 1889, and 1934 in Lake Thun and 1892 in Lake Brienz between 20’000 and 750’000 (Lake Thun) and once 39’000 (Lake Brienz) fry of either *C. macrophthalmus* (only Lake Thun) or *C. wartmanni* (both lakes) were stocked. Heuscher (1901: Page 70) further noted that the introductions of 1888, 1889, and 1892 were unsuccessful in both lakes, as fishermen did not catch adult fish of either of the Lake Constance species ever after those introductions. Steinmann (1950) in his monograph on Swiss whitefish diversity did not mention any species from Lake Constance to be present in Lake Thun or in Lake Brienz. Dönz et al. (2018) could recently show with genetic data from scales dating back to 1972 and earlier that *C. acrinasus* was completely absent in catches of that period. The first qualitative reports of this species in spawning fisheries catches are from around the year 2000 (Douglas et al. 1999; Bittner 2009), and our own genetic data from samples of more than 2000 whitefish from Lake Thun confirm the presence of the species. Based on a recent lake-wide quantitative survey in 2015 Dönz et al. (2018) showed that this species accounts for ca. 10% of all whitefish in Lake Thun in abundance when based on genetic assignments. Several independent multilocus microsatellite and AFLP data sets suggest that it has genetic contributions from the endemic Lake Thun species and cannot clearly be designated genetically to one of the Lake Constance species (Douglas and Brunner 2002; Douglas et al. 2003; Bittner 2009; Hudson et al. 2011; Hudson et al. 2016; Dönz et al. 2018). This suggests that some individuals of one or several of the introduced species from Lake Constance must have successfully reproduced in Lake Thun and hybridized with one or several of the local species.

##### Etymology.

The name *C. acrinasus* is a combination of the ablative case of the Latin adjective *acer* resulting in *acri*, which means pointed and the noun *nasus* for nose. The name acrinasus refers to a phenotypic feature of this species, which often has a pointed snout when viewed in the sagittal plane.

##### Common name.

Albock

### Lake Constance whitefish species

#### Coregonus gutturosus

Taxon classificationAnimaliaTeleosteiCoregonidae

, Gmelin, 1818

0BB524ED-7A60-5FB7-B6EA-87ED1E760996

##### Material examined.

***Non-types*.** NMBE-1076230 (Eawag-246), NMBE-1076232 (Eawag-248–1), NMBE-1076233 (N = 6: Eawag–249–1, Eawag-249–2, Eawag-249–3, Eawag-249–4, Eawag-249–5, Eawag-249–6), NMBE-1076232 (N = 2: Eawag-248–2, Eawag-248–3), Switzerland, Lake Constance (47°38'N, 9°22'E), N = 10, 169–292 mm SL.

##### Distribution and notes on biology.

*Coregonus gutturosus* used to be endemic to Lake Constance but is now extinct.

##### Common name.

Kilch

#### Coregonus arenicolus

Taxon classificationAnimaliaTeleosteiCoregonidae

, Kottelat, 1997

CAA82ADB-570A-52F7-A3E7-E67BED400C99

##### Material examined.

***Holotype*.** NMBE-1076223 (Eawag-239–1), Switzerland, Lake Constance (47°38'N, 9°22'E), 296 mm SL, sex unknown.

***Paratypes*.** NMBE-1076223 (N = 3: Eawag-239–2,Eawag-239–3, Eawag-239–4), Switzerland, Lake Constance (47°38'N, 9°22'E), N = 3, 289–314 mm SL.

##### Distribution and notes on biology.

*Coregonus arenicolus* is found in the upper and lower basin of Lake Constance.

##### Common name.

Sandfelchen.

#### Coregonus macrophthalmus

Taxon classificationAnimaliaTeleosteiCoregonidae

, Nüsslin, 1882

823F455D-4840-5222-8CAF-40036010FB36

##### Material examined.

***Syntypes*.** MHNG-716.052, MHNG-716.051, MHNG-816.02, MHNG-715.094 (N = 2: MHNG-715.094–1, MHNG-715.094–2), NMBE-1076211 (N = 2: Eawag-227–1, Eawag-227–2), Switzerland, Lake Constance (47°38'N, 9°22'E), N = 7, 193–235 mm SL.

##### Distribution and notes on biology.

*Coregonus macrophthalmus* is found in Lake Constance, especially in the upper basin (Obersee). It is unclear if it also occurs in the lower basin (Untersee) of the lake.

##### Common name.

Gangfisch.

#### Coregonus wartmanni

Taxon classificationAnimaliaTeleosteiCoregonidae

, Bloch, 1784

A1DF7123-9A65-5806-996D-041B1ED8CA8E

##### Material examined.

***Non-type*.** NMBE-1076206, Switzerland, Lake Constance (47°38'N, 9°22'E), 301 mm SL, sex female.

##### Distribution and notes on biology.

*Coregonus wartmanni* is found in Lake Constance, especially in the upper basin (Obersee). It is unclear if it also occurs in the lower basin (Untersee).

##### Common name.

Blaufelchen.

### Identification key to the species of lakes Thun and Brienz

**Lake Thun**

**Table:** 

1	Caudal peduncle depth / upper jaw length ratio is 1.36–1.65 and caudal peduncle depth / maxilla length ratio is 1.77–2.24	**2**
–	Caudal peduncle depth / upper jaw length ratio is 0.96–1.43	**3**
2	Total number of gill rakers 25–30	*** C. alpinus***
–	Total number of gill rakers 31–35	*** C. steinmanni***
3	Total number of gill rakers 15–27	*** C. profundus***
–	Total number of gill rakers 30–44	**4**
4	Colouration above the lateral line on the dorsum from a pale rose colouration to a pale brown colouration; no or few small pigmented dots on the edge of the scales or on the boundary of two scales on the flank; no pigmented dots on the dorsum	*** C. albellus***
–	Colouration above the lateral line on the dorsum from a light to dark green and rarely a light olive; moderate to many dots on the edge of the scales or on the boundary of two scales on the flank and/or the dorsum	**5**
5	Angle to body axis of the erected dorsal fin approx. 60–80°	*** C. fatioi***
–	Angle to body axis of the erected dorsal fin approx. 40–60°	*** C. acrinasus***

**Lake Brienz**

**Table:** 

1	Total number of gill rakers 26–30 and erected dorsal fin length / upper jaw length ratio is 3.25–4.1	*** C. alpinus***
–	Total number of gill rakers 32–42 and erected dorsal fin length / upper jaw length ratio is 2.14–3.19	**2**
2	Predorsal length / eye height ratio is 6.1–7.58	*** C. albellus***
–	Predorsal length / eye height ratio is 8.12–10.5	**3**
3	Body depth 19.6–25.1% SL, eye depth 23.1–28.3 % HL	*** C. brienzii***
–	Body depth 22.1–26.2% SL, eye depth 21.2–27.6% HL	*** C. fatioi***

**Table 3.** Morphological and meristic data of *C. albellus* Fatio, 1890 from lakes Thun and Brienz, MHNG-816.022 lectotype from Lake Thun; non-type material N = 34 from Lake Thun and N = 32 from Lake Brienz.

**Morphological/ characters**	*** C. albellus***	**Both lakes**		**Lake Thun**						**Lake Brienz**					
	**Lectotype**	**Non-types both sexes**		**Non-types**						**Non-types**					
		***N-total* = 66**		***N-total* = 34**		***N-females* = 21**		***N-males* = 13**		***N-total* = 32**		***N-females* = 19**		***N-males* = 13**	
		**Mean ± StDev**	**Range**	**Mean ± StDev**	**Range**	**Mean ± StDev**	**Range**	**Mean ± StDev**	**Range**	**Mean ± StDev**	**Range**	**Mean ± StDev**	**Range**	**Mean ± StDev**	**Range**
**SL (mm)**	165.0	173.2±51.9	(101–271)	221.4±16.4	(177–271)	224.4±14.9	(205–271)	216.6±18.1	(177–254)	122.1±11.0	(101–164)	122.9±13.5	(101–164)	120.9±6.4	(108–129)
**Percentage of standard length**															
**PelvFB**	3.6	3.9±0.4	(3.2–5.6)	4.0±0.3	(3.4–4.7)	4.0±0.3	(3.4–4.7)	4.1±0.2	(3.7–4.5)	3.8±0.5	(3.2–5.6)	4.0±0.5	(3.2–5.6)	3.6±0.2	(3.3–4.0)
**PelvFS**	na	6.3±0.7	(5.1–8.6)	6.2±0.7	(5.1–7.3)	6.1±0.7	(5.1–7.2)	6.5±0.7	(5.1–7.3)	6.4±0.7	(5.5–8.6)	6.5±0.8	(5.5–8.6)	6.3±0.6	(5.5–7.8)
**PelvF**	14.1	17±1.1	(14.7–20.9)	16.7±0.9	(14.8–18.7)	16.5±0.6	(15.2–18)	17±1.1	(14.8–18.7)	17.3±1.1	(14.7–20.9)	17.5±1.4	(14.7–20.9)	17.1±0.7	(15.3–18.1)
**PecFB**	3.1	3.3±0.3	(2.7–4.8)	3.4±0.3	(2.7–3.9)	3.3±0.3	(2.7–3.8)	3.5±0.2	(2.9–3.9)	3.3±0.4	(2.9–4.8)	3.5±0.5	(3.0–4.8)	3.2±0.2	(2.9–3.6)
**PecF1**	na	17.6±1.2	(14.7–22.6)	17.3±1.0	(14.7–19.1)	17.2±0.9	(15.4–19.1)	17.4±1.1	(14.7–18.8)	18.0±1.4	(15.7–22.6)	18.2±1.5	(15.7–22.6)	17.6±1.3	(15.9–19.8)
**PecF2**	na	18.9±1.3	(15.7–23.8)	18.4±1.1	(15.7–20.7)	18.2±1	(16.4–20.1)	18.8±1.3	(15.7–20.7)	19.4±1.3	(16.9–23.8)	19.5±1.5	(16.9–23.8)	19.3±1	(18.1–21.2)
**DFB**	11.7	12.1±0.9	(10.7–16.2)	11.9±0.6	(10.8–13.6)	11.8±0.6	(10.8–13.3)	12.0±0.7	(11.0–13.6)	12.2±1.1	(10.7–16.2)	12.6±1.2	(10.8–16.2)	11.8±0.9	(10.7–13.2)
**DFAe**	na	19.1±1.4	(16.1–24.7)	18.5±1.2	(16.1–21.2)	18.3±0.9	(16.1–19.6)	18.9±1.5	(16.7–21.2)	19.7±1.5	(17.3–24.7)	20.2±1.6	(17.8–24.7)	19.1±0.9	(17.3–20.8)
**DFAd**	na	20.1±1.4	(17.1–26.6)	19.7±1.1	(17.1–21.9)	19.5±0.9	(17.3–21.4)	20.0±1.3	(17.1–21.9)	20.6±1.5	(18.3–26.6)	21.0±1.7	(18.3–26.6)	20.0±0.9	(18.9–21.7)
**DFPe**	7	5.4±0.7	(3.7–7.2)	5.1±0.6	(3.7–6.1)	5.0±0.5	(3.7–5.9)	5.2±0.7	(3.8–6.1)	5.7±0.7	(4.4–7.2)	5.7±0.7	(4.4–7.2)	5.7±0.6	(4.4–7.2)
**AFB**	13.1	12.9±1	(10.5–17)	12.7±0.8	(11.1–14.9)	12.4±0.6	(11.1–13.3)	13.1±0.8	(12.0–14.9)	13.0±1.2	(10.5–17.0)	13.3±1.3	(11.5–17.0)	12.7±1.0	(10.5–14.9)
**AFAe**	na	12±0.9	(9.7–14.4)	12.3±0.8	(10.9–14.0)	12.0±0.7	(10.9–13.4)	12.7±0.9	(11.3–14.0)	11.7±1.0	(9.7–14.4)	11.8±1.0	(9.7–14.4)	11.6±1.0	(9.7–13.8)
**AdFB**	6.8	6.5±1.1	(4.5–9.2)	5.8±0.7	(4.5–7.7)	5.8±0.7	(4.7–6.8)	5.9±0.8	(4.5–7.7)	7.2±1.1	(4.5–9.2)	7.2±1.1	(4.9–9.2)	7.1±1.1	(4.5–8.6)
**CF**	na	25±1.8	(21.7–32.9)	24±1.3	(21.7–26.5)	23.8±1.1	(21.7–26.4)	24.2±1.5	(21.8–26.5)	26±1.8	(24–32.9)	26.5±1.9	(24.4–32.9)	25.3±1.4	(24–28.6)
**CD**	6.9	7.2±0.5	(6.3–10)	7.1±0.3	(6.4–7.9)	7.1±0.4	(6.4–7.9)	7.2±0.3	(6.8–7.7)	7.3±0.6	(6.3–10)	7.5±0.8	(6.6–10)	7±0.3	(6.3–7.4)
**CL**	14.7	13.3±0.8	(11.7–16)	13.2±0.7	(11.7–14.6)	13.2±0.7	(11.9–14.5)	13.1±0.9	(11.7–14.6)	13.5±0.9	(12.1–16.0)	13.4±0.9	(12.2–16.0)	13.6±0.9	(12.1–15.5)
**PAdC**	18.9	19.8±1.5	(16.9–24.2)	18.9±1	(16.9–20.9)	18.8±0.9	(16.9–20.9)	19.3±0.9	(17.3–20.4)	20.7±1.4	(17.7–24.2)	20.5±1.8	(17.7–24.2)	20.9±0.6	(19.6–21.8)
**DHL**	14.7	16.4±1.8	(14.1–23.1)	15.0±0.7	(14.1–16.5)	14.9±0.7	(14.1–16.2)	15.2±0.7	(14.1–16.5)	17.9±1.3	(16.1–23.1)	18.3±1.4	(16.8–23.1)	17.3±0.9	(16.1–19.7)
**PreP**	49.2	51.9±2.4	(48.4–67.7)	52.0±1.6	(49.5–56.0)	52.1±1.7	(49.5–56.0)	51.9±1.4	(49.5–54.3)	51.8±3.2	(48.4–67.7)	52.2±3.9	(49.1–67.7)	51.4±1.7	(48.4–54.0)
**PreA**	76.2	77.1±3.1	(74–100)	77.1±1.3	(74.8–80.0)	77.2±1.2	(75.2–79.7)	76.9±1.6	(74.8–80.0)	77.2±4.3	(74.0–100.0)	77.7±5.5	(74.8–100.0)	76.4±1.4	(74.0–78.7)
**PreD**	45.5	47.2±2.2	(43.1–61.2)	46.5±1	(43.1–48.4)	46.6±0.9	(44.9–48.4)	46.2±1.1	(43.1–47.7)	48±2.7	(45.8–61.2)	48.5±3.3	(45.8–61.2)	47.3±1.3	(46–51.3)
**BD**	25.2	23.4±2.8	(16.9–30.2)	25.3±1.4	(23.0–29.6)	25.5±1.5	(23.5–29.6)	24.9±1.2	(23.0–26.8)	21.4±2.5	(16.9–30.2)	22.2±2.8	(19.4–30.2)	20.2±1.4	(16.9–22.6)
**PostD**	43.8	44.3±2	(41.2–56.2)	44.5±1.5	(41.8–48.3)	44.6±1.4	(41.8–46.6)	44.3±1.7	(43–48.3)	44.1±2.5	(41.2–56.2)	44.1±3.1	(41.5–56.2)	44±1.3	(41.2–45.8)
**TL**	na	121.3±2.1	(116.6–130)	120.7±1.8	(116.6–123.7)	120.5±1.8	(116.6–123.7)	121.1±1.8	(118.4–123.7)	122±2.2	(118.2–130)	122.1±2.3	(119.6–130)	121.7±2.2	(118.2–125.5)
**HL (mm)**	34.4	38.4±8.8	(26–51.9)	46.4±3.2	(36.1–51.9)	46.6±2.8	(41.5–51.9)	46.1±3.8	(36.1–51.6)	29.9±2.5	(26–39.2)	30.6±3	(26–39.2)	28.8±1	(27.7–31.1)
**Percentage of head length**															
**SN**	25.4	22.8±2	(18.7–27.2)	24.2±1.3	(20.9–27.2)	24.1±1.3	(20.9–26.3)	24.4±1.3	(22.5–27.2)	21.2±1.4	(18.7–25.2)	21.3±1.2	(19.7–23)	21.1±1.6	(18.7–25.2)
**ED**	23.5	26.2±3.3	(21.4–32)	23.4±1.3	(21.4–25.9)	23.7±1.2	(21.7–25.9)	22.9±1.3	(21.4–25.3)	29.3±1.5	(26.1–32)	29.4±1.7	(26.1–32)	29.2±1.2	(27.4–31.2)
**Morphological/ characters**	*** C. albellus***	**Both lakes**		**Lake Thun**						**Lake Brienz**					
	**Lectotype**	**Non-types both sexes**		**Non-types**						**Non-types**					
		***N-total* = 66**		***N-total* = 34**		***N-females* = 21**		***N-males* = 13**		***N-total* = 32**		***N-females* = 19**		***N-males* = 13**	
		**Mean ± StDev**	**Range**	**Mean ± StDev**	**Range**	**Mean ± StDev**	**Range**	**Mean ± StDev**	**Range**	**Mean ± StDev**	**Range**	**Mean ± StDev**	**Range**	**Mean ± StDev**	**Range**
**EC**	27	29.9±3.7	(23.1–36.8)	26.6±1.4	(23.1–29.2)	26.9±1.4	(24–29.2)	26.2±1.4	(23.1–28.1)	33.3±1.6	(30.4–36.8)	33.2±1.9	(30.4–36.8)	33.5±1.2	(31.5–36.2)
**EH**	na	26.1±2.9	(20.8–30.6)	23.5±1.3	(20.8–26.2)	23.7±1.4	(20.8–26.2)	23.4±1	(21.4–25.1)	28.7±1.1	(26.5–30.6)	28.7±1.2	(26.5–30.6)	28.9±0.9	(27.1–30.4)
**ES**	na	3.4±0.7	(2–4.9)	3.2±0.8	(2.0–4.9)	3.1±0.8	(2.0–4.5)	3.3±1.0	(2.1–4.9)	3.5±0.5	(2.5–4.8)	3.5±0.5	(2.9–4.8)	3.5±0.5	(2.5–4.3)
**PostO**	49.8	50.2±2.5	(45.5–55.6)	52±1.5	(48.5–55.6)	51.8±1.6	(48.5–55.6)	52.3±1.4	(50.1–55.1)	48.2±1.8	(45.5–53.1)	48.3±2	(45.5–53.1)	48.2±1.4	(45.7–50.5)
**HD**	75.5	68.3±4.5	(59.2–80.3)	71.8±2.9	(66.9–80.3)	71.5±3.2	(66.9–80.3)	72.2±2.4	(67.1–75.8)	64.6±2.3	(59.2–69)	64.4±2.4	(59.2–68.8)	64.8±2.3	(61.3–69)
**MW**	na	10.4±0.8	(8.8–12.2)	10.3±0.7	(8.8–12)	10±0.6	(8.8–11.2)	10.8±0.7	(9.4–12)	10.5±0.8	(8.8–12.2)	10.5±0.8	(8.9–12.2)	10.5±0.8	(8.8–11.7)
**UJ**	na	31.7±1.5	(28.6–34.9)	31.2±1.6	(28.8–34.7)	30.9±1.5	(28.8–33.9)	31.8±1.7	(29–34.7)	32.1±1.3	(28.6–34.9)	31.8±1.1	(28.6–33.4)	32.6±1.3	(30.3–34.9)
**LJ**	45.5	43.6±2.8	(38.4–49.2)	41.4±1.6	(38.4–44.6)	41.2±1.7	(38.6–44.4)	41.6±1.6	(38.4–44.6)	45.9±1.6	(41.9–49.2)	45.6±1.7	(41.9–48.5)	46.4±1.5	(43.9–49.2)
**M**	na	23.5±1.7	(20.1–26.9)	22.4±1.4	(20.1–26.8)	22.1±1.4	(20.1–26.8)	22.8±1.3	(21.5–26.4)	24.7±1.1	(22.6–26.9)	24.4±1.1	(22.6–26.5)	25.1±1.2	(23.4–26.9)
**SD**	na	8.8±1.6	(5.6–13.1)	9.6±1.6	(6–13.1)	9.2±1.4	(6–12.7)	10.3±1.7	(7.4–13.1)	8±1	(5.6–10.4)	8.2±1.1	(5.6–10.4)	7.7±0.6	(6.4–8.6)
**SW**	na	17.9±1.5	(13.5–21.1)	17.4±1.7	(13.5–20)	16.9±1.5	(13.5–19.1)	18.1±1.6	(14.3–20)	18.4±1.2	(15.4–21.1)	18.4±1.3	(15.4–20.6)	18.4±1.2	(16.3–21.1)
**HW**	48.1	48.5±4.3	(37.5–56.1)	50.7±3.3	(43.5–56.1)	50.6±3.2	(45.1–56.1)	50.7±3.6	(43.5–55.1)	46.2±4.1	(37.5–54.7)	45.7±3.7	(37.5–51.7)	46.9±4.5	(41.3–54.7)
**IOW**	25.8	26.9±2.2	(22.7–33.6)	28.4±2	(24.4–33.6)	28.4±2	(24.8–33.6)	28.4±2.1	(24.4–31.9)	25.4±1.2	(22.7–27.5)	25.4±1.3	(23.1–27.5)	25.5±1.2	(22.7–27.4)
**INW**	na	11.5±1.3	(7.8–15.4)	11.9±1.3	(9.9–15.4)	11.8±1.4	(9.9–15.4)	12.1±1.3	(9.9–15)	11±1.2	(7.8–13.7)	11.1±1.1	(9–13.7)	11±1.3	(7.8–13)
**LJW**	12.2	11.7±2.4	(7.5–17.5)	13.3±2.2	(8.6–17.5)	13.1±2	(9–16.5)	13.5±2.4	(8.6–17.5)	10±1.3	(7.5–12.3)	9.9±1.2	(8.4–12.3)	10±1.5	(7.5–12.2)
**UJW**	na	24.5±2.2	(19.1–28.9)	25.4±1.9	(20.9–28.7)	24.9±1.9	(20.9–28.7)	26.2±1.8	(22.7–28.4)	23.5±2	(19.1–28.9)	23.4±2.1	(19.1–28.9)	23.6±1.9	(19.8–27.4)
**MGR**	16.1	16±1.5	(11.7–19.4)	15.6±1.7	(11.7–18.3)	15.7±1.6	(11.7–18.3)	15.5±1.8	(12–18.2)	16.5±1.2	(13.7–19.4)	16.4±1.3	(13.7–19.3)	16.6±1.2	(15.1–19.4)
**LGR**	17	17.7±1.6	(14.1–21.8)	17.2±1.5	(14.1–20.3)	17.3±1.6	(14.1–20.3)	17±1.4	(14.7–18.9)	18.2±1.5	(14.9–21.8)	18±1.6	(14.9–21.8)	18.6±1.2	(16.8–21.5)
**UA**	20	19.5±1.4	(14.8–22.6)	19.2±1.4	(14.8–22.6)	19.1±1.6	(14.8–22.6)	19.5±1.2	(17.5–21.3)	19.7±1.4	(17.2–22.1)	19.5±1.5	(17.2–22.1)	20±1.1	(17.8–21.3)
**LA**	37.8	36.4±2.7	(29.6–42.2)	35.4±1.7	(32.3–39.6)	35.4±1.9	(32.3–39.6)	35.4±1.4	(32.8–37.5)	37.5±3.1	(29.6–42.2)	36.8±3.6	(29.6–42.2)	38.7±1.9	(35.3–42)
**Meristic characters**		**Mode**	**Range**	**Mode**	**Range**	**Mode**	**Range**	**Mode**	**Range**	**Mode**	**Range**	**Mode**	**Range**	**Mode**	**Range**
**PelvF unbranched**	1	1	(1–1)	1	(1–1)	1	(1–1)	1	(1–1)	1	(1–1)	1	(1–1)	1	(1–1)
**PelvF branched**	11	10	(9–12)	10	(9–11)	10	(9–11)	10	(9–11)	10	(10–12)	10	(10–11)	10	(10–12)
**PecF unbranched**	1	1	(1–1)	1	(1–1)	1	(1–1)	1	(1–1)	1	(1–1)	1	(1–1)	1	(1–1)
**PecF branched**	16	16	(14–17)	16	(14–17)	16	(14–17)	16	(15–17)	16	(15–17)	16	(15–17)	16	(15–17)
**DF unbranched**	3	4	(3–4)	4	(3–4)	4	(3–4)	4	(3–4)	4	(3–4)	4	(3–4)	4	(3–4)
**DF branched**	11	10	(9–12)	10	(9–12)	10	(9–12)	10	(9–12)	10	(9–12)	10	(9–12)	10	(9–11)
**Meristic characters**	*** C. albellus***	**Both lakes**		**Lake Thun**						**Lake Brienz**					
	**Lectotype**	**Non-types both sexes**		**Non-types**						**Non-types**					
		***N-total* = 66**		***N-total* = 34**		***N-females* = 21**		***N-males* = 13**		***N-total* = 32**		***N-females* = 19**		***N-males* = 13**	
		**Mode**	**Range**	**Mode**	**Range**	**Mode**	**Range**	**Mode**	**Range**	**Mode**	**Range**	**Mode**	**Range**	**Mode**	**Range**
**AF unbranched**	3	3	(2–4)	3	(2–4)	3	(2–4)	3	(2–4)	4	(3–4)	4	(3–4)	4	(3–4)
**AF branched**	14	12	(10–14)	12	(10–13)	12	(10–13)	12	(11–13)	12	(10–14)	12	(11–14)	12	(10–14)
**LS**	75	79	(70–86)	79	(73–86)	79	(73–83)	77	(76–86)	76	(70–84)	77	(70–80)	78	(70–84)
**PDS**	na	31	(26–37)	34	(29–37)	34	(29–37)	34	(30–37)	31	(26–35)	32	(30–35)	31	(26–34)
**TDS**	9	9	(7–10)	9	(8–10)	9	(8–10)	9	(9–10)	8	(7–9)	8	(7–9)	8	(7–9)
**TAS**	7	8	(6–9)	8	(7–9)	8	(7–9)	8	(8–9)	7	(6–8)	7	(6–8)	8	(6–8)
**TPS**	8	9	(7–9)	9	(8–9)	9	(8–9)	9	(8–9)	8	(7–9)	8	(7–9)	8	(7–8)
**UGR**	11	13	(9–17)	13	(9–17)	13	(9–15)	14	(11–17)	13	(11–16)	13	(11–16)	13	(12–14)
**LGR**	23	25	(20–29)	24	(20–28)	24	(20–28)	26	(22–27)	25	(22–29)	25	(22–29)	27	(24–28)
**total GR**	34	38	(32–44)	38	(32–44)	37	(32–42)	38	(33–44)	40	(35–42)	38	(35–42)	40	(37–42)

**Table 4.** Morphological and meristic data of *C. alpinus*Fatio 1885 from lakes Thun and Brienz, MHNG-717.045 lectotype from Lake Thun; non-type material N = 21 from Lake Thun and N = 9 from Lake Brienz.

**Morphological characters**	*** C. alpinus***	**Both lakes**		**Lake Thun**						**Lake Brienz**					
	**Lectotype**	**Non-types both sexes**		**Non-types**						**Non-types**					
		***N-total* = 30**		***N-total* = 21**		***N-females* = 12**		***N-males* = 9**		***N-total* = 9**		***N-females* = 6**		***N-males* = 3**	
		**Mean ± StDev**	**Range**	**Mean ± StDev**	**Range**	**Mean ± StDev**	**Range**	**Mean ± StDev**	**Range**	**Mean ± StDev**	**Range**	**Mean ± StDev**	**Range**	**Mean ± StDev**	**Range**
**SL (mm)**	283.0	266.1±56.4	(147-364)	288.3±45.1	(210-364)	299.6±37.3	(267-364)	273.3±52.2	(210-352)	214.3±46.0	(147-290)	240.1±27.9	(213-290)	162.7±23.3	(147-190)
**Percentage of standard length**															
**PelvFB**	4.5	4.2±0.4	(3.1-5.0)	4.4±0.3	(3.8-5)	4.3±0.4	(3.8-5.0)	4.4±0.3	(4.0-4.8)	3.7±0.3	(3.1-4.0)	3.9±0.1	(3.7-4.0)	3.5±0.5	(3.1-4.0)
**PelvFS**	4.7	6.1±0.8	(4.7-7.6)	5.9±0.7	(4.7-7.2)	5.9±0.7	(4.8-6.6)	5.9±0.8	(4.7-7.2)	6.7±0.5	(5.9-7.6)	6.8±0.5	(6.0-7.6)	6.5±0.6	(5.9-7.1)
**PelvF**	18.1	16.7±1.1	(14-18.2)	16.6±0.9	(14.7-18.2)	16.2±0.9	(14.7-17.3)	17.2±0.6	(16.3-18.2)	16.9±1.5	(14-18.1)	16.9±1.6	(14-18.1)	16.8±1.4	(15.2-18)
**PecFB**	3.7	3.3±0.3	(2.5-3.7)	3.4±0.3	(2.8-3.7)	3.4±0.3	(2.8-3.7)	3.3±0.2	(3.0-3.5)	3.0±0.3	(2.5-3.5)	3.0±0.3	(2.5-3.5)	3.1±0.3	(2.8-3.3)
**PecF1**	18.7	16.2±1.2	(13.6-18.7)	16.2±1.3	(13.6-18.7)	16.0±1.4	(13.6-18.6)	16.5±1.1	(15.1-18.7)	16.3±1.1	(13.9-17.1)	16.4±1.3	(13.9-17.1)	16.1±0.9	(15.1-16.8)
**PecF2**	19.7	17±1.1	(14.4-19.7)	17±1.1	(15.3-19.7)	16.8±1.1	(15.3-19.1)	17.3±1.1	(15.8-19.7)	16.9±1.2	(14.4-17.7)	17±1.3	(14.4-17.7)	16.7±1.1	(15.5-17.6)
**DFB**	12.9	12.8±0.9	(10.7-14.7)	12.9±0.9	(11.5-14.7)	12.9±0.9	(11.7-14.4)	12.9±0.9	(11.5-14.7)	12.6±1.1	(10.7-14.3)	12.6±1.1	(10.7-14.0)	12.7±1.4	(11.7-14.3)
**DFAe**	na	19.7±1.2	(16.4-23.0)	19.5±0.8	(17.6-20.6)	19.3±0.8	(17.6-20.5)	19.7±0.8	(18.6-20.6)	20.3±1.8	(16.4-23.0)	20.1±1.9	(16.4-21.5)	20.9±1.9	(19.5-23.0)
**DFAd**	22.6	20.8±1.4	(17.0-24.0)	20.6±0.9	(18.6-22.5)	20.4±0.8	(18.6-21.3)	20.9±1.1	(19.6-22.5)	21.4±2.0	(17.0-24.0)	21.2±2.2	(17.0-22.7)	21.7±2.0	(20.0-24.0)
**DFPe**	5.6	5.0±0.6	(4.0-6.5)	4.8±0.5	(4-5.8)	4.8±0.6	(4.0-5.8)	4.9±0.4	(4.2-5.5)	5.3±0.7	(4.3-6.5)	5.2±0.6	(4.3-5.9)	5.5±1.1	(4.4-6.5)
**AFB**	12.5	12.4±0.9	(9.8-14.2)	12.5±0.9	(10.3-14.2)	12.3±0.9	(10.3-14.0)	12.8±0.8	(11.1-14.2)	12.1±1.0	(9.8-13.3)	12.1±1.1	(9.8-12.9)	12.2±1.0	(11.4-13.3)
**AFAe**	14	12.3±0.9	(9.8-13.8)	12.2±0.7	(10.5-13.3)	11.9±0.7	(10.5-12.7)	12.5±0.7	(10.9-13.3)	12.5±1.3	(9.8-13.8)	12.6±1.4	(9.8-13.8)	12.4±1.3	(11.2-13.7)
**AdFB**	4.8	4.4±0.6	(3.4-5.5)	4.3±0.6	(3.4-5.5)	4.3±0.6	(3.5-5.5)	4.2±0.5	(3.4-4.7)	4.7±0.5	(3.8-5.4)	4.6±0.5	(3.8-5.1)	4.9±0.5	(4.5-5.4)
**CF**	na	24±1.4	(19.1-26.3)	24±1	(21.9-26)	23.8±0.7	(22.7-24.8)	24.3±1.2	(21.9-26)	24.1±2.2	(19.1-26.3)	24±2.6	(19.1-26.3)	24.3±1.5	(22.9-25.9)
**CD**	8.8	8.1±0.5	(6.7-8.9)	8.2±0.4	(7.6-8.9)	8.1±0.3	(7.6-8.6)	8.3±0.4	(7.8-8.9)	7.8±0.6	(6.7-8.6)	7.8±0.6	(6.7-8.6)	7.8±0.8	(7-8.4)
**CL**	12.6	12.6±0.8	(11.2-14.4)	12.6±0.9	(11.2-14.4)	12.5±0.8	(11.2-13.6)	12.9±0.9	(11.8-14.4)	12.5±0.9	(11.3-13.9)	12.5±0.8	(11.3-13.6)	12.5±1.2	(11.5-13.9)
**PAdC**	18.4	17.7±1.1	(15.3-19.5)	17.8±1	(15.7-19.4)	17.8±1.1	(15.7-19.4)	17.8±1	(16.2-19.4)	17.6±1.3	(15.3-19.5)	17.3±1.4	(15.3-19.5)	18.3±1.1	(17.2-19.3)
**DHL**	15.3	14.6±0.9	(12.6-16.3)	14.2±0.6	(12.6-15.6)	14.1±0.8	(12.6-15.6)	14.3±0.5	(13.7-15.0)	15.4±0.8	(14.0-16.3)	15.2±0.9	(14.0-16.3)	15.8±0.2	(15.6-16.0)
**PreP**	53.6	51.5±2.3	(42.7-55.2)	51.9±1.8	(48-55.2)	51.8±1.8	(49.3-55.2)	52.1±1.9	(48.0-54.5)	50.5±3.1	(42.7-53.0)	50.1±3.8	(42.7-53.0)	51.4±0.7	(50.6-51.9)
**PreA**	77.2	78.3±1.5	(75.2-81.8)	78.5±1.4	(75.2-81.1)	78.8±1.3	(76.5-81.1)	78.1±1.5	(75.2-80.3)	77.8±1.8	(76.1-81.8)	78.2±2.0	(76.1-81.8)	76.8±0.3	(76.5-77.0)
**PreD**	45	47.8±2	(39.9-50.3)	48±1.5	(45.5-50.3)	48.3±1.5	(45.5-50.3)	47.6±1.7	(45.6-50)	47.3±3	(39.9-50.2)	47.1±3.6	(39.9-50.2)	47.8±1.6	(46.3-49.5)
**BD**	27.6	26.5±2.7	(19.9-31.8)	27.5±1.9	(24.5-31.8)	28.2±2.0	(25.2-31.8)	26.5±1.4	(24.5-28.8)	24.2±2.9	(19.9-27.0)	24.8±2.8	(19.9-27.0)	23.1±3.3	(20.5-26.8)
**PostD**	43.7	42.6±2	(34.7-46)	42.7±1.3	(38.3-43.9)	42.3±1.5	(38.3-43.9)	43.2±0.5	(42.1-43.8)	42.3±3.3	(34.7-46)	42.3±4.1	(34.7-46)	42.5±0.9	(41.4-43)
**TL**	118	120.5±4.2	(100-124.2)	121±1.3	(118.7-123.4)	120.6±1.5	(118.7-123.4)	121.5±0.7	(120.8-122.8)	119.2±7.5	(100-124.2)	118.6±9.3	(100-124.2)	120.5±1.7	(118.9-122.4)
**HL (mm)**	58.6	54.2±10.6	(31.1-73.1)	58.3±8.6	(41.9-73.1)	60.2±6.8	(53-73.1)	55.7±10.4	(41.9-72.2)	44.6±8.7	(31.1-58.3)	49.5±4.7	(45.7-58.3)	34.9±6	(31.1-41.8)
**Percentage of head length**															
**SN**	22.2	23.1 ± 1.6	(20.1-26.4)	23.5 ± 1.5	(20.9-26.4)	23.7 ± 1.5	(21.4-26.4)	23.2 ± 1.5	(20.9-25.3)	22.4 ± 1.6	(20.1-25)	22.8 ± 1.7	(20.3-25)	21.7 ± 1.4	(20.1-22.7)
**ED**	22.1	22.5 ± 2	(19.5-27.2)	21.7 ± 1.5	(19.5-26.1)	21.3 ± 1.1	(19.5-22.9)	22.3 ± 2	(19.8-26.1)	24.3 ± 1.7	(21.8-27.2)	23.3 ± 1.1	(21.8-24.9)	26.1 ± 1	(25.4-27.2)
**EC**	28.9	27.4 ± 1.7	(24.5-31.5)	26.7 ± 1.2	(24.5-29)	26.6 ± 1	(24.9-28.3)	26.9 ± 1.4	(24.5-29)	29 ± 1.9	(26.4-31.5)	28.1 ± 1.7	(26.4-30.6)	30.6 ± 0.8	(29.9-31.5)
**Morphological characters**	*** C. alpinus***	**Both lakes**		**Lake Thun**						**Lake Brienz**					
	**Lectotype**	**Non-types both sexes**		**Non-types**						**Non-types**					
		***N-total* = 30**		***N-total* = 21**		***N-females* = 12**		***N-males* = 9**		***N-total* = 9**		***N-females* = 6**		***N-males* = 3**	
		**Mean ± StDev**	**Range**	**Mean ± StDev**	**Range**	**Mean ± StDev**	**Range**	**Mean ± StDev**	**Range**	**Mean ± StDev**	**Range**	**Mean ± StDev**	**Range**	**Mean ± StDev**	**Range**
**EH**	23.7	22.2 ± 1.8	(19.1-27.1)	21.4 ± 1.4	(19.1-23.6)	21.1 ± 1.2	(19.1-23)	21.9 ± 1.6	(19.6-23.6)	23.9 ± 1.5	(22.4-27.1)	23.3 ± 0.9	(22.4-24.6)	25.1 ± 2	(23.2-27.1)
**ES**	5.7	5.0 ± 0.8	(3.3-6.3)	5.1 ± 0.8	(3.4-6.3)	5.0 ± 0.7	(3.7-6.0)	5.2 ± 0.9	(3.4-6.3)	4.7 ± 0.8	(3.3-5.8)	4.6 ± 0.9	(3.3-5.5)	4.9 ± 0.8	(4.3-5.8)
**PostO**	51.2	52.4 ± 1.5	(48.9-55.4)	52.6 ± 1.2	(50.2-55.4)	52.5 ± 1.3	(50.8-55.4)	52.8 ± 1.2	(50.2-54.4)	51.7 ± 1.9	(48.9-54.8)	52.2 ± 1.5	(50.7-54.8)	50.8 ± 2.6	(48.9-53.8)
**HD**	71.7	71 ± 3.7	(65.5-79.6)	71.6 ± 4	(65.6-79.6)	71.5 ± 4.6	(65.6-79.6)	71.8 ± 3.4	(67.9-76.7)	69.4 ± 2.5	(65.5-73.2)	69.7 ± 2.7	(65.5-73.2)	68.7 ± 2.3	(66.3-70.8)
**MW**	8.9	9.4 ± 0.5	(8.4-10.4)	9.4 ± 0.5	(8.4-10.4)	9.3 ± 0.6	(8.4-10.4)	9.4 ± 0.6	(8.8-10.2)	9.5 ± 0.5	(8.7-10.2)	9.6 ± 0.5	(9.2-10.2)	9.3 ± 0.5	(8.7-9.7)
**UJ**	27.8	27.4 ± 1.5	(24.3-30.1)	27.7 ± 1.5	(24.3-30.1)	27.2 ± 1.5	(24.3-29.4)	28.4 ± 1.3	(26.5-30.1)	26.8 ± 1.3	(25.4-29.1)	26.5 ± 1	(25.6-28.2)	27.3 ± 1.8	(25.4-29.1)
**LJ**	38.2	38.4 ± 1.7	(33.8-41.4)	38.6 ± 1.7	(36.6-41.4)	38.5 ± 1.8	(36.6-41.4)	38.7 ± 1.6	(37-41.2)	38.2 ± 1.8	(33.8-39.4)	38 ± 2.1	(33.8-39.2)	38.5 ± 1.3	(36.9-39.4)
**M**	22.5	20 ± 1.2	(16.6-22.5)	20 ± 1.1	(17.7-22.1)	19.7 ± 0.9	(18.4-21)	20.3 ± 1.3	(17.7-22.1)	20 ± 1.6	(16.6-22.5)	19.6 ± 1.6	(16.6-21.1)	20.8 ± 1.5	(19.5-22.5)
**SD**	9.9	10.1 ± 1.4	(7.2-12.9)	10.5 ± 1.3	(8.5-12.9)	10.5 ± 1.4	(8.5-12.9)	10.6 ± 1.2	(8.7-12.2)	9.2 ± 1.1	(7.2-10.5)	9.2 ± 0.8	(7.9-10)	9.3 ± 1.9	(7.2-10.5)
**SW**	13.8	15.6 ± 1.1	(13.7-17.6)	15.6 ± 1.2	(13.7-17.6)	15.7 ± 1.1	(14.1-17.6)	15.5 ± 1.4	(13.7-17.2)	15.7 ± 0.9	(14.6-17.6)	15.6 ± 0.8	(14.6-16.7)	16 ± 1.3	(15.3-17.6)
**HW**	47.1	50 ± 4.5	(39.2-59.5)	51.3 ± 4.1	(44.2-59.5)	51.5 ± 5	(44.2-59.5)	51 ± 2.8	(46.3-55.9)	46.9 ± 4	(39.2-52.3)	47.4 ± 5	(39.2-52.3)	45.8 ± 0.4	(45.4-46.1)
**IOW**	24.5	27.7 ± 2.2	(22.4-32.5)	28.3 ± 2.3	(22.4-32.5)	28 ± 2.4	(22.4-32.4)	28.7 ± 2.3	(24.9-32.5)	26.5 ± 1.2	(24.9-28)	26.5 ± 1.4	(24.9-28)	26.6 ± 1	(25.5-27.4)
**INW**	10.7	11.7 ± 1	(9.5-14.1)	12.1 ± 1	(10.5-14.1)	12 ± 1.1	(10.5-14.1)	12.1 ± 0.9	(10.7-14)	11 ± 0.7	(9.5-11.9)	10.9 ± 0.9	(9.5-11.9)	11.2 ± 0.4	(10.9-11.6)
**LJW**	10.3	11.1 ± 2.2	(7.3-15.7)	12.1 ± 1.7	(10.1-15.7)	11.9 ± 1.8	(10.1-15.4)	12.4 ± 1.6	(10.5-15.7)	8.8 ± 1.2	(7.3-10.6)	9 ± 1.2	(7.5-10.6)	8.5 ± 1.4	(7.3-10.1)
**UJW**	19.8	23.2 ± 2.2	(18.4-27.2)	23.5 ± 2.2	(19.9-27.2)	23 ± 2.5	(19.9-27.2)	24.1 ± 1.6	(21.9-26.5)	22.8 ± 2.2	(18.4-25.6)	23.7 ± 1.6	(21.4-25.6)	20.9 ± 2.2	(18.4-22.8)
**MGR**	10.6	10.9 ± 1.4	(8.3-15.2)	11.3 ± 1.4	(9.3-15.2)	11.3 ± 1.4	(9.3-15.2)	11.3 ± 1.4	(9.5-13.2)	9.8 ± 1	(8.3-11.2)	9.9 ± 1.1	(8.3-11.2)	9.7 ± 0.8	(8.7-10.2)
**LGR**	11.5	11.9 ± 1.2	(10-15.2)	12.3 ± 1.1	(10.6-15.2)	12.2 ± 1.2	(10.6-15.2)	12.5 ± 1	(11.3-14.1)	10.8 ± 0.7	(10-12.3)	10.9 ± 0.8	(10-12.3)	10.6 ± 0.6	(10-11.2)
**UA**	na	18.5 ± 1.4	(15.6-21.5)	18.4 ± 1.3	(15.6-20.7)	18.6 ± 1.5	(15.6-20.7)	18.2 ± 1.1	(16.1-19.7)	18.7 ± 1.7	(16.4-21.5)	18.2 ± 1.4	(16.4-20)	19.8 ± 1.7	(18-21.5)
**LA**	35	33.9 ± 2.1	(28.6-38.8)	33.5 ± 1.8	(28.6-36.3)	34.1 ± 1.5	(30.9-36.3)	32.7 ± 1.9	(28.6-35)	34.7 ± 2.7	(30.4-38.8)	34.5 ± 2.7	(30.4-38.2)	35.1 ± 3.2	(32.8-38.8)
**Meristic characters**		**Mode**	**Range**	**Mode**	**Range**	**Mode**	**Range**	**Mode**	**Range**	**Mode**	**Range**	**Mode**	**Range**	**Mode**	**Range**
**PelvF unbranched**	1	1	(1-1)	1	(1-1)	1	(1-1)	1	(1-1)	1	(1-1)	1	(1-1)	1	(1-1)
**PelvF branched**	11	11	(10-11)	11	(10-11)	11	(10-11)	11	(10-11)	10	(10-11)	10	(10-11)	11	(10-11)
**PecF unbranched**	1	1	(1-1)	1	(1-1)	1	(1-1)	1	(1-1)	1	(1-1)	1	(1-1)	1	(1-1)
**PecF branched**	16	15	(14-17)	15	(14-17)	15	(14-17)	16	(14-16)	15	(15-17)	15	(15-16)	na	(15-17)
**DF unbranched**	3	4	(3-4)	4	(3-4)	4	(3-4)	4	(3-4)	4	(3-4)	4	(3-4)	4	(4-4)
**DF branched**	11	11	(10-13)	11	(10-13)	11	(10-13)	11	(10-12)	11	(10-11)	11	(10-11)	11	(11-11)
**AF unbranched**	3	3	(3-4)	3	(3-4)	3	(3-3)	3	(3-4)	4	(3-4)	4	(3-4)	4	(3-4)
**Meristic characters**	*** C. alpinus***	**Both lakes**		**Lake Thun**						**Lake Brienz**					
	**Lectotype**	**Non-types both sexes**		**Non-types**						**Non-types**					
		***N-total* = 30**		***N-total* = 21**		***N-females* = 12**		***N-males* = 9**		***N-total* = 9**		***N-females* = 6**		***N-males* = 3**	
		**Mode**	**Range**	**Mode**	**Range**	**Mode**	**Range**	**Mode**	**Range**	**Mode**	**Range**	**Mode**	**Range**	**Mode**	**Range**
**AF branched**	12	12	(10-14)	12	(10-14)	12	(10-14)	12	(10-13)	11	(11-13)	12	(11-13)	11	(11-11)
**LS**	82	84	(77-93)	81	(77-93)	84	(77-93)	80	(78-84)	84	(80-88)	86	(80-88)	na	(81-84)
**PDS**	39	36	(32-42)	36	(32-42)	36	(32-42)	33	(33-38)	33	(32-42)	na	(33-42)	na	(32-37)
**TDS**	10	10	(8-11)	10	(9-11)	10	(9-11)	10	(9-11)	10	(8-10)	10	(8-10)	9	(9-10)
**TAS**	8	8	(7-9)	8	(8-9)	8	(8-9)	8	(8-9)	8	(7-8)	8	(7-8)	8	(7-8)
**TPS**	8	8	(7-9)	9	(8-9)	9	(8-9)	8	(8-9)	8	(7-8)	8	(8-8)	8	(7-8)
**UGR**	10	10	(8-11)	10	(8-11)	10	(9-11)	11	(8-11)	9	(9-11)	9	(9-11)	na	(9-11)
**LGR**	18	19	(15-23)	19	(16-23)	19	(16-21)	20	(17-23)	18	(15-21)	17	(17-21)	na	(15-19)
**total GR**	28	29	(25-34)	30	(25-34)	28	(26-32)	30	(25-34)	28	(26-30)	29	(27-30)	28	(26-28)

**Table 5.** Morphological and meristic data of *C. fatioi* Kottelat, 1997 from lakes Thun and Brienz, MHNG-809.059 lectotype from Lake Thun; non-type material N = 30 from Lake Thun and N = 30 from Lake Brienz.

**Morphological characters**	*** C. fatioi***	**Both lakes**		**Lake Thun**						**Lake Brienz**					
	**Lectotype**	**Non-types both sexes**		**Non-types**						**Non-types**					
		*** Ntotal * = 60**		*** Ntotal * = 30**		***Nfemales* = 17**		***Nmales* = 13**		*** Ntotal * = 30**		***Nfemales* = 12**		***Nmales* = 18**	
		**Mean ± StDev**	**Range**	**Mean ± StDev**	**Range**	**Mean ± StDev**	**Range**	**Mean ± StDev**	**Range**	**Mean ± StDev**	**Range**	**Mean ± StDev**	**Range**	**Mean ± StDev**	**Range**
**SL (mm)**	154.5	207.5±35.2	(132-288)	230.2±21.2	(191-288)	226.6±14.3	(191-245)	234.9±27.8	(202-288)	184.8±31.7	(132-244)	195.9±29.7	(141-244)	177.3±31.6	(132-225)
**Percentage of standard length**															
**PelvFB**	3.6	3.8±0.4	(3.1-4.8)	4.0±0.4	(3.2-4.8)	3.9±0.3	(3.3-4.4)	4.0±0.4	(3.2-4.8)	3.7±0.4	(3.1-4.6)	3.7±0.3	(3.1-4.1)	3.7±0.4	(3.2-4.6)
**PelvFS**	5.4	6.2±0.8	(3.9-8.0)	6.2±0.9	(3.9-8.0)	6.3±0.8	(4.6-7.4)	6.1±1.0	(3.9-8.0)	6.2±0.8	(3.9-7.4)	6.2±0.8	(3.9-7.0)	6.2±0.9	(3.9-7.4)
**PelvF**	17.4	16.1±1	(13.7-19.3)	16.4±1.2	(13.7-19.3)	16.6±1	(15.1-19.3)	16.1±1.4	(13.7-18.6)	15.8±0.8	(14.6-17.4)	15.8±0.7	(14.7-17.1)	15.9±0.8	(14.6-17.4)
**PecFB**	3.1	3.2±0.3	(2.7-3.8)	3.3±0.3	(2.8-3.8)	3.2±0.3	(2.8-3.7)	3.4±0.3	(2.9-3.8)	3.1±0.2	(2.7-3.4)	3.0±0.2	(2.7-3.4)	3.1±0.2	(2.7-3.4)
**PecF1**	17.8	16.2±1.3	(13.3-18.9)	16.5±1.4	(13.3-18.9)	16.8±1.2	(14.7-18.9)	16.1±1.5	(13.3-18.4)	16.0±1.1	(14.1-18.7)	15.8±1.0	(14.2-17.3)	16.0±1.2	(14.1-18.7)
**PecF2**	18.4	17.2±1.4	(13.8-20.6)	17.7±1.5	(13.8-20.6)	18±1.4	(15.5-20.6)	17.2±1.7	(13.8-19.8)	16.8±1.1	(14.9-19.7)	16.5±0.9	(14.9-17.9)	17±1.2	(15.3-19.7)
**DFB**	11.4	11.9±0.7	(10.3-13.3)	11.8±0.7	(10.3-13.1)	11.6±0.7	(10.3-12.9)	12.0±0.6	(11.2-13.1)	12.0±0.7	(10.3-13.3)	12.0±0.4	(11.3-12.6)	12.0±0.9	(10.3-13.3)
**DFAe**	na	17.9±1.3	(15.4-21.9)	18.0±1.4	(15.4-21.9)	18.4±1.4	(15.4-21.9)	17.4±1.2	(15.8-19.8)	17.8±1.1	(15.7-20.0)	17.6±1.0	(15.7-19.4)	18.0±1.2	(16.5-20.0)
**DFAd**	19.1	19.1±1.3	(16.7-23.5)	19.3±1.4	(16.7-23.5)	19.7±1.4	(16.7-23.5)	18.8±1.3	(17.0-21.3)	18.8±1.1	(17.1-21.1)	18.8±0.9	(17.1-20.3)	18.8±1.3	(17.1-21.1)
**DFPe**	6.3	5.1±0.6	(3.9-7.0)	5.0±0.7	(3.9-6.9)	5.1±0.7	(3.9-6.9)	4.8±0.7	(4.1-6.3)	5.3±0.5	(4.3-7.0)	5.3±0.5	(4.7-6.3)	5.3±0.6	(4.3-7.0)
**AFB**	12.1	12.3±0.9	(10.6-15.1)	12.6±1.0	(10.7-15.1)	12.5±1.0	(10.7-14.0)	12.8±1.1	(11.7-15.1)	11.9±0.8	(10.6-13.3)	12.0±0.6	(11.4-13.0)	11.9±0.8	(10.6-13.3)
**AFAe**	na	11.5±0.9	(9.8-13.9)	11.9±0.9	(10.2-13.9)	12.1±0.9	(10.5-13.9)	11.7±1.0	(10.2-13.3)	11.1±0.6	(9.8-12.7)	11.0±0.5	(9.8-11.9)	11.1±0.7	(10.2-12.7)
**AdFB**	5.7	5.5±0.8	(4.0-8.1)	5.6±0.7	(4.6-8.1)	5.7±0.7	(4.8-8.1)	5.4±0.6	(4.6-6.9)	5.5±0.8	(4.0-7.7)	5.5±0.7	(4.3-6.7)	5.5±0.9	(4.0-7.7)
**CF**	na	23.8±1.3	(19.6-27.2)	23.6±1.3	(19.6-26.6)	23.9±1.1	(22.1-26.4)	23.3±1.6	(19.6-26.6)	24±1.2	(22.4-27.2)	23.8±1.3	(22.4-27.2)	24.1±1.1	(22.7-26.4)
**CD**	7.7	7.2±0.3	(6.7-8.5)	7.1±0.3	(6.7-8.5)	7.1±0.4	(6.7-8.5)	7.2±0.2	(6.9-7.7)	7.3±0.3	(6.8-8)	7.3±0.3	(6.8-7.8)	7.4±0.3	(6.9-8)
**CL**	13.9	13.7±0.9	(11.5-16.1)	13.3±0.8	(11.5-14.7)	13.1±0.8	(11.5-14.7)	13.5±0.6	(12.6-14.6)	14.2±0.8	(13.1-16.1)	13.9±0.7	(13.1-15.5)	14.3±0.8	(13.2-16.1)
**PAdC**	19.1	18.9±1	(16.8-22.2)	18.8±1	(16.8-22.2)	18.6±1.1	(16.8-22.2)	19±0.8	(17.8-20)	19±1.1	(16.9-20.8)	18.7±1.1	(16.9-20.4)	19.1±1.1	(17.2-20.8)
**DHL**	15.9	15.2±0.8	(13.6-16.8)	14.8±0.7	(13.6-16.2)	14.9±0.6	(13.9-16.1)	14.6±0.9	(13.6-16.2)	15.7±0.7	(14.5-16.8)	15.4±0.8	(14.5-16.8)	15.8±0.6	(14.7-16.7)
**PreP**	52.1	52.0±1.5	(47.5-55.5)	51.8±1.7	(47.5-55.1)	52.2±1.7	(47.5-55.1)	51.3±1.6	(48.5-53.8)	52.1±1.3	(48.8-55.5)	52.7±1.4	(51.3-55.5)	51.8±1.1	(48.8-53.9)
**PreA**	78.8	77.0±1.3	(74.4-80.2)	76.9±1.5	(74.4-80.2)	77.4±1.6	(74.7-80.2)	76.3±1.1	(74.4-78.1)	77.1±1.0	(75.2-79.2)	77.4±0.9	(76.4-79.2)	76.8±1.0	(75.2-78.6)
**PreD**	48.4	46.8±1.3	(41.5-49.1)	46.8±1.5	(41.5-49.1)	46.5±1.7	(41.5-48.8)	47.2±1.1	(44.8-49.1)	46.8±1	(43.4-49)	46.7±1.4	(43.4-49)	46.9±0.7	(45.9-48)
**BD**	24.9	24.4±1.4	(22.1-28.1)	24.9±1.4	(22.7-28.1)	25.3±1.3	(23.2-28.1)	24.4±1.4	(22.7-28.1)	23.9±1.2	(22.1-26.2)	24.7±1.1	(22.4-26.2)	23.4±0.9	(22.1-24.9)
**PostD**	45.4	44.5±1.4	(41.6-50.7)	44.9±1.7	(41.6-50.7)	44.5±2	(41.6-50.7)	45.3±1.2	(43.4-47.1)	44.2±0.9	(42.5-45.8)	44±0.8	(42.5-45.6)	44.3±1	(42.5-45.8)
**TL**	na	121.1±1.8	(117.3-126)	120.7±1.9	(117.3-124.2)	121.3±1.7	(117.3-124)	120±1.9	(117.4-124.2)	121.5±1.8	(118.8-126)	121.3±2.1	(119-126)	121.6±1.5	(118.8-124.2)
**HL (mm)**	35.1	43.5±6.5	(27.9-55.9)	47.5±3.4	(42-55.9)	47.4±2.8	(42.6-50.9)	47.7±4.2	(42-55.9)	39.5±6.3	(27.9-48.8)	41.3±5.1	(31.4-48.2)	38.3±6.9	(27.9-48.8)
**Percentage of head length**															
**SN**	19.9	23.6±1.9	(18.2-27)	24.3±1.6	(18.2-27)	24.2±2	(18.2-27)	24.5±1.1	(23-26.5)	22.8±1.9	(18.5-26.4)	23.2±1.4	(21.4-25.5)	22.5±2.2	(18.5-26.4)
**ED**	25.8	23.6±2	(19.9-27.6)	22.4±1.4	(19.9-25.9)	22.8±1.3	(20.9-25.9)	22±1.5	(19.9-24.7)	24.8±1.7	(21.2-27.6)	24.9±1	(22.9-26.6)	24.8±2.1	(21.2-27.6)
**EC**	31.8	27.6±2.2	(23.2-33)	26.3±1.6	(23.2-29)	26.6±1.5	(23.6-29)	25.9±1.7	(23.2-28.6)	29±1.9	(25.3-33)	29.1±0.9	(27.5-30.6)	28.9±2.4	(25.3-33)
**EH**	25.8	23.4±1.6	(19.7-26.3)	22.4±1.3	(19.7-25.3)	22.6±1.2	(20.8-25.3)	22.1±1.4	(19.7-25)	24.4±1.3	(22.1-26.3)	24.4±1.2	(22.8-26.2)	24.4±1.4	(22.1-26.3)
**ES**	3.6	4.3±1.2	(1.7-6.8)	3.6±1.1	(1.7-5.9)	3.3±0.9	(1.7-5.2)	4.1±1.1	(2.1-5.9)	4.9±0.9	(3.4-6.8)	4.9±0.8	(4.0-6.8)	4.9±1.1	(3.4-6.5)
**PostO**	49.8	51.3±1.8	(46.8-54.8)	52.2±1.4	(48.7-54.8)	52±1.6	(48.7-54.1)	52.5±1.1	(51.4-54.8)	50.5±1.7	(46.8-54)	50.7±1	(49.8-53)	50.3±2.1	(46.8-54)
**HD**	68.3	69.6±3.1	(63.6-78.6)	70.7±3.3	(65.5-78.6)	70.6±3.7	(65.5-78.6)	70.8±2.7	(66.3-74.8)	68.5±2.6	(63.6-73.2)	68.8±2.8	(65.7-73.2)	68.3±2.5	(63.6-72.2)
**MW**	11.5	10.1±0.8	(8.2-12.1)	10±0.9	(8.2-12.1)	10.1±0.9	(8.5-12.1)	9.8±0.9	(8.2-11)	10.1±0.8	(8.5-11.4)	10±0.9	(8.5-11.4)	10.2±0.8	(8.6-11.4)
**UJ**	30.5	30±1.4	(27.6-34.1)	30.5±1.5	(28-34.1)	30.8±1.6	(28-34.1)	30.2±1.3	(28.2-33.1)	29.5±1.3	(27.6-32)	29.4±1.2	(28.1-31.2)	29.6±1.3	(27.6-32)
**LJ**	38.4	41.6±2.6	(36.9-48.4)	40.7±2.1	(36.9-46.1)	41.3±2.1	(37.4-46.1)	39.8±1.8	(36.9-42.3)	42.6±2.6	(37.6-48.4)	43.1±2.3	(40.6-47.8)	42.2±2.9	(37.6-48.4)
**M**	26.6	21.8±1.3	(18.5-25.6)	21.8±1.6	(18.5-25.6)	22.1±1.4	(19.8-25.6)	21.5±1.7	(18.5-25.1)	21.7±1	(18.7-24.2)	22±0.8	(21-23.8)	21.5±1.1	(18.7-24.2)
**SD**	8.1	9.3±1.2	(6.7-12.4)	9.5±1.2	(6.7-12.4)	9±1	(6.7-10.5)	10.1±1.1	(8.7-12.4)	9±1.1	(6.7-10.9)	9.4±1.2	(6.9-10.9)	8.8±1	(6.7-10.4)
**SW**	15	17.7±1.3	(14.7-20.4)	17.5±1.3	(14.7-20.4)	17.6±1.5	(14.7-20.4)	17.5±1.1	(16-19.6)	17.8±1.3	(14.7-19.7)	17.4±1.6	(14.7-19.7)	18±1.1	(15.8-19.7)
**HW**	45.2	49.8±3.1	(42.3-57.2)	51.1±3	(45.8-56.6)	51.5±3.4	(45.8-56.6)	50.6±2.3	(47.8-54.1)	48.5±2.7	(42.3-57.2)	48.8±3.5	(42.3-57.2)	48.3±2	(44.8-52.8)
**IOW**	24.5	27±1.5	(22.8-31.5)	27.7±1.6	(23.6-31.5)	27.2±1.4	(23.6-29.7)	28.3±1.6	(25.4-31.5)	26.4±1.1	(22.8-28.8)	26.6±0.9	(25.4-28.8)	26.2±1.3	(22.8-28.5)
**INW**	10.9	11.5±1.1	(9.2-13.5)	11.7±1	(9.8-13.5)	11.6±1.1	(9.8-13.3)	11.8±0.9	(10.6-13.5)	11.4±1.2	(9.2-13.5)	11.2±1	(10.1-13.3)	11.5±1.3	(9.2-13.5)
**LJW**	14.7	12±1.9	(7.9-16)	12.4±2.3	(7.9-16)	12.5±2.2	(7.9-15.8)	12.4±2.5	(7.9-16)	11.6±1.2	(8.6-13.3)	11.4±1.3	(8.7-13.3)	11.8±1.2	(8.6-13.2)
**UJW**	23.7	24±1.8	(20.3-30.3)	24.7±1.8	(21-30.3)	25.2±2	(21-30.3)	24.1±1.3	(22.1-26.8)	23.4±1.5	(20.3-26.5)	23.1±1.7	(20.3-26.5)	23.5±1.5	(21-26)
**MGR**	14.3	14.5±2	(10.5-21.3)	15.8±1.9	(12.5-21.3)	16.3±1.8	(13.9-21.3)	15±1.8	(12.5-19.6)	13.2±1.2	(10.5-15)	13±1	(11.5-14.4)	13.3±1.4	(10.5-15)
**LGR**	14.9	15.6±2.1	(12.3-22.6)	16.9±2	(12.8-22.6)	17.5±1.9	(15.4-22.6)	16.1±1.9	(12.8-19.6)	14.3±1.1	(12.3-16.4)	14.3±0.9	(12.7-15.5)	14.3±1.2	(12.3-16.4)
**UA**	19	18.6±1.5	(15.7-22.6)	19.1±1.6	(16.1-22.6)	18.6±1.4	(16.1-21.2)	19.7±1.7	(17.3-22.6)	18.2±1.1	(15.7-20.1)	18±0.8	(16.7-19.4)	18.4±1.3	(15.7-20.1)
**LA**	35.3	35.8±1.8	(32.5-41.3)	35.9±1.7	(32.5-41.3)	36.1±1.4	(34-38.6)	35.7±2.1	(32.5-41.3)	35.7±1.9	(32.9-39.8)	35.7±1.2	(33.4-37.3)	35.8±2.3	(32.9-39.8)
**Meristic characters**		**Mode**	**Range**	**Mode**	**Range**	**Mode**	**Range**	**Mode**	**Range**	**Mode**	**Range**	**Mode**	**Range**	**Mode**	**Range**
**PelvF unbranched**	1	1	(1-1)	1	(1-1)	1	(1-1)	1	(1-1)	1	(1-1)	1	(1-1)	1	(1-1)
**PelvF branched**	11	10	(9-11)	10	(9-11)	10	(9-11)	10	(9-11)	10	(9-11)	10	(10-11)	10	(9-11)
**PecF unbranched**	1	1	(1-1)	1	(1-1)	1	(1-1)	1	(1-1)	1	(1-1)	1	(1-1)	1	(1-1)
**PecF branched**	16	16	(14-17)	16	(14-17)	16	(14-17)	16	(15-17)	16	(14-17)	16	(14-17)	16	(14-17)
**DF unbranched**	4	4	(3-4)	4	(3-4)	4	(3-4)	4	(3-4)	4	(3-4)	4	(3-4)	4	(3-4)
**DF branched**	10	10	(10-13)	10	(10-11)	10	(10-11)	10	(10-11)	11	(10-13)	11	(10-12)	11	(10-13)
**AF unbranched**	3	3	(2-5)	3	(2-4)	3	(2-4)	2	(2-4)	4	(3-5)	3	(3-5)	4	(3-4)
**AF branched**	12	12	(10-14)	12	(10-14)	12	(11-14)	12	(10-14)	12	(11-13)	12	(11-13)	12	(11-13)
**LS**	82	86	(78-93)	86	(78-93)	85	(78-86)	86	(78-93)	86	(79-92)	86	(81-92)	86	(79-91)
**PDS**	32	36	(30-44)	32	(30-44)	36	(30-40)	38	(31-44)	34	(30-40)	34	(30-38)	34	(33-40)
**TDS**	10	9	(8-11)	9	(8-11)	9	(9-10)	10	(8-11)	10	(9-10)	10	(9-10)	10	(9-10)
**TAS**	7	8	(7-10)	8	(7-9)	8	(7-9)	8	(7-9)	8	(7-9)	8	(8-9)	8	(7-9)
**TPS**	8	9	(7-10)	9	(7-9)	9	(8-9)	9	(7-9)	9	(8-9)	9	(8-9)	9	(8-9)
**UGR**	11	12	(10-16)	14	(10-16)	14	(12-16)	14	(10-15)	12	(11-15)	12	(11-14)	12	(11-15)
**LGR**	22	24	(19-27)	24	(22-27)	24	(22-27)	24	(22-26)	22	(19-27)	22	(22-26)	24	(19-27)
**total GR**	33	38	(32-43)	38	(32-43)	38	(34-43)	38	(32-40)	35	(32-40)	37	(33-38)	39	(32-40)

**Table 6.** Morphological and meristic data of *C. steinmanni* from Lake Thun, Switzerland, NMBE-1077219, female, holotype from Lake Thun; paratypes *N* = 12. For females and for both sexes the range and mean include the holotype.

**Morphological characters**	*** C. steinmanni***	**Lake Thun**					
	**Holotype**	**Both sexes**					
		***N-total* = 12**		***N-females* = 3**		***N-males* = 9**	
		**Mean ± Stdev**	**Range**	**Mean ± Stdev**	**Range**	**Mean ± Stdev**	**Range**
**SL (mm)**	301	275.3±29.4	(211-323)	276.5±36.9	(234-301)	274.9±29.2	(211-323)
**Percentage of standard length**							
**PelvFB**	4.0	4.4±0.3	(4.0-4.8)	4.1±0.2	(4.0-4.3)	4.5±0.3	(4.1-4.8)
**PelvFS**	5.7	6.2±0.5	(5.3-6.9)	6.2±0.4	(5.7-6.5)	6.2±0.6	(5.3-6.9)
**PelvF**	15.3	16.5±1.1	(14.6-18.3)	16.1±1.1	(15.3-17.4)	16.6±1.1	(14.6-18.3)
**PecFB**	3.2	3.4±0.3	(3.1-3.8)	3.2±0.2	(3.1-3.4)	3.4±0.3	(3.1-3.8)
**PecF1**	14.7	16.2±1.3	(13.9-18.2)	15.8±1.1	(14.7-16.9)	16.4±1.4	(13.9-18.2)
**PecF2**	15.2	17±1.3	(15.2-19.1)	16.2±1	(15.2-17.2)	17.3±1.3	(15.5-19.1)
**DFB**	11.4	12.6±0.8	(11.4-13.8)	12.4±1.2	(11.4-13.7)	12.6±0.7	(11.7-13.8)
**DFAe**	16.2	18.8±1.7	(16.2-21.2)	18.4±2.3	(16.2-20.9)	19.0±1.6	(16.2-21.2)
**DFAd**	17.8	20.1±1.6	(17.5-22.4)	20.0±2.3	(17.8-22.4)	20.2±1.4	(17.5-22.1)
**DFPe**	4.8	4.8±0.7	(3.9-6.3)	4.6±0.2	(4.4-4.8)	4.9±0.7	(3.9-6.3)
**AFB**	12.1	12.6±0.8	(11.5-14.2)	12.6±0.5	(12.1-13.0)	12.6±0.9	(11.5-14.2)
**AFAe**	11.2	12.4±1.0	(10.8-13.7)	12.3±1.3	(11.2-13.7)	12.4±0.9	(10.8-13.5)
**AdFB**	5	4.5±0.6	(3.7-5.4)	4.4±0.5	(4.0-5.0)	4.5±0.6	(3.7-5.4)
**CF**	23.6	23.4±1.2	(22.2-25.9)	23.7±1.2	(22.4-24.9)	23.3±1.3	(22.2-25.9)
**CD**	7.8	8±0.4	(7.5-8.6)	7.9±0.2	(7.7-8.1)	8±0.4	(7.5-8.6)
**CL**	13	13.0±0.7	(11.4-14.0)	13.5±0.5	(13.0-14.0)	12.9±0.7	(11.4-13.9)
**PAdC**	18.2	18±1	(16.4-19.6)	17.8±0.8	(16.9-18.4)	18±1.1	(16.4-19.6)
**DHL**	13.6	14.0±0.7	(13.2-15.1)	14.3±0.7	(13.6-14.9)	13.9±0.7	(13.2-15.1)
**PreP**	53.4	51.7±1.9	(48.6-54.3)	52.8±1.2	(51.4-53.6)	51.3±2.0	(48.6-54.3)
**PreA**	78.1	77.5±0.9	(75.0-78.4)	78.0±0.6	(77.4-78.4)	77.3±0.9	(75.0-77.9)
**PreD**	50	47.2±1.5	(44.5-50)	48.2±1.7	(46.7-50)	46.9±1.4	(44.5-49.7)
**BD**	30	27.0±1.5	(24.6-30.0)	28.0±1.8	(26.5-30.0)	26.7±1.3	(24.6-28.7)
**PostD**	43.3	43.3±1.2	(41.9-45.6)	42.5±0.8	(41.9-43.3)	43.6±1.2	(42-45.6)
**TL**	120.1	119.6±2.3	(115.3-122.5)	119.5±0.6	(118.8-120.1)	119.6±2.7	(115.3-122.5)
**HL (mm)**	58.7	55.3±4.9	(44.8-63.3)	55.6±5.3	(49.4-58.7)	55.2±5.1	(44.8-63.3)
**Percentage of head length**							
**SN**	22.2	23.2±1.7	(20.5-26.3)	23.5±1.2	(22.2-24.6)	23.1±1.9	(20.5-26.3)
**ED**	22.2	22±1.1	(20.5-24.5)	22.6±1.7	(21.1-24.5)	21.8±0.8	(20.5-23)
**EC**	25.5	26.2±1.2	(24.2-27.8)	26.3±1.3	(25.5-27.8)	26.2±1.2	(24.2-27.4)
**EH**	22.5	21.6±1.1	(19.6-24.1)	22.5±1.5	(21-24.1)	21.3±0.8	(19.6-22)
**ES**	5.1	4.8±0.6	(3.9-5.6)	4.9±0.5	(4.3-5.2)	4.8±0.7	(3.9-5.6)
**PostO**	54.4	52.4±1.4	(50.3-54.4)	53±1.4	(51.6-54.4)	52.2±1.5	(50.3-54.3)
**HD**	72.1	72.1±2.1	(68.9-76.3)	72.8±0.9	(72.1-73.8)	71.8±2.4	(68.9-76.3)
**MW**	10.7	9.3±0.7	(8.3-10.7)	9.7±0.8	(9.1-10.7)	9.2±0.7	(8.3-10.6)
**UJ**	27	27.3±1.4	(25.2-30)	27.3±0.7	(26.9-28.1)	27.3±1.6	(25.2-30)
**LJ**	39.4	39±1.2	(36.6-40.4)	39.7±0.3	(39.4-40)	38.7±1.3	(36.6-40.4)
**M**	19.7	19.7±1.2	(18.1-21.8)	19.4±0.7	(18.6-19.9)	19.8±1.3	(18.1-21.8)
**SD**	10.4	10±1.7	(6.5-13.2)	10.1±0.4	(9.7-10.4)	10±2	(6.5-13.2)
**SW**	15.8	16.7±1.1	(15.3-18.9)	16±0.8	(15.3-17)	16.9±1.1	(15.7-18.9)
**HW**	53.1	51.6±3.1	(44.5-56.9)	49±4.3	(44.5-53.1)	52.4±2.2	(49.5-56.9)
**IOW**	29.6	27.6±2.3	(23.8-31.2)	27.9±2.2	(25.4-29.6)	27.5±2.4	(23.8-31.2)
**INW**	11.6	12.1±0.7	(11-13.2)	11.7±0.1	(11.6-11.8)	12.3±0.7	(11-13.2)
**LJW**	14.3	11.9±1.4	(9.7-14.3)	12±2.3	(9.7-14.3)	11.9±1.1	(10.1-13.6)
**UJW**	24.1	23±1.6	(19.3-25)	21.6±2.4	(19.3-24.1)	23.4±1.1	(21.2-25)
**MGR**	11.3	11.5±1.7	(9.1-14.3)	11.3±1.1	(10.2-12.4)	11.5±1.9	(9.1-14.3)
**LGR**	11.7	12.1±1.5	(10-14.4)	11.6±1.2	(10.4-12.9)	12.3±1.6	(10-14.4)
**UA**	19.6	18.6±0.6	(17.8-19.8)	18.9±0.6	(18.4-19.6)	18.6±0.6	(17.8-19.8)
**LA**	34.7	34.3±1.2	(31.6-36.5)	33.9±0.8	(33-34.7)	34.4±1.3	(31.6-36.5)
**Meristic characters**		**Mode**	**Range**	**Mode**	**Range**	**Mode**	**Range**
**PelvF unbranched**	1	1	(1-1)	1	(1-1)	1	(1-1)
**PelvF branched**	10	10	(10-12)	na	(10-12)	10	(10-12)
**PecF unbranched**	1	1	(1-1)	1	(1-1)	1	(1-1)
**PecF branched**	15	15	(14-16)	na	(14-16)	15	(15-16)
**DF unbranched**	4	4	(3-4)	4	(3-4)	4	(3-4)
**DF branched**	10	10	(10-12)	10	(10-11)	10	(10-12)
**AF unbranched**	3	3	(3-3)	3	(3-3)	3	(3-3)
**AF branched**	11	12	(11-13)	12	(11-12)	12	(11-13)
**LS**	78	78	(78-87)	78	(78-80)	85	(78-87)
**PDS**	40	36	(32-40)	na	(32-40)	35	(33-40)
**TDS**	9	10	(8-10)	9	(9-10)	10	(8-10)
**TAS**	8	8	(8-9)	8	(8-8)	8	(8-9)
**TPS**	8	9	(8-9)	8	(8-8)	9	(8-9)
**UGR**	10	11	(10-12)	11	(10-11)	12	(10-12)
**LGR**	20	20	(19-23)	20	(20-21)	21	(19-23)
**total GR**	30	31	(30-35)	na	(30-32)	31	(30-35)

**Table 7.** Morphological and meristic data of *C. brienzii* from Lake Brienz, Switzerland, NMBE-1077126, female, holotype; paratypes N = 12. For females and for both sexes the range and the mean include the holotype.

**Morphological characters**	*** C. brienzii***	**Lake Brienz**					
	**Holotype**	**Both sexes**					
		***N-total* = 13**		***N-females* = 4**		***N-males* = 9**	
		**Mean ± Stdev**	**Range**	**Mean ± Stdev**	**Range**	**Mean ± Stdev**	**Range**
**SL (mm)**	223.0	181.5±37.0	(118–226)	187.8±47.4	(118–223)	178.7±34.3	(129–226)
**Percentage of standard length**							
**PelvFB**	4.1	3.7±0.6	(2.8–4.8)	3.6±0.6	(2.8–4.2)	3.8±0.6	(2.9–4.8)
**PelvFS**	6.1	6.1±0.8	(4.6–7.4)	5.8±0.4	(5.1–6.1)	6.3±0.9	(4.6–7.4)
**PelvF**	15.2	15.6±1.1	(14–17.5)	15±0.5	(14.6–15.7)	15.9±1.2	(14–17.5)
**PecFB**	3.1	3.1±0.2	(2.6–3.4)	3.0±0.3	(2.6–3.2)	3.1±0.2	(2.8–3.4)
**PecF1**	16.0	15.9±1.6	(13.9–20.1)	15.4±0.7	(14.5–16.0)	16.2±1.8	(13.9–20.1)
**PecF2**	17.0	16.8±1.6	(14–20.7)	16.4±0.7	(15.5–17)	17±1.9	(14–20.7)
**DFB**	12.3	11.8±0.8	(10.4–12.9)	11.7±0.4	(11.3–12.3)	11.9±1.0	(10.4–12.9)
**DFAe**	17.6	17.9±1.2	(15.5–19.8)	17.7±0.7	(16.9–18.6)	18.0±1.4	(15.5–19.8)
**DFAd**	18.7	18.6±1.5	(15.3–20.8)	18.3±0.4	(17.8–18.7)	18.8±1.8	(15.3–20.8)
**DFPe**	5.1	5.2±0.6	(4.2–6.5)	5.0±0.2	(4.7–5.2)	5.3±0.7	(4.2–6.5)
**AFB**	13.7	12.4±0.9	(11.1–13.7)	12.9±1.1	(11.4–13.7)	12.2±0.7	(11.1–13.6)
**AFAe**	11.1	11.2±1.0	(9.4–12.6)	11.2±0.5	(10.5–11.6)	11.2±1.2	(9.4–12.6)
**AdFB**	5.1	5.5±0.8	(4.0–7.1)	5.2±0.3	(5.0–5.7)	5.6±0.9	(4.0–7.1)
**CF**	23.8	24.1±1.1	(22.6–26.3)	23.2±0.5	(22.6–23.8)	24.5±1.1	(22.7–26.3)
**CD**	7.3	7.3±0.3	(6.7–7.7)	7.1±0.4	(6.7–7.5)	7.4±0.2	(7.1–7.7)
**CL**	13.9	13.8±1.0	(12.2–15.8)	14.0±0.4	(13.7–14.6)	13.7±1.1	(12.2–15.8)
**PAdC**	18.8	19.1±0.7	(17.9–20.7)	19±0.4	(18.6–19.4)	19.1±0.9	(17.9–20.7)
**DHL**	15.0	15.6±0.7	(14.6–16.8)	15.4±0.6	(15.0–16.3)	15.7±0.7	(14.6–16.8)
**PreP**	48.6	51.1±1.7	(47.8–54.0)	50.9±1.8	(48.6–52.8)	51.2±1.8	(47.8–54.0)
**PreA**	75.3	77.1±1.5	(74.3–79.5)	76.2±1.7	(74.3–78.2)	77.5±1.3	(75.4–79.5)
**PreD**	46.2	47.5±1.7	(43.9–49.4)	47.2±1.1	(46.2–48.2)	47.6±2	(43.9–49.4)
**BD**	24.6	22.6±1.7	(19.6–25.1)	22.7±2.5	(20.5–25.1)	22.6±1.5	(19.6–24.2)
**PostD**	45.9	44.1±1.1	(42.4–45.9)	44.6±1.3	(43–45.9)	43.9±1.1	(42.4–45.5)
**TL**	122.0	121.5±1.9	(117.8–124.4)	121.2±2.5	(117.8–123.8)	121.6±1.7	(119.2–124.4)
**HL (mm)**	45.4	38.7±7.3	(26.7–47.4)	39.2±8.5	(26.7–45.4)	38.5±7.3	(28.3–47.4)
**Percentage of head length**							
**SN**	25.6	23.3±1.8	(20.5–26.3)	23.6±2.1	(21.1–25.6)	23.2±1.7	(20.5–26.3)
**ED**	24.4	25.3±1.6	(23.1–28.3)	25.2±1.6	(24.2–27.6)	25.3±1.7	(23.1–28.3)
**EC**	27.8	29±2.3	(25.6–32.9)	28.8±3.1	(25.6–32.9)	29.1±2.1	(26.5–32.7)
**EH**	22.0	24.4±1.4	(22–27.2)	23.9±1.7	(22–26.2)	24.7±1.3	(23–27.2)
**ES**	3.5	4.7±1.2	(3.3–7.2)	4.8±1.4	(3.5–6.5)	4.7±1.2	(3.3–7.2)
**PostO**	50.9	50.7±1.1	(48.2–52.3)	49.8±1.5	(48.2–51.1)	51.1±0.7	(50.3–52.3)
**HD**	75.2	68.5±3.3	(64.4–75.2)	69.8±4.4	(65.2–75.2)	67.9±2.8	(64.4–73.1)
**MW**	9.7	9.9±0.9	(8.5–10.9)	9.4±0.8	(8.5–10.3)	10.1±0.8	(8.6–10.9)
**UJ**	30.2	29.5±1.6	(27.1–32)	29±1.8	(27.1–30.8)	29.6±1.6	(27.3–32)
**LJ**	42.9	42.2±1.5	(40.5–45.7)	43.2±1.7	(42–45.7)	41.8±1.2	(40.5–43.7)
**M**	23.4	21±2.4	(15.4–24)	21±3.9	(15.4–24)	21.1±1.7	(18.3–23.8)
**SD**	7.0	8.8±1.4	(6.4–11.6)	7.4±0.9	(6.4–8.6)	9.4±1.2	(8–11.6)
**SW**	18.0	17.8±1.2	(15.7–20.2)	17.6±0.6	(16.7–18)	17.8±1.4	(15.7–20.2)
**HW**	52.1	48.1±3.1	(44.1–52.4)	48.5±4	(44.1–52.1)	47.9±2.9	(44.1–52.4)
**IOW**	28.4	26.2±1.9	(22.8–30.7)	26.3±1.7	(25–28.4)	26.1±2.1	(22.8–30.7)
**INW**	9.7	11.1±0.8	(9.7–12.6)	10.8±0.7	(9.7–11.2)	11.3±0.9	(10–12.6)
**LJW**	14.1	11.5±1.2	(10.1–14.1)	11.9±2	(10.1–14.1)	11.3±0.6	(10.5–12.4)
**UJW**	25.9	23.4±1.6	(20.2–26.1)	23.3±2.3	(20.2–25.9)	23.5±1.3	(21.4–26.1)
**MGR**	13.5	13.5±1.3	(10.9–15.1)	13.7±1.6	(11.6–15.1)	13.3±1.3	(10.9–14.9)
**LGR**	13.9	14 .7±1.6	(12.1–16.8)	14.8±2.2	(12.1–16.8)	14.7±1.4	(13–16.7)
**UA**	20.4	18.5±1.7	(15.3–20.5)	19.6±0.8	(18.5–20.4)	18±1.7	(15.3–20.5)
**LA**	40.4	35.5±2	(33–40.4)	37.2±2.5	(35–40.4)	34.8±1.4	(33–37.5)
**Meristic characters**		**Mode**	**Range**	**Mode**	**Range**	**Mode**	**Range**
**PelvF unbranched**	1	1	(1–1)	1	(1–1)	1	(1–1)
**PelvF branched**	10	10	(9–11)	10	(9–10)	10	(10–11)
**Meristic characters**	*** C. brienzii***	**Lake Brienz**					
	**Holotype**	**Both sexes**					
		***N-total* = 13**		***N-females* = 4**		***N-males* = 9**	
		**Mode**	**Range**	**Mode**	**Range**	**Mode**	**Range**
**PecF unbranched**	1	1	(1–1)	1	(1–1)	1	(1–1)
**PecF branched**	15	15	(15–17)	15	(15–17)	15	(15–17)
**DF unbranched**	4	4	(3–4)	4	(4–4)	4	(3–4)
**DF branched**	12	11	(10–13)	11	(10–12)	10	(10–13)
**AF unbranched**	4	4	(3–4)	4	(4–4)	4	(3–4)
**AF branched**	13	12	(11–13)	13	(11–13)	12	(12–12)
**LS**	89	86	(80–91)	89	(80–91)	86	(80–88)
**PDS**	36	35	(32–40)	na	(34–37)	32	(32–40)
**TDS**	9	9	(7–10)	9	(7–9)	9	(8–10)
**TAS**	8	8	(7–8)	8	(7–8)	8	(7–8)
**TPS**	8	8	(8–9)	8	(8–8)	8	(8–9)
**UGR**	14	14	(11–14)	13	(13–14)	12	(11–14)
**LGR**	25	24	(20–25)	24	(24–25)	23	(20–25)
**total GR**	39	37	(32–39)	37	(37–39)	32	(32–38)

**Table 8.** Morphological and meristic data of *C. profundus* from Lake Thun, NMBE-1077208, male, holotype; paratypes N = 27. For ranges of males and for both sexes, the total range and mean include the holotype.

**Morphological characters**	*** C. profundus***	**Lake Thun**					
	**Holotype**	**Both sexes**					
		***N-total* = 28**		***N-females = 6***		***N-males* = 22**	
		**Mean ± Stdev**	**Range**	**Mean ± Stdev**	**Range**	**Mean ± Stdev**	**Range**
**SL (mm)**	194.0	223.3±26.7	(188–316)	248.7±42.2	(188–316)	216.3±16	(188–241)
**Percentage of standard length**							
**PelvFB**	4.4	4.2±0.3	(3.6–5.0)	4.2±0.2	(4–4.5)	4.2±0.4	(3.6–5)
**PelvFS**	7.2	6.0±0.8	(4.0–7.2)	5.7±1	(4–6.8)	6.1±0.7	(4.8–7.2)
**PelvF**	16.9	17.7±1.1	(15.1–19.6)	17.3±0.9	(16.5–18.9)	17.9±1.1	(15.1–19.6)
**PecFB**	3.5	3.7±0.2	(3.2–4.3)	3.6±0.2	(3.2–3.8)	3.7±0.2	(3.4–4.3)
**PecF1**	16.9	18.4±1.1	(16.6–21.0)	18.1±1.3	(16.6–19.8)	18.5±1	(16.8–21)
**PecF2**	17.8	20.2±1.3	(17.7–23.2)	19.9±1.5	(17.7–22.1)	20.2±1.3	(17.8–23.2)
**DFB**	12.6	12.5±0.9	(10.5–14.5)	12.3±0.7	(11.3–13.4)	12.5±1	(10.5–14.5)
**DFAe**	18.7	19.5±1.4	(15.9–21.9)	18.7±1.9	(15.9–21.6)	19.7±1.2	(17–21.9)
**DFAd**	20.6	20.7±1.3	(17.5–23.2)	19.9±1.4	(17.5–21.5)	20.9±1.2	(18.3–23.2)
**DFPe**	5.1	5.0±0.5	(3.9–6.1)	5.1±0.4	(4.5–5.6)	5±0.6	(3.9–6.1)
**AFB**	13.5	13.2±1.0	(10.8–15.3)	13.4±0.8	(12.1–14.4)	13.1±1.1	(10.8–15.3)
**AFAe**	13.6	13.3±1.0	(10.9–14.7)	12.8±1	(10.9–13.9)	13.4±0.9	(11.3–14.7)
**AdFB**	5.1	5.3±0.6	(3.8–6.3)	5.3±0.4	(4.6–5.8)	5.2±0.6	(3.8–6.3)
**CF**	24.1	24.5±1.4	(21.8–27.8)	24.3±2	(21.8–27.8)	24.6±1.3	(22.2–27.8)
**CD**	7.5	7.3±0.3	(6.5–7.9)	7.5±0.2	(7.2–7.8)	7.3±0.3	(6.5–7.9)
**CL**	12.5	11.8±0.7	(10.2–13.0)	12±0.8	(10.9–13)	11.8±0.7	(10.2–13)
**PAdC**	16.9	18.3±1.1	(15.8–20.1)	18.5±0.9	(17.1–19.6)	18.2±1.1	(15.8–20.1)
**DHL**	16.4	16.4±0.6	(15.5–18.4)	16.2±0.5	(15.5–16.7)	16.5±0.6	(15.7–18.4)
**PreP**	55.2	54.2±1.5	(51.2–58.1)	53.3±1.2	(51.2–54.1)	54.5±1.4	(52.1–58.1)
**PreA**	79.2	78.4±1.4	(75.0–80.6)	77.8±1.3	(75.8–79.4)	78.6±1.4	(75–80.6)
**PreD**	48.5	48.3±1.3	(45.8–51.1)	47.8±1.8	(45.8–50)	48.5±1.2	(46.9–51.1)
**BD**	24.4	24.2±1.4	(22.1–27.6)	25.4±1.3	(24–27.6)	23.9±1.2	(22.1–26.6)
**PostD**	40.6	42.5±1.5	(38.9–44.5)	43.2±1.4	(41.3–44.5)	42.3±1.5	(38.9–44.4)
**TL**	122.2	121.3±1.7	(117.3–125.6)	120.5±1.1	(118.9–121.8)	121.5±1.8	(117.3–125.6)
**HL (mm)**	41.2	48.9±5.5	(39.8–66.2)	54.1±8.7	(39.8–66.2)	47.4±3.2	(41.2–53.7)
**Percentage of head length**							
**SN**	23.6	23.5±0.8	(21.8–24.8)	23.3±0.6	(22.5–24)	23.6±0.8	(21.8–24.8)
**ED**	23.3	23.8±1.4	(21.3–26.2)	23.7±1.5	(21.9–25.7)	23.8±1.4	(21.3–26.2)
**EC**	30.9	29.2±1.4	(26.2–32.1)	28.2±1.6	(26.2–31.1)	29.5±1.3	(26.9–32.1)
**EH**	24.5	23.6±0.9	(21.8–25.5)	23.2±0.7	(21.9–23.9)	23.7±0.9	(21.8–25.5)
**ES**	5.7	4.6±0.8	(3.0–5.9)	4.3±0.9	(3.5–5.9)	4.7±0.7	(3–5.7)
**PostO**	51.1	50.9±1.4	(48–54)	52.2±1.8	(49.2–54)	50.6±1	(48–52.1)
**HD**	78.3	71.8±2.8	(65.9–78.3)	73±2.1	(69.4–75.7)	71.5±2.9	(65.9–78.3)
**MW**	11.2	10±0.8	(8.5–11.7)	10±0.5	(9.4–10.7)	10±0.9	(8.5–11.7)
**UJ**	29.1	28.7±1.2	(26.4–30.6)	28.1±1.3	(26.4–30)	28.9±1.1	(26.8–30.6)
**LJ**	41.4	39.9±1.7	(37–43.6)	39.1±1.4	(37–40.9)	40.1±1.8	(37.2–43.6)
**M**	24	20.7±1.2	(17.3–24)	20.1±1.4	(17.3–21.2)	20.8±1.1	(18.7–24)
**SD**	10.1	10±0.8	(8.1–11.3)	9.7±0.6	(8.8–10.7)	10±0.8	(8.1–11.3)
**SW**	17.6	15.8±1.3	(12.5–17.8)	15.3±1.6	(13.7–17.3)	16±1.1	(12.5–17.8)
**HW**	57.3	52.4±3.3	(46.7–58.6)	53.1±3.9	(46.7–58.6)	52.2±3.1	(47.4–57.7)
**IOW**	28.7	28.1±1.2	(26.1–30.3)	28.9±1.4	(26.5–30.3)	27.9±1.1	(26.1–29.5)
**INW**	11.1	11.1±1	(8.2–13.3)	11.7±1.1	(10.3–13.3)	10.9±1	(8.2–12.5)
**LJW**	9.3	11.7±2.2	(7.8–16.2)	12.7±0.7	(11.4–13.6)	11.5±2.4	(7.8–16.2)
**UJW**	28.9	26±1.7	(22.7–29.2)	25.2±1.5	(22.7–27.4)	26.2±1.7	(22.8–29.2)
**MGR**	10	9.2±1.1	(7.6–11.7)	9.4±1.2	(7.6–10.9)	9.2±1.1	(8–11.7)
**LGR**	10.7	10.1±1.2	(7.8–12.4)	10.5±1.6	(7.8–12.4)	9.9±1.1	(8.1–12.3)
**UA**	19.6	18±1.8	(15.5–21.8)	18.7±2.4	(15.5–21.8)	17.8±1.6	(15.5–21.2)
**LA**	35.8	34.3±1.8	(30.3–37.7)	35.1±2.1	(32.9–37.7)	34.1±1.7	(30.3–36.6)
**Meristic characters**		**Mode**	**Range**				
**PelvF unbranched**	1	1	(1–1)	1	(1–1)	1	(1–1)
**PelvF branched**	10	10	(9–11)	10	(10–11)	10	(9–11)
**PecF unbranched**	1	1	(1–1)	1	(1–1)	1	(1–1)
**Meristic characters**	*** C. profundus***	**Lake Thun**					
	**Holotype**	**Both sexes**					
		***N-total* = 28**		***N-females = 6***		***N-males* = 22**	
		**Mode**	**Range**	**Mode**	**Range**	**Mode**	**Range**
**PecF branched**	16	16	(13–17)	16	(16–16)	16	(13–17)
**DF unbranched**	5	4	(3–5)	4	(3–4)	4	(3–5)
**DF branched**	10	10	(9–12)	11	(10–12)	10	(9–11)
**AF unbranched**	5	3	(2–5)	2	(2–4)	3	(2–5)
**AF branched**	11	12	(11–14)	12	(12–14)	12	(11–13)
**LS**	83	84	(76–90)	83	(80–89)	84	(76–90)
**PDS**	34	34	(32–38)	32	(32–37)	34	(32–38)
**TDS**	9	9	(8–10)	9	(8–10)	9	(8–10)
**TAS**	8	8	(6–8)	8	(8–8)	8	(6–8)
**TPS**	8	8	(7–9)	9	(8–9)	8	(7–9)
**UGR**	8	9	(5–10)	7	(6–10)	9	(5–9)
**LGR**	13	14	(10–18)	17	(11–18)	14	(10–18)
**total GR**	21	21	(15–27)	na	(18–27)	21	(15–26)

**Table 9.** Morphological and meristic data of *C. acrinasus* from Lake Thun, NMBE-1077271, male, holotype; paratypes N = 25. For males and for both sexes, the range and mean include the holotype.

**Morphological characters**	*** C. acrinasus***	**Lake Thun**					
	**Holotype**	**Both sexes**					
		***N-total* = 26**		***N-females* = 4**		***N-males* = 22**	
		**Mean±Stdev**	**Range**	**Mean±Stdev**	**Range**	**Mean±Stdev**	**Range**
**SL (mm)**	239.5	237.3±21.2	(197–278)	235.5±26.5	(197–254)	237.6±20.8	(197–278)
**Percentage of standard length**							
**PelvFB**	5.1	4.1±0.4	(3.5–5.1)	3.7±0.4	(3.5–4.3)	4.1±0.4	(3.5–5.1)
**PelvFS**	7	6.2±0.7	(4.6–7.5)	6.2±0.3	(5.7–6.4)	6.2±0.7	(4.6–7.5)
**PelvF**	17.4	16±0.9	(14.3–17.5)	15.6±1.2	(14.6–17.2)	16.1±0.8	(14.3–17.5)
**PecFB**	3.6	3.4±0.2	(3.1–4.0)	3.2±0.1	(3.1–3.4)	3.5±0.2	(3.1–4)
**PecF1**	17.4	15.9±1.1	(13.8–18.2)	15.6±1.8	(14.1–18.1)	16±1	(13.8–18.2)
**PecF2**	18.6	16.9±1.3	(15–19.7)	16.5±2.2	(15–19.7)	17±1.1	(15–19)
**DFB**	14.8	12.4±0.8	(11.2–14.8)	12.3±0.8	(11.5–13.4)	12.4±0.9	(11.2–14.8)
**DFAe**	20.9	18.1±1.2	(15.7–20.9)	17.8±1.5	(15.8–19.1)	18.1±1.2	(15.7–20.9)
**DFAd**	21.7	19.3±1.1	(17.0–21.7)	19.1±1.1	(18.0–20.3)	19.3±1.2	(17–21.7)
**DFPe**	5.5	5.0±0.5	(4.0–6.1)	4.9±0.5	(4.2–5.3)	5±0.5	(4–6.1)
**AFB**	13.6	12.6±0.6	(11.3–13.6)	12.6±0.6	(11.9–13.3)	12.6±0.6	(11.3–13.6)
**AFAe**	13	11.6±0.8	(9.2–13.0)	11.4±0.5	(11.0–12.2)	11.7±0.9	(9.2–13)
**AdFB**	4.5	4.7±0.7	(3.7–6.2)	4.8±0.6	(4.2–5.6)	4.7±0.7	(3.7–6.2)
**CF**	24	23.3±0.9	(21.5–25.1)	23.2±0.2	(23–23.4)	23.4±1	(21.5–25.1)
**CD**	7.5	7.6±0.4	(7.1–8.3)	7.8±0.3	(7.6–8.2)	7.6±0.4	(7.1–8.3)
**CL**	11.7	12.8±0.6	(11.7–14.2)	12.4±0.4	(11.9–12.8)	12.9±0.6	(11.7–14.2)
**PAdC**	15	18.1±1.2	(15–20.1)	17.6±1	(16.2–18.4)	18.2±1.2	(15–20.1)
**DHL**	14.9	15.2±0.6	(13.8–16.1)	14.9±0.9	(14.0–15.9)	15.2±0.5	(13.8–16.1)
**PreP**	50.3	52.6±1.6	(49.1–56.8)	51.9±0.5	(51.4–52.4)	52.7±1.8	(49.1–56.8)
**PreA**	78.5	77.7±1.2	(75.3–80.3)	77.1±0.5	(76.5–77.6)	77.8±1.3	(75.3–80.3)
**PreD**	45.4	47.5±1.4	(45–50.7)	47.5±1.1	(46.3–48.6)	47.5±1.4	(45–50.7)
**BD**	25.6	24.7±1.6	(20.7–28.1)	26.1±1.6	(24.4–28.1)	24.4±1.5	(20.7–26.7)
**PostD**	41.2	43±1.3	(40.3–45.6)	42.2±1.6	(41–44.3)	43.1±1.3	(40.3–45.6)
**TL**	123.2	120.6±1.7	(116–123.2)	119±2.5	(116–121.5)	120.8±1.4	(118.2–123.2)
**HL (mm)**	49	49.9±4	(41.5–58.4)	48.5±4.7	(41.5–51.3)	50.1±3.9	(41.5–58.4)
**Percentage of head length**							
**SN**	23.4	23.9±1.4	(20.5–27)	22.6±1.8	(20.5–24.6)	24.1±1.3	(21.6–27)
**ED**	23.2	23.7±0.8	(21.6–25.5)	23.8±0.8	(22.6–24.4)	23.7±0.9	(21.6–25.5)
**EC**	27.4	27.7±1	(26–29.6)	28.6±1	(27.2–29.6)	27.6±0.9	(26–28.8)
**EH**	22.8	22.9±0.9	(21.7–24.8)	23.6±1.1	(22.2–24.8)	22.8±0.8	(21.7–24.5)
**ES**	4.9	4.7±0.8	(3.2–6.4)	5.6±0.9	(4.8–6.4)	4.5±0.6	(3.2–6.1)
**PostO**	51	50.9±1.5	(48.5–54.1)	52±1.8	(49.8–54.1)	50.7±1.4	(48.5–53)
**HD**	69.8	69.1±2.4	(65.1–74.9)	69.8±2	(67.8–72.5)	68.9±2.5	(65.1–74.9)
**MW**	9.7	9.8±0.7	(8.1–11.4)	9.8±0.6	(8.8–10.3)	9.8±0.8	(8.1–11.4)
**UJ**	28.8	29.4±1.2	(26.7–30.9)	30.1±0.8	(29.3–30.8)	29.2±1.3	(26.7–30.9)
**LJ**	40.5	40.9±1.7	(38.6–47)	40.5±1.1	(39–41.5)	41±1.8	(38.6–47)
**M**	21.1	21.8±1	(19.4–23.8)	21.9±0.9	(21.3–23.2)	21.8±1	(19.4–23.8)
**SD**	9.8	8.6±1.3	(6–11.3)	9±1.1	(7.9–10.5)	8.6±1.3	(6–11.3)
**SW**	17.1	16±1.5	(13.1–18.1)	16.2±1.3	(14.7–17.6)	15.9±1.5	(13.1–18.1)
**HW**	53.8	49.6±3.2	(43.9–56.2)	51.1±2.4	(48.2–53.8)	49.4±3.3	(43.9–56.2)
**IOW**	25.8	27±2.1	(21.3–31.5)	27.1±1.4	(25.1–28.1)	27±2.3	(21.3–31.5)
**INW**	10.8	11.7±1	(9.5–13.4)	11.7±1.2	(10.5–13.3)	11.7±1	(9.5–13.4)
**LJW**	12.4	12.2±1.2	(9.2–14.3)	12.5±1	(11–13.4)	12.1±1.2	(9.2–14.3)
**UJW**	24	22.8±2.1	(18.2–27.5)	23.7±2.7	(20.4–26.3)	22.6±2	(18.2–27.5)
**MGR**	13.1	13.4±1.6	(9.1–16.6)	14.4±1.9	(11.9–16.6)	13.2±1.6	(9.1–15.1)
**LGR**	15	14.5±1.4	(11.4–16.9)	15.7±1.2	(14.4–16.9)	14.3±1.3	(11.4–16.3)
**UA**	19.7	18.1±1.6	(13.5–20.3)	18.5±1.6	(16.2–19.8)	18.1±1.6	(13.5–20.3)
**LA**	36.7	34.9±1.7	(32.3–38.9)	35.9±2.1	(34–38.9)	34.8±1.7	(32.3–38.4)
**Meristic characters**		**Mode**	**Range**	**Mode**	**Range**	**Mode**	**Range**
**PelvF unbranched**	1	1	(1–1)	1	(1–1)	1	(1–1)
**PelvF branched**	11	10	(9–12)	10	(10–11)	11	(9–12)
**PecF unbranched**	1	1	(1–1)	1	(1–1)	1	(1–1)
**PecF branched**	15	15	(13–16)	16	(15–16)	15	(13–16)
**Meristic characters**	*** C. acrinasus***	**Lake Thun**					
	**Holotype**	**Both sexes**					
		***N-total* = 26**		***N-females* = 4**		***N-males* = 22**	
		**Mode**	**Range**	**Mode**	**Range**	**Mode**	**Range**
**DF unbranched**	3	4	(3–4)	3	(3–4)	4	(3–4)
**DF branched**	12	10	(9–12)	11	(10–12)	10	(9–12)
**AF unbranched**	3	3	(2–4)	3	(3–3)	3	(2–4)
**AF branched**	13	11	(11–13)	12	(11–13)	11	(11–13)
**LS**	80	84	(79–88)	85	(84–85)	80	(79–88)
**PDS**	34	34	(33–42)	34	(34–41)	35	(33–42)
**TDS**	10	10	(9–10)	10	(9–10)	10	(9–10)
**TAS**	8	8	(8–9)	8	(8–8)	8	(8–9)
**TPS**	9	8	(8–9)	9	(8–9)	8	(8–9)
**UGR**	13	13	(10–15)	na	(10–15)	13	(10–14)
**LGR**	20	24	(20–26)	24	(21–24)	24	(20–26)
**total GR**	33	36	(30–40)	35	(34–36)	36	(30–40)

**Figure 11.**
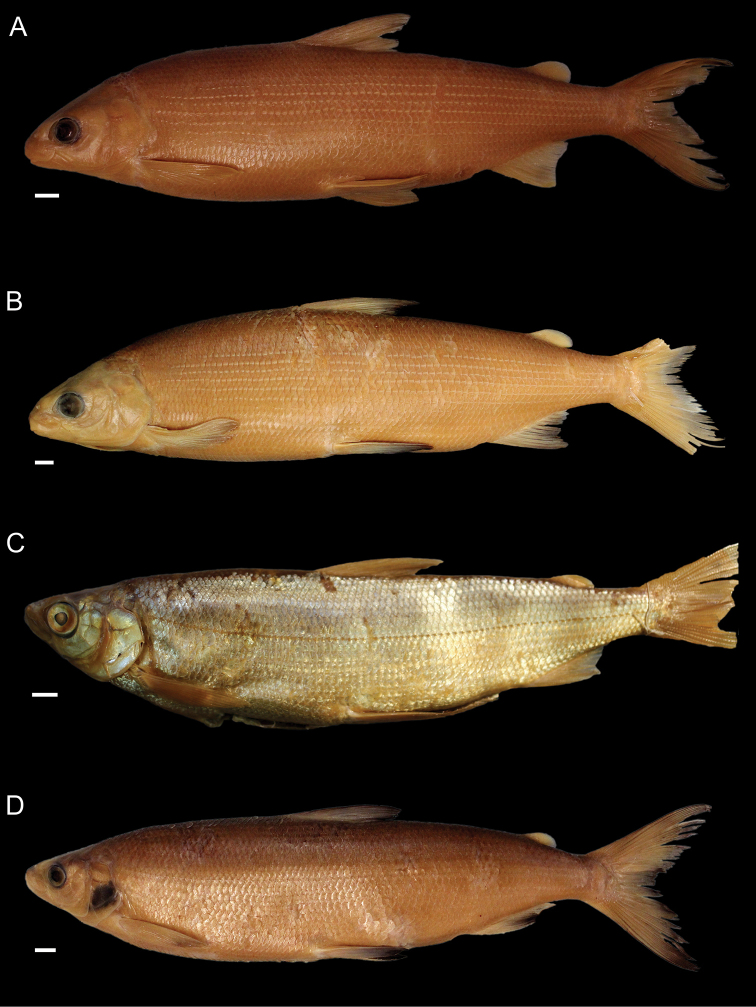
Types of the Lake Constance species, Switzerland **A***Coregonus gutturosus*, non-type, NMBE-1076232 (Eawag-248–1), 250 mm, sex unknown, preserved specimen **B***Coregonus arenicolus*, holotype, 296 mm, NMBE-1076223 (Eawag-239–1), sex unknown, preserved specimen **C***Coregonus macrophthalmus*, syntype, MHNG-716.052, 215 mm, sex unknown, preserved specimen **D***Coregonus wartmanni*, non-type, NMBE-1076206, 301 mm, female, preserved specimen. The white scale (1cm) below each fish acts as a reference for the actual size of the specimen.

**Table 10.** The first- and second-best ratios retrieved from the LDA ratio extractor of either head or body characters (see Table 1) alone or combined, used for pair-wise comparisons of all contemporary specimens from the six whitefish species of Lake Thun. For some comparisons only a subset of characters could be used (a-d); the respective characters that were excluded are listed at the end of the table. Only external characters were used for the LDA comparisons, since internal characters (gill raker and gill arch length) cannot be measured on live specimens, and are thus not informative to assign specimens to species in the field. Additionally, species were combined to find first- and second-best ratios that distinguish one species or a combination of species vs. all other species. For multi-species comparisons, only the comparisons that yielded distinguishing ratios are shown. δ is a measure of how good shape discriminates in comparison to size (i.e., the smaller δ the less allometry). Ratios marked with an asterisk * have very little (for the pairwise species comparisons not more than one specimen of one species overlaps with the other species) or no overlap and were thus eligible for use in the species key and the diagnoses.

Characters	Species comparison	Best ratios	Range species 1	Range species 2	Standard distance	δ (Shape vs. size)
head + body	* C. albellus* vs. *C. alpinus*	1: CD/UJ *	0.96–1.29	1.36–1.65	18.09	0.1
		2: AdFB/ES	5.54–13.54	2.87–5.94	17.49	0.1
body	* C. albellus* vs. *C. alpinus*	1: CD/DHL *	0.44–0.54	0.54–0.62	5.98	0.26
		2: DFB/AdFB	1.6–2.66	2.31–4.02	4.86	0.31
head	* C. albellus* vs. *C. alpinus*	1: UJ/ES	6.48–16.01	4.52–7.93	6	0.22
		2: HL/UJ	2.88–3.47	3.33–4.11	5.26	0.25
head + body	* C. albellus* vs. *C. fatioi*	1: TL/EH	21.93–27.57	21.91–29.46	3.07	0.08
		2: PelvF/PecF1	0.87–1.03	0.87–1.07	2.89	0.08
body	* C. albellus* vs. *C. fatioi*	1: PecF1/TL	0.12–0.16	0.11–0.15	1.4	0.19
		2: DFAe/DFAd	0.88–1	0.9–0.98	1.18	0.21
head	* C. albellus* vs. *C. fatioi*	1: EH/HL	0.21–0.26	0.2–0.25	1.63	0.08
		2: LJW/ES	2.14–7.79	1.59–8.18	1.35	0.1
head + body	* C. albellus* vs. *C. steinmanni*	1: CD/UJ *	0.96–1.29	1.36–1.55	13.8	0.12
		2: AdFB/ES	5.54–13.54	3.31–6.31	13	0.12
head	* C. albellus* vs. *C. steinmanni*	1: HL/UJ	2.88–3.47	3.33–3.97	5.3	0.21
		2: LJ/ES	8.25–20.33	6.65–12.45	4.59	0.23
head + body	* C. albellus* vs. *C. profundus*	1: CL/EC *	1.97–2.87	1.56–2.09	13.19	0.03
		2: DHL/M	2.77–3.53	3.21–3.79	12.79	0.03
body	* C. albellus* vs. *C. profundus*	1: CL/DHL	0.75–1.04	0.61–0.82	4.43	0.06
		2: CD/BD	0.26–0.31	0.28–0.34	3.38	0.07
head	* C. albellus* vs. *C. profundus*	1: EC/UJ	0.74–0.95	0.87–1.05	5.02	0.1
		2: SW/ES	3.22–9.67	2.31–5.26	4.05	0.12
head + body	* C. albellus* vs. *C. acrinasus*	1: AdFB/ES	5.54–13.54	3.31–6.5	9.13	0.06
		2: CD/UJW	1.14–1.79	1.4–2	8.69	0.07
body	* C. albellus* vs. *C. acrinasus*	1: PecF1/CD	2.13–2.76	1.8–2.39	4.5	0.11
		2: DFB/AdFB	1.6–2.66	1.98–3.45	3.88	0.13
head	* C. albellus* vs. *C. acrinasus*	1: UJW/ES	5.27–13.65	3.22–7.96	4.19	0.14
		2: ED/UJ	0.66–0.84	0.74–0.9	3.51	0.16
**Characters**	**Species comparison**	**Best ratios**	**Range species 1**	**Range species 2**	**Standard distance**	**δ (shape vs. Size)**
head + body	* C. alpinus* vs. *C. fatioi*	1: CD/PostD *	0.17–0.21	0.14–0.17	22.73	0.07
		2: DFAe/UJ	3.14–3.93	2.43–3.41	22.33	0.07
body	* C. alpinus* vs. *C. fatioi*	1: CD/PostD *	0.17–0.21	0.14–0.17	8.98	0.17
		2: DFAe/DHL	1.26–1.55	1.02–1.36	7.9	0.19
head	* C. alpinus* vs. *C. fatioi*	1: HD/UJ	2.34–2.9	2.13–2.57	3.86	0.3
		2: MW/ES	1.47–3	1.82–6.16	3.15	0.34
head + body	* C. alpinus* vs. *C. steinmanni* (a)	ED/EC	0.74–0.9	0.74–0.9	8.07	0.05
		CD/CL	0.6–0.75	0.54–0.7	8.02	0.05
body	* C. alpinus* vs. *C. steinmanni*	1: DFAe/AFAe	1.5–1.83	1.43–1.62	5.7	0.06
		2: PelvFS/DFAe	0.24–0.36	0.29–0.37	5.58	0.07
head	* C. alpinus* vs. *C. steinmanni*	1: EC/SW	1.47–2.13	1.43–1.7	2.45	0.16
		2: ED/EC	0.74--0.9	0.79–0.9	2.2	0.18
head + body	* C. alpinus* vs. *C. profundus*	1: CD/DHL *	0.54–0.62	0.4–0.49	19.86	0.07
		2: PecF2/CF	0.63–0.82	0.74–0.90	19.01	0.07
body	* C. alpinus* vs. *C. profundus*	1: CD/DHL *	0.54–0.62	0.4–0.49	9.31	0.15
		2: PecF2/CF	0.63–0.82	0.74–0.90	7.32	0.19
head	* C. alpinus* vs. *C. profundus*	1: EH/PostD	0.09–0.11	0.11–0.15	4.32	0.21
		2: SD/UJW	0.35–0.51	0.30–0.44	3.93	0.23
head + body	* C. alpinus* vs. *C. acrinasus*	1: CD/LJ	0.95–1.11	0.79–1	65.21	0.02
		2: CF/M *	5.55–6.55	4.4–5.57	65.13	0.02
body	* C. alpinus* vs. *C. acrinasus*	1: CD/DHL	0.54–0.62	0.46–0.58	4.69	0.25
		2: DFAe/DFPe	3.39–4.72	2.84–4.54	3.91	0.29
head	* C. alpinus* vs. *C. acrinasus*	1: PostO/M	2.4–3	2.17–2.56	4.26	0.21
		2: HD/MW	6.57–8.7	6.02–8.87	3.65	0.24
**Characters**	**Species comparison**	**Best ratios**	**Range species 1**	**Range species 2**	**Standard distance**	**δ (shape vs. Size)**
head + body	* C. fatioi* vs. *C. steinmanni* (b)	1: CD/UJ *	1.02–1.34	1.36–1.55	33.96	0.04
		2: PelvF/PAdC	0.73–1	0.84–0.1	33.71	0.04
body	* C. fatioi* vs. *C. steinmanni*	1: CD/PostD *	0.14–0.17	0.17–0.20	6.34	0.22
		2: DHL/BD	0.5–0.7	0.45–0.58	5.37	0.25
head	* C. fatioi* vs. *C. steinmanni*	1: HD/UJ	2.13–2.57	2.42–2.83	4.41	0.23
		2: HW/LJW	3.17–6.12	3.72–5.1	3.25	0.29
head + body	* C. fatioi* vs. *C. profundus*	1: CL/EC	1.84–2.98	1.56–2.09	10.03	0.02
		2: DHL/UJ	2.11–2.70	2.32–2.92	9.54	0.02
body	* C. fatioi* vs. *C. profundus*	1: CL/DHL	0.76–1.04	0.61–0.82	4.44	<0.01
		2: DFPe/CD	0.56–0.87	0.56.0.82	3.2	<0.01
head	* C. fatioi* vs. *C. profundus*	1: EC/SW	1.32–1.73	1.63–2.38	5.05	0.08
		2: UJ/UJW	1.04–1.50	1–1.29	4.28	0.09
head + body	* C. fatioi* vs. *C. acrinasus*	1: CD/PostD	0.14–0.17	0.16–0.2	8.3	0.05
		2: ED/SW	1.08–1.5	1.3–1.79	8	0.05
body	* C. fatioi* vs. *C. acrinasus*	1: CD/PostD	0.14–0.17	0.16–0.2	3.66	0.07
		2: AFAe/DHL	0.69–0.9	0.61–0.93	2.93	0.09
head	* C. fatioi* vs. *C. acrinasus*	1: ED/SW	1.08–1.5	1.3–1.79	3.05	0.15
		2: MW/ES	1.82–6.16	1.4–3.02	2.45	0.18
**Characters**	**Species comparison**	**Best ratios**	**Range species 1**	**Range species 2**	**Standard distance**	**δ (shape vs. Size)**
head + body	* C. steinmanni* vs. *C. profundus* (c)	1: CD/DHL *	0.53–0.63	0.4–0.49	23.9	0.05
		2: CL/IOW	2.05–2.69	1.69–2.28	23.3	0.05
body	* C. steinmanni* vs. *C. profundus*	1: CD/DHL *	0.53–0.63	0.4–0.49	9.13	0.14
		2: PecF2/DFAe	0.76–0.96	0.82–1.21	7.44	0.17
head	* C. steinmanni* vs. *C. profundus*	1: SW/UJW	0.65–0.80	0.54–0.69	5.9	0.12
		2: EH/PostO	0.36–0.47	0.41–0.52	5.37	0.13
head + body	* C. steinmanni* vs. *C. acrinasus* (d)	1: CD/M *	1.86–2.24	1.4–1.9	160.64	<0.01
		2: PostD/LJ	4.96–5.9	4.65–5.43	160.6	<0.01
body	* C. steinmanni* vs. *C. acrinasus*	1: CD/DHL	0.53–0.63	0.46–0.58	4.46	0.23
		2: PelvF/DHL	1.08–1.26	0.95–1.16	3.83	0.26
head	* C. steinmanni* vs. *C. acrinasus*	1: ED/HD	0.29–0.33	0.31–0.37	4.54	0.13
		2: HL/M	4.6–5.53	4.21–5.17	3.41	0.17
**Characters**	**Species comparison**	**Best ratios**	**Range species 1**	**Range species 2**	**Standard distance**	**δ (shape vs. Size)**
head + body	* C. profundus* vs. *C. acrinasus*	1: PecF2/CD	2.37–3.16	1.91–2.59	13.46	0.01
		2: LJ/UJW	1.34–1.86	1.54–2.27	13.12	0.01
body	* C. profundus* vs. *C. acrinasus*	1: PecF2/CD	2.37–3.16	1.91–2.59	4.58	0.05
		2: DHL/TL	0.13–0.15	0.12–0.13	3.48	0.06
head	* C. profundus* vs. *C. acrinasus*	1: M/UJW	0.69–0.94	0.8–1.22	4.45	0.02
		2: EC/LJ	0.66–0.81	0.58–0.72	3.88	0.02
**Characters**	**Mulitple species comparison**	**Best ratios**	**Range group 1**	**Range group 2**	**Standard distance**	**δ (shape vs. Size)**
head + body	* C. alpinus* + *C. steinmanni* vs. 4 other species	1: CD/UJ *	1.36–1.65	0.96–1.43	5.34	0.24

(a) PelvFS, PecF1, DFAd, DFAe, DFPe, TL, SL, EH, SD, SW, INW, IOW (b) PelvFS, PecF1, DFAd, TL (c) PelvFS, PecF1, DFAd, TL,EH (d) PelvFS, PecF1, DFAd, TL, EH, ES, EC

**Table 11.** The first- and second-best ratios retrieved from the LDA ratio extractor of either head or body characters (see Table 1) alone or combined, used for pair-wise comparisons of all contemporary specimens from the four whitefish species of Lake Brienz. For some species comparisons only a subset of characters could be used (a-l); the respective characters that were excluded are listed at the end of the table. Only external characters were used for the LDA comparisons, since internal characters (gill raker and gill arch length) cannot be measured on live specimens, and are thus not informative to assign specimens to species in the field. Due to large size differences between the species the LDA ratios were calculated with three different datasets; once each with individuals larger or smaller than 163.5mm standard length and once with the full size ranges of all species. For the multi-species comparisons, only the comparisons that yielded distinguishing ratios are shown. δ is a measure of how good shape discriminates in comparison to size (i.e., the smaller the less allometry). Ratios marked with an asterisk * have very little (for the pairwise species comparisons not more than one specimen of one species overlaps with that of the other species) or no overlap and were thus eligible for use in the species key and the diagnoses.

Characters	Species comparison	Size range	Best ratios	Range species 1	Range species 2	Standard distance	δ (Shape vs. size)
head + body	* C. albellus vs. C. alpinus (a)*	<163.5mm	1: PreA/LJ *	6.33-7.44	9.24-9.97	27.13	0.04
			2: AFAe/M	1.65-2.25	2.58-2.63	25.94	0.04
body	* C. albellus vs. C. alpinus*	<163.5mm	1: PecF2/DFAd *	0.81-1.06	0.78-0.8	9.97	0.14
			2: DHL/PreD	0.34-0.42	0.32-0.34	9.4	0.15
head	* C. albellus vs. C. alpinus*	<163.5mm	1: HD/LJ	1.30-1.55	1.77-1.92	15.43	0.02
			2: IOW/UJW	0.89-1.30	1.20-1.26	14.14	0.02
head + body	* C. albellus vs. C. fatioi*	<163.5mm	1: PecF2/PreA *	0.22-0.28	0.2-0.22	5.78	0.16
			2: DHL/PreP	0.31-0.38	0.30-0.32	4.49	0.2
body	* C. albellus vs. C. fatioi*	<163.5mm	1: PecF2/PreA *	0.22-0.28	0.2-0.22	6.76	0.17
			2: DHL/TL	0.13-0.18	0.13-0.14	5.7	0.19
head	* C. albellus vs. C. fatioi*	<163.5mm	1: UJ/ES *	6.81-12.42	4.51-6.15	8.63	0.12
			2: EH/HL *	0.27-0.31	0.23-0.27	7.3	0.14
head + body	* C. albellus vs. C. brienzii (b)*	<163.5mm	1: PreD/LJ *	3.99-4.68	5.05-5.57	47.9	0.01
			2: M/ES *	5.35-9.76	3.31-4.37	47.63	0.01
body	* C. albellus vs. C. brienzii*	<163.5mm	1: PecF2/PreD *	0.36-0.45	0.29-0.32	15.95	0.06
			2: DHL/TL	0.13-0.18	0.13-0.14	9.91	0.05
head	* C. albellus vs. C. brienzii*	<163.5mm	1: LJ/ES *	9.62-17.28	6.01-6.49	12.51	0.05
			2: HL/UJ	2.87-3.5	3.19-3.6	8.87	0.04
**Characters**	**Species comparison**	**Size range**	**Best ratios**	**Range species 1**	**Range species 2**	**Standard distance**	**δ (Shape vs. size)**
head + body	* C. alpinus vs. C. fatioi (b)*	>163.5mm	1: AFAe/UJ *	1.96-2.5	1.66-1.96	26.08	0.04
			2: CL/PreA	0.14-0.18	0.17-0.21	26.46	0.04
body	* C. alpinus vs. C. fatioi*	>163.5mm	1: AFae/TL	0.1-0.11	0.09-0.1	13.41	0.11
			2: CL/PreA	0.14-0.18	0.17-0.21	13.41	0.11
head	* C. alpinus vs. C. fatioi*	>163.5mm	1: HL/UJ *	3.55-3.93	3.13-3.55	11.51	0.07
			2: LJW/UJW	0.33-0.44	0.38-0.55	11.02	0.07
head + body	* C. alpinus vs. C. brienzii (c)*	>163.5mm	1: CD/SW *	2.25-2.64	1.82-2.04	34.25	0.02
			2: LJW/UJW *	0.33-0.44	0.45-0.55	33.91	0.02
body	* C. alpinus vs. C. brienzii (d)*	>163.5mm	1: DFAe/PAdC *	1.11-1.32	0.96-1.16	18.53	0.07
			2: CD/AFB	0.61-0.68	0.52-0.62	18.31	0.07
head	* C. alpinus vs. C. brienzii (e)*	>163.5mm	1: LJW/UJW *	0.33-0.44	0.45-0.55	7.44	0.08
			2: PostO/UJ	1.8-2.12	1.57-1.86	6.78	0.08
**Characters**	**Species comparison**	**Size range**	**Best ratios**	**Range species 1**	**Range species 2**	**Standard distance**	**δ (Shape vs. size)**
head + body	* C. fatioi vs. C. brienzii (f)*	>163.5mm	1: CL/PAdC	0.71-0.86	0.66-0.76	7.46	0.08
			2: BD/LJ	2.44-3.05	2.31-2.82	7.36	0.08
body	* C. fatioi vs. C. brienzii*	>163.5mm	1: CL/PAdC	0.71-0.86	0.66-0.76	6.04	0.1
			2: CF/BD	0.87-1.13	0.93-1.12	5.92	0.1
head	* C. fatioi vs. C. brienzii*	>163.5mm	1: ED/M	1.03-1.19	1.04-1.57	3.58	0.18
			2: HW/UJW	1.89-2.23	1.88-2.33	3.38	0.19
**Characters**	**Species comparison**	**Size range**	**Best ratios**	**Range species 1**	**Range species 2**	**Standard distance**	**δ (Shape vs. size)**
head + body	* C. alpinus vs. other 3 species*	<163.5mm	1: DFAd/LJ *	2.57-2.58	1.6-2.1	23.47	0.03
			2: AdFB/PAdC	0.26-0.28	0.21-0.42	22.66	0.03
head + body	* C. albellus vs. other 3 species*	<163.5mm	1: PostD/EH *	5.47-6.93	7.5-8.9	48.36	0.02
			2: UJW/ES	4.88-9.3	3.41-5.31	48.13	0.02
head + body	* C. alpinus vs. C. fatioi + C. brienzii (g)*	>163.5mm	1: DFAe/UJ *	3.28-4.1	2.58-3.19	24.71	0.05
			2: CD/SW *	2.25-2.64	1.76-2.27	24.37	0.05
**Characters**	**Species comparison**	**Size range**	**Best ratios**	**Range species 1**	**Range species 2**	**Standard distance**	**δ (Shape vs. size)**
head + body	* C. albellus vs. C. alpinus (h)*	100-290	1: PreD/LJ *	3.99-4.68	5.6-6.81	22.86	0.13
			2: DFAe/UJ *	2.14-2.79	3.25-4.1	21.65	0.14
head	* C. albellus vs. C. alpinus*	100-290	1: HD/UJ *	1.87-2.2	2.38-2.78	14.39	0.18
			2: LJ/IOW *	1.53-1.99	1.33-1.57	13.25	0.19
head + body	* C. albellus vs. C. fatioi*	100-290	1: PreP/EH *	6.56-7.98	8.94-11.43	15.95	0.13
			2: CL/UJ	1.44-2.02	1.93-2.72	15.09	0.14
head + body	* C. albellus vs. C. brienzii (i)*	100-290	1: PreD/EH *	6.1-7.58	8.12-10.32	50.86	0.04
			2: CL/LJ	0.99-1.45	1.38-1.65	50.6	0.04
head	* C. albellus vs. C. brienzii*	100-290	1: EH/HL *	0.27-0.31	0.22-0.27	9.33	0.18
			2: LJ/ES	9.62-17.28	6.08-12.43	8.57	0.22
**Characters**	**Species comparison**	**Size range**	**Best ratios**	**Range species 1**	**Range species 2**	**Standard distance**	**δ (Shape vs. size)**
head + body	* C. alpinus vs. C. brienzii (j)*	100-290	1: DFAd/LJW *	9.84-14.82	6.05-8.91	20.72	0.03
			2: DHL/LJ *	1.84-2.22	1.63-1.82	20.47	0.02
body	* C. alpinus vs. C. brienzii (k)*	100-290	1: PecF2/DFAd *	0.74-0.85	0.85-1.03	87.52	<0.01
			2: CD/PostD	0.17-0.2	0.15-0.18	87.48	<0.01
head	* C. alpinus vs. C. brienzii*	100-290	1: HD/LJW *	6.72-9.39	5.23-6.66	11.94	0.04
			2: HL/LJ *	2.54-2.96	2.19-2.47	11.61	0.04
head + body	* C. alpinus vs. C. fatioi (h)*	100-290	1: DFAe/UJ *	3.25-4.1	2.45-3.17	18.98	0.03
			2: PecF2/AFAe	1.24-1.47	1.37-1.63	18.63	0.03
body	* C. alpinus vs. C. fatioi*	100-290	1: PecF2/DFAe	0.77-0.89	0.87-1.02	9.25	0.08
			2: AFAe/PostD	0.27-0.32	0.22-0.29	8.71	0.08
head	* C. alpinus vs. C. fatioi*	100-290	1: LJW/UJW	0.33-0.47	0.37-0.55	5.62	0.08
			2: HL/UJ	3.43-3.93	3.13-3.63	4.98	0.08
**Characters**	**Species comparison**	**Size range**	**Best ratios**	**Range species 1**	**Range species 2**	**Standard distance**	**δ (Shape vs. size)**
head + body	* C. fatioi vs. C. brienzii (l)*	100-290	1: AFB/BD	0.45-0.67	0.44-0.58	28.19	<0.01
			2: PreD/M	8.87-14.85	9.13-11.41	28.16	<0.01
body	* C. fatioi vs. C. brienzii*	100-290	1: AFB/BD	0.45-0.67	0.44-0.58	2.76	0.05
			2: PreP/PreA	0.58-0.65	0.57-0.64	2.51	0.05
head	* C. fatioi vs. C. brienzii*	100-290	1: ED/M	1.04-1.57	1.03-1.28	1.72	0.1
			2: SN/MW	2.09-2.63	1.78-2.87	1.52	0.1
**Characters**	**Species comparison**	**Size range**	**Best ratios**	**Range species 1**	**Range species 2**	**Standard distance**	**δ (Shape vs. size)**
head + body	* C. albellus vs. other 3 species*	100-290	1: PreD/EH *	6.1-7.58	8.12-10.5	10.89	0.16
			2: CL/UJ	1.44-2.02	1.85-2.72	9.79	0.17
head + body	* C. alpinus vs. other 3 species*	100-290	1: DFAe/UJ *	3.25-4.1	2.14-3.19	9.59	0.11
			2: LJW/UJW	0.33-0.47	0.34-0.55	8.98	0.12

(a) PelvS, PecF1, DFAd, DFAe, DFPe, TL, SL, EH, SD, SW, INW, IOW (b) PelvFS, PelvFB, PecFB, DFPe, TL, EH, ED, SD, IOW (c) PelvFB, PelvFS, PelvF, PecF1, DFB, DFAe, DFPe, AFB, AFAe, AdFB, CF, PAdC, PreP, PreA, SL, TL, BD, PostD, DHL, ED, EH, ES, PostO, HD, MW, SN, SD, SW, IOW, INW (d) PecFB, PelvFB, PelvF, PelvFS, PecF1, DFAe, DFPe, AFAe, AdFB, PreP, CF, TL, PostD (e) ED, EH, HD, SD, SW, INW (f) PelvFB, PelvFS, PelvF, PecFB, DFAe, DFAd, DFPe, CF, PreP, SL, TL, ED, EH, MW, SD, SW, IOW, INW, ES (g) PelvFS, PecF1, PecFB, DFAd, DFPe, SL, TL, ED, EH, INW, CF (h) PelvS, PecF1, DFAd, DFAe, DFPe, TL, SL, EH, SD, SW, INW, IOW (i) PelvFS (j) PelvFB, PelvFS, PelvF, PecF1,DFAe, DFPe, AFB, AFAe,CF, SL, TL, BD, ED, EH, ES, PostO, HD, MW, SN, SD, SW, IOW, INW (k) PelvFS, PecF1, CF, SL, TL (l) PelvS, TL, INW

**Table 12.** Morphological and meristic data of *C. gutturosus* Gmelin, 1818, *C. arenicolus* Kottelat, 1997, *C. macrophthalmus* Nüsslin, 1882 and *C. wartmanni* Bloch, 1784 from Lake Constance. *Coregonus gutturosus* Gmelin, 1818, non-types N = 10. *Coregonus arenicolus* Kottelat, 1997, holotype, NMBE-1076223 (Eawag-239–1), sex unknown; paratypes N = 3. *Coregonus macrophthalmus* Nüsslin, 1882, syntypes N = 7. *C. wartmanni* Bloch, 1784, non- type, NMBE-1076206, female.

Morphological characters	* C. gutturosus*			* C. arenicolus*				* C. macrophthalmus*			* C. wartmanni*
	Non-types (N=10)			Holotype	Paratypes (N=3)			Syntypes (N=7)			Non-type
	*N* -total	Mean±StDev	Range		*N* -total	Mean±StDev	Range	*N* -total	Mean±StDev	Range	
**SL (mm)**	10	220.4±36.8	(169–292)	296.0	3	301.3±12.5	(289–314)	7	213.9±12.4	(193–235)	301
**Percentage of standard length**											
**PelvFB**	10	4.1±0.2	(3.7–4.4)	3.9	3	4.4±0.3	(3.9–4.6)	7	3.8±0.3	(3.3–4.2)	3.8
**PelvFS**	10	6.1±0.4	(5.3–6.7)	5.4	3	5.7±0.5	(5.2–6.1)	7	5.7±0.7	(4.8–6.9)	6.5
**PelvF**	10	17.1±1.2	(15.4–19.1)	17.3	3	17.3±0.7	(16.8–18.1)	7	16.5±0.9	(15.2–17.6)	15.4
**PecFB**	10	3.4±0.3	(2.9–3.9)	3.3	3	3.4±0.2	(3.2–3.5)	7	3.2±0.4	(2.8–3.9)	3
**PecF1**	10	16.8±1.1	(14.8–18.9)	14.4	3	16.8±0.5	(14.4–17.2)	7	16.4±1.2	(15.1–18.1)	16
**PecF2**	10	18.2±1.5	(16.8–20.3)	15.7	3	17.4±0.8	(15.7–18)	7	17.1±1	(15.6–18.4)	17
**DFB**	10	11.9±0.7	(10.7–12.8)	12.2	3	12.2±1.1	(11.0–13.1)	7	11.6±0.7	(10.8–12.4)	11.2
**DFAe**	10	19.3±1.3	(17.6–21.6)	18.9	3	19.2±1.2	(18.0–20.3)	7	18.2±1.2	(16.6–19.6)	16.6
**DFAd**	10	20.4±1.1	(19.0–22.2)	20.2	3	20.5±1.3	(19.3–21.9)	7	19.2±1.3	(17.2–20.5)	18.2
**DFPe**	10	5.5±0.7	(4.8–7.0)	5.2	3	5.5±0.2	(5.2–5.7)	7	5.2±0.6	(4.4–5.9)	4.6
**AFB**	10	12.4±0.8	(11.4–13.4)	11.9	3	11.5±1.1	(10.7–12.7)	7	12.3±1.3	(10.6–14.2)	12.5
**AFAe**	10	12.3±1.0	(10.7–13.9)	13.2	3	13.3±0.5	(12.9–13.8)	5	12.1±1.3	(10.8–13.9)	11.1
**AdFB**	10	5.6±0.4	(4.9–6.1)	5.7	3	5.0±1.3	(3.7–6.2)	7	5.3±0.3	(4.9–5.8)	4
**CF**	9	23.2±1.9	(20.8–25.6)	na	2	24±0.1	(24–24.1)	3	22.6±1.2	(21.8–24)	23.8
**CD**	10	7.4±0.4	(6.7–8.2)	7.7	3	8.1±0.1	(7.7–8.2)	7	7.4±0.4	(6.9–8)	7.4
**CL**	10	12.9±0.8	(11.5–13.9)	14.4	3	12.9±0.8	(12.0–14.4)	7	13.8±1.3	(12.4–16.5)	13.2
**PAdC**	10	18.5±0.6	(17.4–19.3)	19.6	3	17.2±2.2	(14.6–19.6)	7	18.9±1.1	(17.6–20.2)	17.8
**DHL**	10	16.8±0.8	(15.4–18.1)	15.1	3	15.1±0.2	(14.8–15.3)	7	15.7±0.8	(14.4–16.5)	14.5
**PreP**	10	52.7±1.4	(50.4–54.1)	49.5	3	50.6±0.5	(49.5–51.0)	7	51.7±1.7	(48.1–53.1)	50.7
**PreA**	10	77.9±1.4	(76.0–80.4)	75.0	3	79.2±0.9	(75.0–80.3)	7	76.8±0.9	(75.7–78.3)	77.4
**PreD**	10	48.4±0.9	(46.8–49.6)	47.9	3	49.2±0.5	(47.9–49.6)	7	47±1	(45.8–48.5)	47.3
**BD**	10	25.9±1.9	(22.9–29.6)	24.4	3	26.2±0.8	(24.4–27.1)	7	23.5±1.9	(21.0–26.9)	23.5
**PostD**	10	43±1.5	(40.6–45.4)	44.8	3	42.2±1.7	(40.4–44.8)	7	43±1.4	(41.6–45.7)	44
**TL**	9	120.2±3	(115.1–124.3)	na	2	121.5±2.9	(119.4–123.5)	3	119.2±0.7	(118.9–120)	120.6
**HL (mm)**	10	50.3±7.1	(41.6–62.4)	61.8	3	63.7±3.9	(59.6–67.2)	7	47.5±3.2	(42.6–51.3)	64.7
**Percentage of head length**											
**SN**	10	22.4±0.7	(21.1–23.1)	23.4	3	23.4±1.6	(21.6–24.6)	7	21.7±2.7	(18–25.6)	24
**ED**	10	21.1±1.4	(19.4–23)	19.6	3	17.7±0.4	(17.3–19.6)	7	24.1±1.7	(21.3–26.1)	18.9
**EC**	10	26.9±1.2	(25.4–29.3)	25.7	3	25±0.8	(24.1–25.7)	7	28.9±2	(25.4–30.8)	23.9
**EH**	10	21.3±0.6	(20.5–22.6)	20.8	3	19.6±0.9	(18.8–20.8)	7	23.2±2.1	(19.5–25.6)	19
**ES**	10	4.8±0.7	(3.5–5.6)	4.9	3	5.2±0.5	(4.6–5.5)	7	3.9±0.8	(2.7–4.6)	5.1
**PostO**	10	52.8±1	(51.5–54.4)	55.7	3	54±1	(53–55.7)	7	50.2±1.9	(48.5–53.2)	53.4
**HD**	10	74.2±3.2	(69.9–80.6)	68.2	3	72.6±3	(68.2–75)	7	68.6±4.8	(61.6–76.3)	67.6
**MW**	10	9.8±0.6	(9–11.2)	10.4	3	10.5±0.5	(10–11)	6	10.1±0.8	(8.7–11.1)	10.6
**UJ**	10	26.8±1.2	(24.6–29)	27.2	3	29.3±1	(27.2–30.1)	7	30.3±2.3	(26.7–33.8)	28.8
**LJ**	10	36.6±1.4	(34.3–39.1)	37.8	3	38.7±0.6	(37.8–39.1)	7	42.2±2	(40–44.4)	43.5
**M**	10	18.9±1.3	(17.3–21.7)	21.1	3	19.7±0.9	(18.6–21.1)	5	23.1±1.9	(20.1–24.7)	22
**SD**	10	10.2±0.8	(9.3–11.9)	9.7	3	10.9±1.3	(9.7–12.3)	7	7.4±1.2	(5.5–9.5)	6.8
**SW**	10	15.1±1.6	(12.3–17.6)	14.9	3	17.8±0.7	(14.9–18.5)	7	15.6±1.2	(14.1–17.4)	15
**HW**	10	56.1±4.3	(46.7–62.3)	51.8	3	50.8±0.5	(50.5–51.8)	7	41.6±1.5	(39.3–43.3)	45.5
**IOW**	10	28.4±1.7	(26.2–31.6)	29.6	3	29.7±1	(28.8–30.8)	7	26.1±1.6	(23.8–28.9)	24.2
**INW**	10	11.9±0.7	(10.7–12.7)	12.0	3	13.7±0.1	(12–13.8)	5	11.9±1.3	(10.7–14.1)	12.7
**LJW**	10	7.7±1	(6.8–9.9)	7.8	3	8.1±0.3	(7.8–8.5)	5	7.8±1	(6.4–8.8)	8.1
**UJW**	10	25.2±1.2	(23.1–26.8)	24.9	3	26.4±0.8	(24.9–27.2)	7	21.6±1.9	(18.6–24.6)	22.7
**MGR**	9	6.9±1.3	(4.1–8.7)	9.9	2	10.2±0.6	(9.8–10.6)	4	12.5±1.4	(11.6–14.7)	10.8
**LGR**	9	8.2±1.4	(6.7–10.6)	10.9	2	11.5±0.6	(10.9–12)	4	14.6±1.2	(13.3–16.1)	11.3
**UA**	na	na	na	na	na	na	na	na	na	na	na
**LA**	na	na	na	na	na	na	na	na	na	na	na
**Meristic character**	***N*-total**	**Mode**	**Range**		***N*-total**	**Mode**	**Range**	***N*-total**	**Mode**	**Range**	
**PelvF nbranched**	10	1	(1–1)	1	3	1	(1–1)	7	1	(1–1)	1
**PelvF branched**	10	11	(9–11)	11	3	11	(11–11)	7	10	(10–11)	12
**PecF nbranched**	10	1	(1–1)	1	3	1	(1–1)	7	1	(1–1)	1
**PecF branched**	10	13	(12–14)	12	3	12	(12–14)	7	15	(14–15)	16
**DF unbranched**	10	4	(3–4)	4	3	4	(4–4)	7	4	(4–4)	4
**DF branched**	10	10	(9–10)	9	3	10	(9–10)	7	9	(9–10)	10
**AF unbranched**	10	3	(3–4)	3	3	3	(3–3)	7	3	(3–4)	4
**AF branched**	10	11	(10–12)	10	3	10	(10–11)	7	11	(10–13)	13
**LS**	10	78	(76–82)	82	3	na	(82–90)	7	80	(73–80)	84
**PDS**	10	33	(31–35)	36	3	na	(36–44)	7	32	(32–36)	34
**TDS**	10	10	(9–10)	10	3	10	(10–11)	7	9	(9–10)	10
**TAS**	10	8	(7–9)	8	3	9	(8–9)	7	7	(7–9)	8
**TPS**	10	8	(8–9)	8	3	9	(8–9)	7	7	(7–9)	8
**UGR**	9	7	(7–9)	9	2	na	(9–12)	4	14	(12–14)	11
**LGR**	9	10	(9–12)	13	2	na	(13–19)	4	24	(22–24)	23
**total GR**	9	19	(16–21)	22	2	na	(22–31)	4	36	(36–38)	34

**Figure 12.**
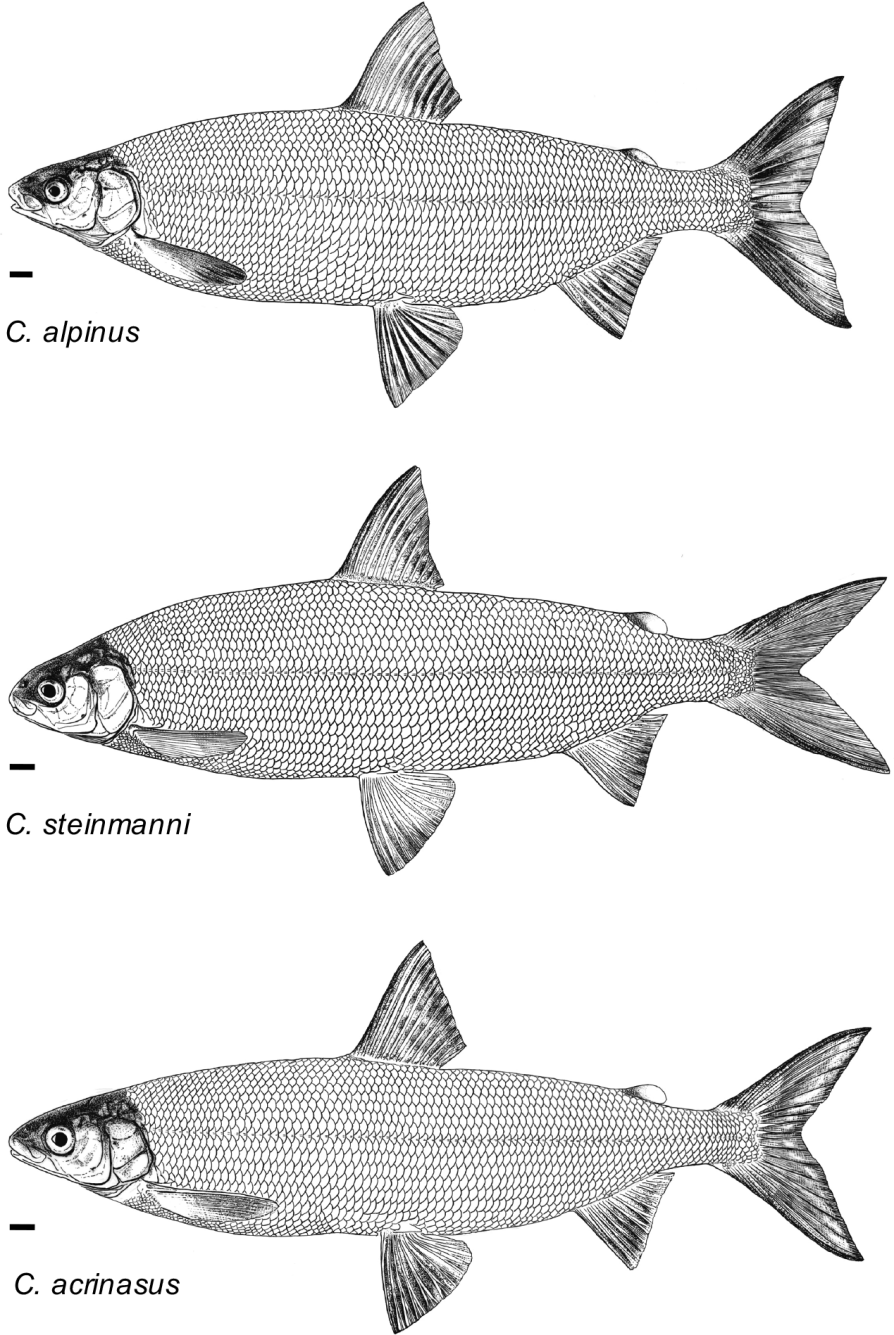
Illustrations of specimens of each species from Lake Thun. From top to bottom: *Coregonus alpinus*: non-type, NMBE-1077244, 343 mm, male; *Coregonus steinmanni*: paratype, NMBE-1077218, 289.5 mm, male; *Coregonus acrinasus*: paratype, NMBE-1077270, 270 mm, male; *Coregonus fatioi*: nontype, NMBE-1077138, 267 mm, male; *Coregonus albellus*: non-type, NMBE-1077188, 215 mm, male; *Coregonus profundus*: non-type, Eawag-123850, 195 mm, male. The black scale (1cm) below each fish acts as a reference for the actual size of the specimen.

**Figure 12.**
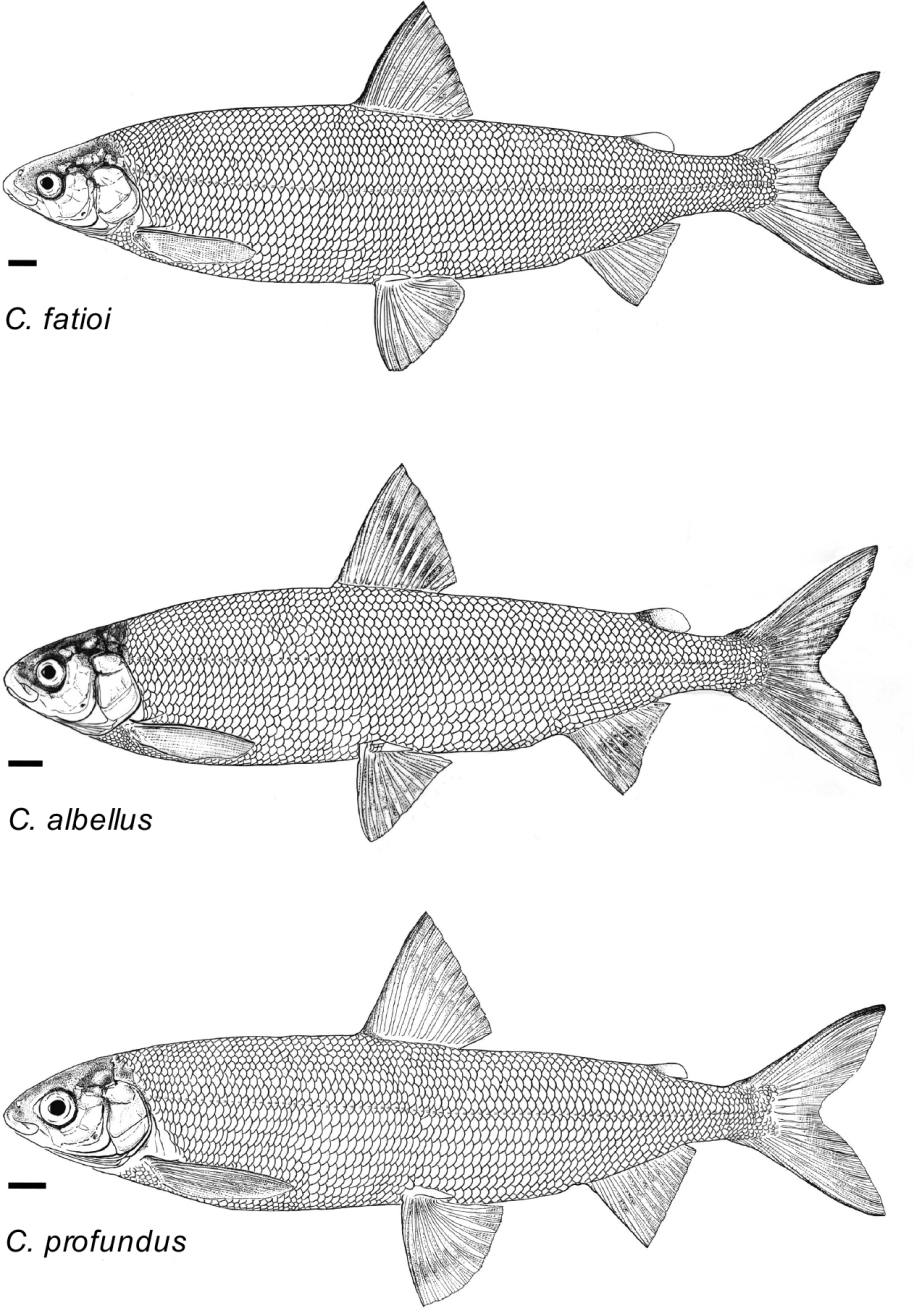
Continued.

## Discussion

Phylogeographic studies have shown that the pre-alpine whitefish are a monophyletic clade, most closely related to whitefish from northern Europe. The clade is of hybrid origin involving two glacial lineages that must have come into secondary contact several hundred thousand years after their separation. Independent events of intra-lacustrine speciation led to a series of adaptive radiations in each major lake system of the northern pre-Alps (Hudson et al. 2011). Up to six endemic species can be found in the most diverse of these adaptive radiations (Vonlanthen et al. 2012; Hudson et al. 2016; Dönz et al. 2018). Here we take an integrative taxonomic approach, combining genetic assignments (Dönz et al. 2018) with morphological and meristic traits and multivariate statistical methods to delineate species and revise the taxonomy of the whitefish radiation of lakes Thun and Brienz in the western Aare catchment of Switzerland. We distinguish and characterize seven whitefish species from these lakes. Three of them occur in both lakes and three may be unique to Lake Thun and one may be unique to Lake Brienz. The three species that occur in both lakes have been described more than 130 years ago. Two of these species, *C. alpinus* and *C. albellus*, were described by Fatio (1885 and 1890 respectively). The third species, *C. fatioi*, was renamed by Kottelat (1997), but first described by Fatio (1890). Kottelat (1997) proposed *C. fatioi* as a replacement name, since the name given by Fatio (1890) was preoccupied by another species described by Fatio (1885). Our own earlier research combining genetics and ecology had shown that a fourth species is present in both lakes (Dönz et al. 2018). However, recent whole-genome data (De-Kayne et al. unpublished) csuggest that this newly discovered species is genetically different between lakes. We thus describe this species here as *Coregonus steinmanni* sp. nov. for the specimens from Lake Thun and those from Lake Brienz we designate as *C. brienzii*. Our earlier research (Hudson et al. 2011; Vonlanthen et al. 2012; Dönz et al. 2018) had further revealed that Lake Thun harbours two additional undescribed species and we describe these here as *Coregonus profundus* sp. nov. and *Coregonus acrinasus* sp. nov. Consistent with previous work (Dönz et al. 2018), recent genomic analyses (whole-genome data: De-Kayne et al. unpublished) find that the three species, *C. alpinus*, *C. fatioi* and *C. albellus*, occurring in both lakes cluster by species, whereas *C. steinmanni* from Lake Thun and *C. brienzii* from Lake Brienz (formerly *C. steinmanni* from Lake Brienz; Dönz et al. 2018) are not each others closest relatives. Interestingly, we also find morphological relationships to differ between the lakes; in Lake Thun *C. steinmanni* groups in morphospace with *C. alpinus*, whereas in Lake Brienz *C. brienzii* groups in morphospace with *C. fatioi*.

Based on genetic, morphological and ecological data at least two species from the Lake Thun-Brienz radiation, namely *C. albellus* (since at least 2004: Bittner 2009; Vonlanthen and Périat 2018; this study) and *C. profundus* (since at least 2016: this study) have colonized Lake Biel. There are no indications and no historical records that the Bernese cantonal officials have translocated any whitefish from other lakes into Lake Biel. Importantly, Steinmann (1950) already mentions that fishermen reported that suddenly after the Jura water correction, whitefish that resembled *C. albellus* (common name Brienzlig), appeared in Lake Biel. It is hence possible, that colonization of Lake Biel happened in recent times through the river Aare, which became connected with Lake Biel after the Jura water correction of 1868–1878. At least one of the species, *C. albellus*, has likely established a self-sustaining population in Lake Biel, since a reasonable number of ripe specimens of this species have been caught repeatedly over several years during the typical spawning period of this species (late summer: September-October; Bittner 2009; Vonlanthen and Périat 2018; Suppl. material 1: Figure S9). Today, Lake Biel harbours two native whitefish species, *C. confusus*, Fatio 1885 and *C. palaea*, Cuvier 1829 (Kottelat and Freyhof 2007) but it used to harbour a third species known by its local name as "Balch-Pfärrit" (Fatio 1885), which is extinct today (Vonlanthen et al. 2012). Fatio (1890: Page 192) mentions that the "Balch-Pfärrit" was intermediate in phenotype between *C. confusus* and *C. palaea* of Lake Biel and has been considered by the local fishermen as a natural hybrid between the latter two species. Yet, based on the overall phenotype and ecological characters (spawning season and depth) Fatio (1885, 1890) considered the "Balch-Pfärrit" as an independent albeit variable species. This species increased in abundance during the study period of Fatio, which coincided with the completion of the Jura water correction from 1868–1878 that by passed the river Aare from Lake Thun to Lake Biel. This led some fishermen to suggest, that the "Balch-Pfärrit" might have come from Lake Thun. Fatio dismissed this because these fish did not resemble the species known by then from Lake Thun, this being *C. albellus*, *C. alpinus* and C.*fatioi*. He rather suggested the rise in abundance of the "Balch-Pfärrit" may have been caused by the lake level reduction of Lake Biel following the Jura water correction. The Lake Biel and Lake Neuchatel species’ *C. confusus*, *C. palaea*, and *C. candidus* form distinct monophyletic clades in population neighbour-joining trees and one genetic cluster in a structure analysis, based on microsatellite and genomic AFLP loci (Hudson et al. 2011, 2016). Based on this and on the fact that the historically reported three whitefish species of Lake Biel (*C. confusus*, *C. palaea* and the "Balch-Pfärrit") were all winter spawners (Fatio 1885, 1890; Steinmann 1950) and that the ripe whitefish, that were caught in recent years in late summer in Lake Biel, were assigned with high probability to whitefish species from Lake Thun (Bittner 2009; Suppl. material 1: Figure S9), suggests that the ripe specimens caught in late summer in Lake Biel are unlikely to be the extinct "Balch-Pfärrit". Instead, we suggest that two Lake Thun whitefish species, *C. profundus* and *C. albellus*, have colonized Lake Biel. Interestingly, Steinmann (1950) reports that he was able to examine two ripe whitefish in September 1944 from Lake Biel that he thought resembled, based on their morphology (e.g., gill raker number, eye size), very much *C. albellus* from lakes Thun and Brienz.

Lakes Thun and Brienz in the Bernes Highlands today harbour the most speciose pre-alpine whitefish radiation. These lakes have also suffered the least anthropogenic pressures of all the large pre-alpine lakes in Switzerland. Species delineation and description in such rich radiations require an integrative approach to taxonomy, combining morphology with population genetics and ecology and extensive contemporary and historical specimen collections. Such work is also much needed for conservation-minded fisheries management because, as we have shown here and others before us (Douglas and Brunner 2002; Bittner 2009; Dönz et al. 2018), human-made changes to the connectivity of water bodies as well as deliberate introductions, are increasing the distribution ranges of species and cause previously isolated biota to mix.

## Supplementary Material

XML Treatment for Coregonus albellus

XML Treatment for Coregonus alpinus

XML Treatment for Coregonus fatioi

XML Treatment for Coregonus steinmanni

XML Treatment for C. brienzii

XML Treatment for Coregonus profundus

XML Treatment for Coregonus acrinasus

XML Treatment for Coregonus gutturosus

XML Treatment for Coregonus arenicolus

XML Treatment for Coregonus macrophthalmus

XML Treatment for Coregonus wartmanni

## Supplementary materials

Supplementary material 1 Tables S1-S8, Figures S1-S11 Data type measurements https://binary.pensoft.net/file/473479Oliver M. Selz, Carmela J. Dönz, Pascal Vonlanthen, Ole Seehausen
