# Supplementary material for: A taxonomic revision of the whitefish of lakes Brienz and Thun, Switzerland, with descriptions of four new species (Teleostei, Coregonidae)
Source: Zookeys. 2020 Nov 9;989:79–162. doi: 10.3897/zookeys.989.32822 (PMC7669820; doi:10.3897/zookeys.989.32822)

Table S1. PC-loadings of all characters (values > 0.1 or < -0.1 are highlighted in bold), eigenvalues, proportion of explained variance, and R-squared from a linear regression of shape vs. isosize of the retained first three PC-axes from a PCA with all contemporary specimens of the formerly described species *C. alpinus*, *C. albellus,* and *C. fatioi* and their types and the newly described species *C. steinmanni* and *C. profundus* from Lake Thun. See Table 1 and Figure S1 and S2 for descriptions and illustrations of each morphological character.

	**shape PC1**	**shape PC2**	**shape PC3**
**Eigenvalue**	0.121	0.033	0.013
**Variance**	45.63%	12.38%	12.04%
**R-squared**	<0.1	0.29	0.05
		**PCA-loadings**	
**Character**			
**PELVFB**	**-0.14**	**-0.20**	**0.15**
**PELVF**	-0.09	0.08	0.01
**PECFB**	**-0.14**	0.03	-0.02
**DFB**	**-0.13**	**-0.10**	**0.19**
**DFPe**	0.02	**0.24**	**0.14**
**AFB**	-0.07	0.02	0.08
**AdFB**	**0.21**	**0.47**	**-0.25**
**CD**	**-0.14**	**-0.20**	**0.25**
**CL**	0.07	**-0.18**	**0.15**
**PAdC**	0.02	0.02	0.02
**DHL**	-0.09	**0.17**	-0.07
**PreP**	-0.08	-0.05	0.02
**PreA**	-0.07	-0.10	0.06
**SL**	-0.05	-0.09	0.07
**PreD**	-0.09	-0.09	0.05
**BD**	-0.06	**-0.28**	**0.22**
**PostD**	0.02	-0.07	0.07
**SN**	-0.01	0.08	-0.03
**ED**	-0.04	**0.25**	**-0.15**
**EC**	**-0.12**	**0.27**	-0.09
**PostO**	-0.05	-0.02	-0.01
**HL**	-0.06	0.06	-0.03
**HD**	-0.06	0.05	-0.05
**LJ**	0.00	**0.15**	-0.07
**HW**	-0.09	0.04	-0.03
**IOW**	-0.06	0.01	-0.09
**LJW**	0.01	**-0.50**	**-0.79**
**MGR**	**0.63**	**-0.11**	**0.11**
**LGR**	**0.63**	-0.08	0.07
**LA**	0.01	**0.13**	-0.01

Table S2. PC-loadings of all characters (values > 0.1 or < -0.1 are highlighted in bold), eigenvalues, proportion of explained variance, and R-squared from a linear regression of shape vs. isosize of the retained first three PC-axes from a PCA with all contemporary specimens of the six species *C. alpinus*, *C. steinmanni*, *C. fatioi*, *C. albellus*, *C. acrinasus* and *C. profundus* from Lake Thun. See Table 1 and Figure S1 and S2 for descriptions and illustrations of each morphological character.

	shape PC1	shape PC2	shape PC3
**Eigenvalue**	0.156	0.054	0.040
**Variance**	35.42%	12.21%	8.94%
**R-squared**	<0.1	<0.01	0.21
		**PCA-loadings**	
**Character**			
**PELVFB**	0.08	0.01	**-0.25**
**PELVFS**	-0.06	-0.10	**-0.12**
**PELVF**	0.04	0.08	0.01
**PECFB**	0.09	0.07	-0.01
**PECF1**	0.04	**0.16**	0.07
**PECF2**	0.04	**0.19**	**0.11**
**DFB**	0.09	-0.07	**-0.17**
**DFAe**	0.07	0.05	-0.09
**DFAd**	0.06	0.03	-0.08
**DFPe**	-0.01	-0.01	0.08
**AFB**	0.03	0.01	-0.08
**AFAe**	0.06	**0.10**	-0.01
**AdFB**	**-0.18**	0.10	**0.39**
**CF**	0.02	0.04	-0.07
**CD**	0.09	**-0.11**	**-0.25**
**CL**	-0.06	**-0.13**	**-0.19**
**PAdC**	-0.04	-0.03	-0.02
**DHL**	0.04	0.06	**0.11**
**PreP**	0.04	0.00	-0.07
**PreA**	0.03	-0.03	**-0.12**
**SL**	0.01	-0.04	**-0.12**
**TL**	0.01	-0.03	**-0.10**
**PreD**	0.05	-0.03	**-0.12**
**BD**	0.03	-0.08	**-0.30**
**PostD**	-0.04	-0.06	**-0.10**
**SN**	-0.02	0.03	0.04
**ED**	0.00	0.05	**0.19**
**EC**	0.08	0.04	**0.19**
**EH**	-0.01	0.07	**0.21**
**PostO**	0.01	0.01	-0.03
**HL**	0.02	0.02	0.02
**HD**	0.03	0.06	0.02
**MW**	-0.03	0.03	**0.24**
**UJ**	-0.07	0.03	**0.15**
**LJ**	-0.03	0.01	**0.11**
**M**	-0.07	-0.01	**0.16**
**SD**	**0.11**	**0.23**	-0.08
**SW**	-0.08	0.04	0.06
**HW**	0.04	0.08	-0.01
**IOW**	0.03	0.07	0.01
**INW**	-0.02	-0.04	**-0.10**
**LJW**	-0.06	**0.29**	**-0.23**
**UJW**	0.00	**0.20**	**0.16**
**ES**	**0.61**	**-0.62**	**0.28**
**MGR**	**-0.49**	**-0.37**	0.01
**LGR**	**-0.49**	**-0.33**	0.02
**UA**	-0.05	-0.05	-0.01
**LA**	-0.03	-0.02	0.07

Table S3. PC-loadings of all characters (values > 0.1 or < -0.1 are highlighted in bold), eigenvalues, proportion of explained variance, and R-squared from a linear regression of shape vs. isosize of the retained first three PC-axes from a PCA with all contemporary specimens of the four species *C. alpinus*, *C. fatioi,* and *C. albellus* and the newly described species *C. brienzii* from Lake Brienz. Only specimens smaller than 163.5 mm were used for the analysis to overcome allometry issues. See Table 1 and Figure S1 and S2 for descriptions and illustrations of each morphological character.

	**shape PC1**	**shape PC2**	**shape PC3**
**Eigenvalue**	0.160	0.041	0.021
**Variance**	42.58%	10.85%	7.25%
**R-squared**	0.1	<0.1	<0.01
		**PCA-loadings**	
**Character**			
**PELVFB**	-0.02	0.10	0.04
**PELVFS**	0.02	0.06	**-0.24**
**PELVF**	-0.04	0.09	**-0.14**
**PECFB**	0.01	0.07	0.01
**PECF1**	-0.07	**0.13**	-0.03
**PECF2**	**-0.11**	0.08	-0.06
**DFB**	0.04	**0.11**	**-0.14**
**DFAe**	-0.04	**0.17**	**-0.15**
**DFAd**	-0.05	**0.17**	**-0.16**
**DFPe**	-0.07	**0.24**	**-0.11**
**AFB**	0.02	**0.14**	0.04
**AFAe**	0.01	**0.19**	-0.10
**AdFB**	-0.10	**-0.59**	**-0.40**
**CF**	0.01	0.10	-0.01
**CD**	**0.15**	**0.15**	0.01
**CL**	**0.17**	**-0.15**	**-0.12**
**PAdC**	0.05	**-0.14**	-0.09
**DHL**	-0.01	0.03	0.05
**PreP**	**0.11**	0.05	-0.08
**PreA**	**0.12**	0.04	-0.09
**SL**	**0.12**	-0.02	**-0.14**
**TL**	**0.11**	0.01	**-0.11**
**PreD**	**0.12**	0.05	-0.04
**BD**	**0.19**	**0.11**	-0.02
**PostD**	**0.13**	-0.05	-0.09
**SN**	0.01	0.02	0.07
**ED**	**-0.14**	-0.03	0.03
**EC**	**-0.12**	-0.02	0.04
**EH**	**-0.17**	-0.02	0.05
**PostO**	0.02	**0.10**	-0.04
**HL**	-0.02	0.06	0.00
**HD**	0.04	0.03	0.02
**MW**	-0.03	-0.05	0.03
**UJ**	**-0.13**	0.03	**0.12**
**LJ**	**-0.10**	-0.03	**0.14**
**M**	**-0.16**	0.01	0.08
**SD**	**0.11**	**-0.17**	0.05
**SW**	-0.04	-0.07	**0.17**
**HW**	0.04	-0.07	0.09
**IOW**	0.03	-0.01	-0.02
**INW**	0.04	-0.09	0.05
**LJW**	**0.14**	**-0.42**	0.03
**UJW**	0.01	**-0.12**	0.08
**ES**	**0.55**	-0.07	**0.55**
**MGR**	**-0.38**	-0.09	**0.30**
**LGR**	**-0.40**	-0.09	**0.26**
**UA**	-0.09	**0.10**	**0.12**
**LA**	-0.07	**-0.15**	-0.05

Table S4. PC-loadings of all characters (values > 0.1 or < -0.1 are highlighted in bold), eigenvalues, proportion of explained variance, and R-squared from a linear regression of shape vs. isosize of the retained first three PC-axes from a PCA with all specimens of the three species *C. alpinus*, *C. fatioi,* and *C. albellus* and the newly described species *C. brienzii* from Lake Brienz. Only specimens larger than 163.5 mm were used for the analysis to overcome allometry issues. See Table 1 and Figure S1 and S2 for descriptions and illustrations of each morphological character.

	**shape PC1**	**shape PC2**	**shape PC3**
**Eigenvalue**	0.082	0.041	0.025
**Variance**	27.27%	13.58%	12.18%
**R-squared**	0.14	0.18	-0.02
		**PCA-loadings**	
**Character**			
**PELVFB**	0.04	**-0.12**	-0.08
**PELVFS**	**0.21**	**0.20**	0.02
**PELVF**	**0.13**	-0.04	-0.09
**PECFB**	0.02	-0.07	-0.01
**PECF1**	0.04	0.10	**-0.16**
**PECF2**	0.02	0.06	**-0.15**
**DFB**	**0.15**	-0.07	0.02
**DFAe**	**0.20**	-0.02	**-0.12**
**DFAd**	**0.20**	-0.03	-0.10
**DFPe**	0.01	0.06	**-0.19**
**AFB**	0.01	-0.09	**-0.11**
**AFAe**	**0.17**	-0.03	**-0.15**
**AdFB**	-0.04	-0.08	**0.32**
**CF**	0.01	-0.01	-0.08
**CD**	**0.14**	-0.05	-0.03
**CL**	-0.10	-0.08	0.07
**PAdC**	-0.03	**-0.15**	0.08
**DHL**	0.01	-0.02	-0.02
**PreP**	0.02	-0.06	0.10
**PreA**	0.06	-0.07	0.02
**SL**	0.04	-0.08	0.03
**TL**	0.01	-0.09	0.02
**PreD**	0.05	-0.05	0.02
**BD**	**0.11**	**-0.11**	**0.15**
**PostD**	0.00	**-0.14**	0.08
**SN**	-0.04	**-0.16**	0.04
**ED**	-0.03	**0.15**	**-0.12**
**EC**	0.03	**0.17**	-0.09
**EH**	-0.02	**0.16**	**-0.13**
**PostO**	0.05	0.06	-0.07
**HL**	0.01	0.02	-0.05
**HD**	0.01	-0.03	0.01
**MW**	-0.03	0.04	0.00
**UJ**	**-0.16**	-0.06	-0.09
**LJ**	**-0.14**	0.10	0.00
**M**	-0.10	**0.13**	**-0.14**
**SD**	**0.17**	**0.22**	-0.01
**SW**	**-0.13**	-0.10	0.03
**HW**	-0.03	**-0.15**	0.07
**IOW**	0.02	**-0.13**	-0.01
**INW**	0.03	-0.01	0.10
**LJW**	**-0.33**	**-0.30**	**0.41**
**UJW**	-0.04	**-0.16**	0.03
**ES**	**0.23**	**0.55**	**0.63**
**MGR**	**-0.49**	**0.23**	-0.07
**LGR**	**-0.49**	**0.31**	-0.08
**UA**	0.02	0.06	-0.09
**LA**	-0.01	-0.04	-0.02

Table S5. PC-loadings of all characters (values > 0.1 or < -0.1 are highlighted in bold), eigenvalues, proportion of explained variance, and R-squared from a linear regression of shape vs. isosize of the retained first three PC-axes from a PCA with all whitefish species from Lake Thun and the three specimens of whitefish from Lake Biel that were assigned by genotype to *C. albellus* or *C. profundus*. See Table 1 and Figure S1 and S2 for descriptions and illustrations of each morphological character.

	**shape PC1**	**shape PC2**	**shape PC3**
**Eigenvalue**	0.155	0.054	0.040
**Variance**	34.94%	12.09%	9.12%
**R-squared**	<0.1	<0.01	0.3
		**PCA-loadings**	
**Character**			
**PELVFB**	0.08	0.01	**0.27**
**PELVFS**	-0.06	**-0.10**	0.09
**PELVF**	0.04	0.08	-0.02
**PECFB**	0.09	0.07	0.02
**PECF1**	0.03	**0.16**	-0.07
**PECF2**	0.04	**0.19**	**-0.11**
**DFB**	0.09	-0.07	**0.16**
**DFAe**	0.07	0.05	0.07
**DFAd**	0.06	0.03	0.05
**DFPe**	-0.01	0.00	**-0.10**
**AFB**	0.03	0.01	0.06
**AFAe**	0.06	0.10	-0.01
**AdFB**	**-0.18**	0.09	**-0.37**
**CF**	0.02	0.04	0.06
**CD**	0.09	**-0.11**	**0.23**
**CL**	-0.06	**-0.13**	**0.20**
**PAdC**	-0.04	-0.03	0.02
**DHL**	0.04	0.06	**-0.12**
**PreP**	0.04	0.00	0.07
**PreA**	0.03	-0.03	**0.13**
**SL**	0.01	-0.04	**0.12**
**TL**	0.01	-0.03	**0.10**
**PreD**	0.05	-0.02	**0.12**
**BD**	0.03	-0.08	**0.29**
**PostD**	-0.04	-0.06	**0.11**
**SN**	-0.02	0.03	-0.03
**ED**	0.00	0.05	**-0.21**
**EC**	0.07	0.04	**-0.21**
**EH**	-0.01	0.07	**-0.22**
**PostO**	0.01	0.01	0.03
**HL**	0.02	0.02	-0.03
**HD**	0.03	0.06	-0.02
**MW**	-0.04	0.04	**-0.24**
**UJ**	-0.07	0.03	**-0.14**
**LJ**	-0.03	0.01	**-0.12**
**M**	-0.07	-0.01	**-0.16**
**SD**	**0.11**	**0.23**	0.09
**SW**	-0.08	0.05	-0.07
**HW**	0.04	0.08	0.02
**IOW**	0.03	0.07	0.01
**INW**	-0.02	-0.04	0.09
**LJW**	-0.06	**0.29**	**0.30**
**UJW**	0.00	**0.20**	**-0.13**
**ES**	**0.61**	**-0.62**	**-0.24**
**MGR**	**-0.49**	**-0.37**	0.01
**LGR**	**-0.49**	**-0.33**	0.00
**UA**	-0.05	-0.04	0.00
**LA**	-0.03	-0.02	-0.09

Table S6. Frequency of occurrence of meristic values in the six whitefish species from Lake Thun and the four whitefish species from Lake Brienz. The highest frequency for each species is highlighted in bold.

Species	Lake	Ntotal	number of lateral line scales																							
			70	71	72	73	74	75	76	77	78	79	80	81	82	83	84	85	86	87	88	89	90	91	92	93
C. albellus	Thun	34				1			2	6	3	**12**		3	1	2	1	2	1							
	Brienz	32	2			1	3	2	**7**	6	4	2	3	1			1									
	Thun+Brienz	66	2			2	3	2	9	12	7	**14**	3	4	1	2	2	2	1							
C. fatioi	Thun	30									4	3	4	1	2	1	4	4	**6**							1
	Brienz	30										1	1	3	1	3	2	4	**7**	3	2		1	1	1	
	Thun+Brienz	60									4	4	5	4	3	4	6	8	**13**	3	2		1	1	1	1
C. alpinus	Thun	21								1	3	1	3	**4**	**4**		3	1								1
	Brienz	9											1	1	1		**2**		**2**	1	1					
	Thun+Brienz	30								1	3	1	4	**5**	**5**		**5**	1	2	1	1					1
C. brienzii	Brienz	13											2	1		1			**4**	1	1	2		1		
C. steinmanni	Thun	12									**3**		2		2		2	2		1						
C. profundus	Thun	28							1	2	1	2	4	3	2	4	**5**		2			1	1			
C. acrinasus	Thun	26										2	6	3			**8**	4	1	1	1					

Species	Lake	Ntotal	PDS																		
			**26**	**27**	**28**	**29**	**30**	**31**	**32**	**33**	**34**	**35**	**36**	**37**	**38**	**39**	**40**	**41**	**42**	**43**	**44**
C. albellus	Thun	34				1	1	3	4	5	**9**	2	7	2							
	Brienz	32	1	1	1		5	**10**	8	3	1	2									
	Thun+Brienz	66	1	1	1	1	6	**13**	12	8	10	4	7	2							
C. fatioi	Thun	30					1	2	**7**	3		2	6	3	4		1				1
	Brienz	30					1		1	2	**9**	5	5	3	3		1				
	Thun+Brienz	59					2	2	8	5	9	7	**11**	6	7		2				1
C. alpinus	Thun	21							1	3	2	**4**	**4**	2	3	1			1		
	Brienz	9							1	**2**	1	1	1	1				1	1		
	Thun+Brienz	30							2	**5**	3	**5**	**5**	3	3	1		1	2		
C. brienzii	Brienz	13							2	1	**3**	**3**	2	1			1				
C. steinmanni	Thun	12							1	1	2	**3**	3				2				
C. profundus	Thun	28							5	1	**10**	4	3	2	3						
C. acrinasus	Thun	26								2	**6**	5	2	3	5	1		1	1		

Species	Lake	Ntotal	TDS					Ntotal	TAS				Ntotal	TPS			
			**7**	**8**	**9**	**10**	**11**		**6**	**7**	**8**	**9**		**7**	**8**	**9**	**10**
C. albellus	Thun	34		3	**24**	7		34		2	**23**	9	66		5	**29**	
	Brienz	32	4	**22**	6			30	2	**20**	10		30	13	**18**	1	
	Thun+Brienz	66	4	25	**30**	7		64	2	22	**33**	9	30	13	23	**30**	
C. fatioi	Thun	30		3	**18**	8	1	30		5	**18**	7	60	2	8	**20**	
	Brienz	30			13	**17**		30		1	**22**	7	21		13	**17**	
	Thun+Brienz	60		3	**31**	25	1	60		6	**40**	14	9	2	21	**37**	
C. alpinus	Thun	21			5	**14**	2	21			**16**	5	30		7	**14**	
	Brienz	9		1	**4**	**4**		9		3	**6**		0	1	**8**		
	Thun+Brienz	30		1	9	**18**	2	30		3	**22**	5	0	1	**15**	14	
C. brienzii	Brienz	13	1	1	**7**	4		13		4	**9**		26		**12**	1	
C. steinmanni	Thun	12		1	3	**8**		12			**8**	4	0		5	**7**	
C. profundus	Thun	28		7	**17**	4		27	1	9	**18**		0	3	**20**	5	
C. acrinasus	Thun	26			10	**16**		26			**25**	1	0		**17**	9	

Species	Lake	Ntotal	total number of gill rakers																													
			15	16	17	18	19	20	21	22	23	24	25	26	27	28	29	30	31	32	33	34	35	36	37	38	39	40	41	42	43	44
C. albellus	Thun	34																		2	2	1	1	4	6	**7**	6	2	1	1		1
	Brienz	32																					1		5	**7**	4	9	3	3		
	Thun+Brienz	66																		2	2	1	2	4	11	**14**	10	11	4	4		1
C. fatioi	Thun	30																		1		2	2	5	2	**7**	4	3	2	1	1	
	Brienz	30																		3	2	4	**6**	3	3	4	4	1				
	Thun+Brienz	60																		4	2	6	8	8	5	**11**	8	4	2	1	1	
C. alpinus	Thun	21											1	1	3	3	5	**6**		1		1										
	Brienz	9												1	1	**4**	2	1														
	Thun+Brienz	30											1	2	4	**7**	**7**	**7**		1		1										
C. brienzii	Brienz	13																		2		2	1	1	**4**	1	2					
C. steinmanni	Thun	12																2	**4**	1	2	2	1									
C. profundus	Thun	28	1			2		1	**8**	3	6	3	1	2	1																	
C. acrinasus	Thun	26																1		2	4	4	4	**5**	1	2	2	1				

Species	Lake	Ntotal	PelvF # branched				Ntotal	PecF # branched					Ntotal	DF # branched					Ntotal	AF # branched				
			**9**	**10**	**11**	**12**		**13**	**14**	**15**	**16**	**17**		**9**	**10**	**11**	**12**	**13**		**10**	**11**	**12**	**13**	**14**
C. albellus	Thun	34	3	**24**	7		34		1	6	**23**	4	34	2	**17**	13	2		34	1	5	**21**	7	
	Brienz	32	**20**	11	1		32		5	**20**	7		32	3	**25**	3	1		32	1	8	**21**	2	
	Thun+Brienz	66	23	**35**	8		66		6	26	**30**	4	66	5	**42**	16	3		66	2	13	**42**	9	
C.fatioi	Thun	30	3	**20**	7		30		2	6	**17**	5	30		**18**	12			30	1	3	**17**	7	2
	Brienz	30	1	**19**	10		30		2	7	**14**	7	30		10	**16**	3	1	30		3	**22**	5	
	Thun+Brienz	60	4	**39**	17		60		4	13	**31**	12	60		**28**	**28**	3	1	60	1	6	**39**	12	2
C. alpinus	Thun	21		8	**13**		21		5	**9**	6	1	21		7	**10**	3	1	21	2	3	**13**	2	1
	Brienz	9		**5**	4		9			**6**	2	1	9		1	**8**			9		**4**	3	2	
	Thun+Brienz	30		13	**17**		30		5	**15**	8	2	30		8	18	3	1	30	2	7	**16**	4	1
C. brienzii	Brienz	13	1	**9**	3		13			**7**	4	2	13		**5**	**5**	2	1	13		1	**10**	2	
C. steinmanni	Thun	12		**6**	4	2	12		1	**8**	3		12		**8**	3	1		12		4	**7**	1	
C. profundus	Thun	28	2	**20**	6		28	1		4	**20**	3	28	1	**18**	8	1		28		8	**16**	3	1
C. acrinasus	Thun	26	1	**12**	**12**	1	26	1	5	**11**	9		26	1	**15**	8	2		26		**12**	11	3	

Table S7. Frequency of occurrence of the meristic values in the four whitefish species from Lake Constance, Switzerland, *C. gutturosus* Gmelin, 1818, *C. arenicolus* Kottelat, 1997, *C. macrophthalmus* Nüsslin, 1882, *C. wartmanni* Bloch, 1784, and the partially allochthonous whitefish species *C. acrinasus* from Lake Thun, Switzerland, that has ancestry contributions from Lake Constance whitefish. The highest frequency for each species is highlighted in bold.

**Species**	**Lake**	**Ntotal**	**number of lateral line scales**																							
			70	71	72	73	74	75	76	77	78	79	80	81	82	83	84	85	86	87	88	89	90	91	92	93
C.acrinaus	Thun	26									2	6	3			**8**	4	1	1	1						
C.gutturosus	Constance	10							1	2	**3**	1	2		1											
C.arenicolus	Constance	4													**1**				**1**			**1**	**1**			
C.macrophthalmus	Constance	7				1			1		1	1	**3**													
C.wartmanni	Constance	1															**1**									

**Species**	**Lake**	**Ntotal**	**PDS**													
			**31**	**32**	**33**	**34**	**35**	**36**	**37**	**38**	**39**	**40**	**41**	**42**	**43**	**44**
C.acrinaus	Thun	26			2	**6**	5	2	3	5	1		1	1		
C.gutturosus	Constance	9	1	3	**4**		1									
C.arenicolus	Constance	4						**1**	**1**			**1**				**1**
C.macrophthalmus	Constance	7		**3**	2	1		1								
C.wartmanni	Constance	1				**1**										

**Species**	**Lake**	**Ntotal**	**TDS**			**Ntotal**	**TAS**			**Ntotal**	**TPS**		
			**9**	**10**	**11**		**7**	**8**	**9**		**7**	**8**	**9**
C.acrinaus	Thun	26	10	**16**		26		**25**	1	26		**17**	9
C.gutturosus	Constance	9	4	**5**		9	**4**	3	2	9		**8**	1
C.arenicolus	Constance	4		**3**	1	4		1	**3**	4		1	**3**
C.macrophthalmus	Constance	7	**6**	1		7	**4**	2	1	7	**4**	2	1
C.wartmanni	Constance	1		**1**		1		**1**		1		**1**	

**Species**	**Lake**	**Ntotal**	**number of total gill rakers**																													
			15	16	17	18	19	20	21	22	23	24	25	26	27	28	29	30	31	32	33	34	35	36	37	38	39	40	41	42	43	44
C.acrinaus	Thun	26																1		2	4	4	4	**5**	1	2	2	1				
C.gutturosus	Constance	9		1	**2**	**2**	**2**	1	1																							
C.arenicolus	Constance	3								**1**			**1**						**1**													
C.macrophthalmus	Constance	4																						**2**	1	1						
C.wartmanni	Constance	1																				**1**										

**Species**	**Lake**	**Ntotal**	**PelvF # branched**				**Ntotal**	**PecF # branched**					**Ntotal**	**DF # branched**				**Ntotal**	**AF # branched**			
			**9**	**10**	**11**	**12**		**12**	**13**	**14**	**15**	**16**		**9**	**10**	**11**	**12**		**10**	**11**	**12**	**13**
C.acrinaus	Thun	26	1	**12**	**12**	1	26		1	5	**11**	9	26	1	**15**	8	2	26		**12**	11	3
C.gutturosus	Constance	9	1	3	**5**		9	2	**4**	3			9	1	**8**			9	2	**5**	2	
C.arenicolus	Constance	4			**4**		4	**2**	1	1			4	1	**3**			4	**3**	1		
C.macrophthalmus	Constance	7		**4**	3		7			3	**4**		7	**4**	3			7	1	**3**	1	2
C.wartmanni	Constance	1				**1**	1					**1**	1		**1**			1				**1**

Table S8. PC-loadings of all characters (values > 0.1 or < -0.1 are highlighted in bold), eigenvalues, proportion of explained variance, and R-squared from a linear regression of shape vs. isosize of the retained first three PC-axes from a PCA with all contemporary specimens of the formerly described species *C. alpinus*, *C. albellus,* and *C. fatioi*, their types, the newly described species *C. steinmanni* and *C. profundus* from Lake Thun and the museum specimens with the river Aare as type-locality. See Table 1 and Figure S1 and S2 for descriptions and illustrations of each morphological character.

	**shape PC1**	**shape PC2**	**shape PC3**
**Eigenvalue**	0.117	0.031	0.015
**Variance**	50.42%	13.55%	6.52%
**R-squared**	<0.1	0.33	<0.1
		**PCA-loadings**	
**Character**			
**PELVFB**	**-0.14**	**0.23**	-0.02
**PELVF**	-0.08	-0.07	**-0.16**
**PECFB**	**-0.14**	-0.04	-0.02
**DFB**	**-0.13**	**0.18**	0.01
**DFPe**	0.02	**-0.17**	**-0.40**
**AFB**	-0.07	0.01	-0.05
**AdFB**	**0.21**	**-0.52**	**0.61**
**CD**	**-0.13**	**0.30**	-0.01
**CL**	0.07	**0.25**	**0.32**
**PAdC**	0.03	0.01	**0.34**
**DHL**	-0.08	**-0.18**	-0.07
**PreP**	-0.08	0.05	0.00
**PreA**	-0.06	**0.12**	0.05
**SL**	-0.04	**0.12**	0.10
**PreD**	-0.09	0.10	0.04
**BD**	-0.06	**0.34**	0.06
**PostD**	0.02	**0.11**	**0.22**
**SN**	-0.01	-0.09	-0.09
**ED**	-0.04	**-0.31**	**-0.18**
**EC**	**-0.12**	**-0.28**	**-0.15**
**PostO**	-0.05	0.02	-0.04
**HL**	-0.06	-0.06	-0.07
**HD**	-0.06	-0.06	-0.05
**LJ**	0.01	**-0.17**	-0.10
**HW**	-0.10	-0.06	-0.02
**IOW**	-0.06	-0.05	0.03
**MGR**	**0.63**	**0.13**	**-0.24**
**LGR**	**0.63**	**0.10**	-0.10

Figure S1. Illustration of the morphological body character measurements (see Table 1 in materials and methods for a detailed description of each character).

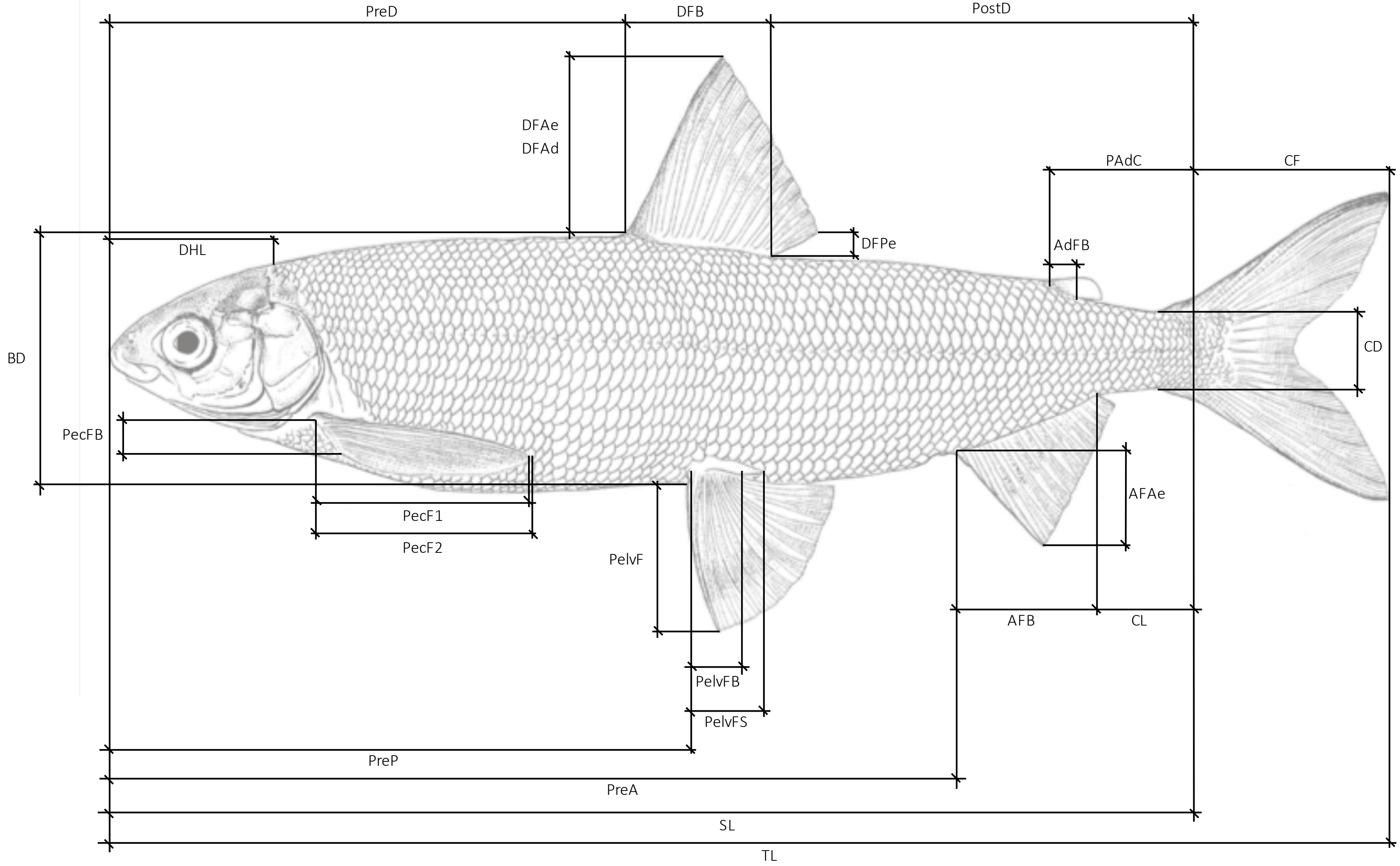

Figure S2. Illustration of the morphological head character measurements (see Table 1 in materials and methods for a detailed description of each character).

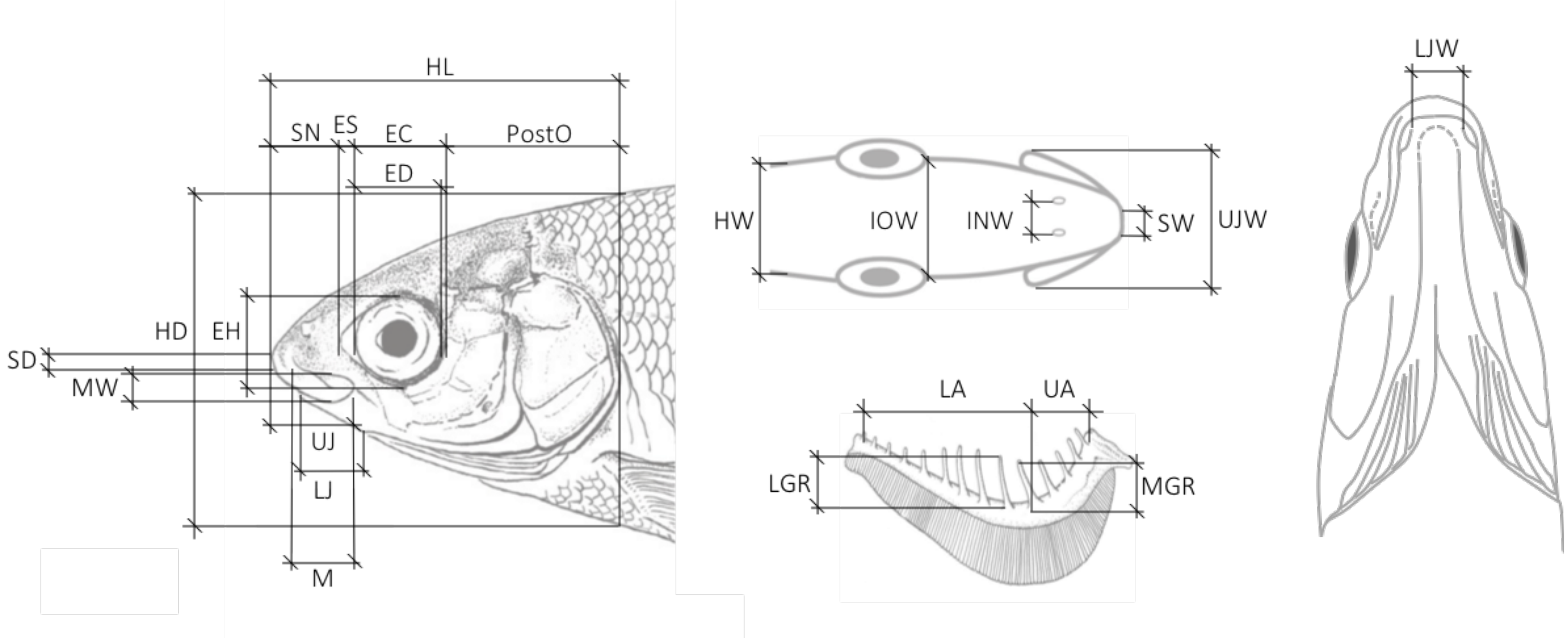

Figure S3. Spawning distribution of the different whitefish species at one spawning site in Lake Thun (Faulensee: 46.673725 / 7.707944) and Lake Brienz (Iseltwald: 46.712079 / 7.961261) during the complete spawning season of the whitefish species from late summer to late winter. The lake surface is indicated in the figures as a horizontal blue line. The distribution of the nets that were set at different depths is indicated along the *y*-axis with grey horizontal lines. For comparison the colours in the figure correspond to those used in the depth distributions figures of Dönz et al. (2018). The depth distribution is shown for mature and ripe fish with genetic assignment probabilities higher than 70% to one of the six species from Dönz et al. (2018) (see Materials and methods section for details on the assignment procedure). We sampled the two spawning sites four times during the whole spawning season along a depth-gradient; once in late September/early October (Thun: 23-24.09.2015; Brienz: 7-8. & 16.10.2015), mid-November (Thun: 16-17.11.2015; Brienz: 23-24.11.2015), late December (Thun: 16-17.12.2015; Brienz: 21-22.12.2015), and late February/early March (Thun: 28.02-1.02.2016; Brienz: 14-15.03.2016). We did not catch any ripe whitefish in March in Lake Brienz, although the spawning season of one of the species (*C. albellus*) is known to have two peaks, once in late summer and one in late winter (Bittner et al. unpublished; Dönz et al. 2018). Thus, the spawning depth distribution in late winter is only shown for Lake Thun. Three multi-mesh benthic nets (Net1 = 15 + 20 mm, Net2 = 25 + 30 + 40 mm, Net3 = 50 + 60 mm) were set at one spawning site along a depth transect from very shallow waters to the lake bottom (2, 10, 30, 60, 100, 150, 200 m and in Lake Brienz at 250 m). To minimize by-catch of other fish species some depths were not fished (2, 10, 30 m) at certain times of the year (September/October and March), since previous work suggests that no spawning whitefish can be found at shallow depths during these time periods (Steinmann 1950; Bittner et al. unpublished; Dönz et al. 2018).

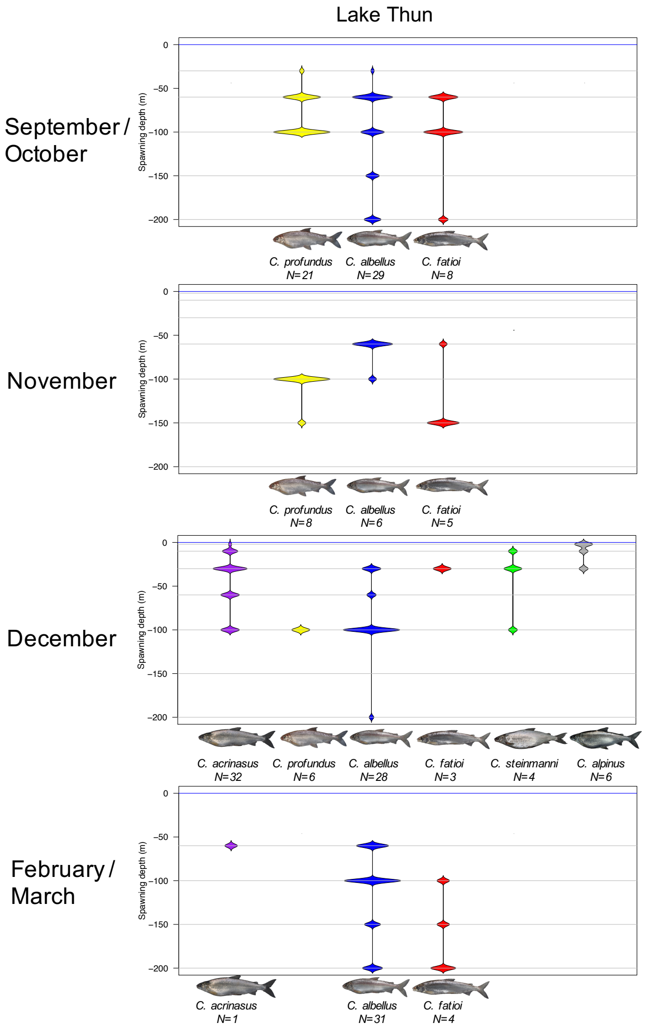

Lake Thun
Lake Brienz
September / October
November
December
February / March
Spawning depth (m)
0
−50
−100
−150
−200
−250
C. profundus N=21
C. albellus N=29
C. fatioi N=8
C. albellus N=6
C. profundus N=8
C. albellus N=6
C. fatioi N=5
C. fatioi N=5
C. acrinasus N=32
C. profundus N=6
C. albellus N=28
C. fatioi N=3
C. steinmanni N=4
C. alpinus N=6
C. albellus N=1
C. fatioi N=4
C. brienzii N=3
C. alpinus N=1
C. acrinasus N=1
C. albellus N=31
C. fatioi N=4

Figure S4. Size (TL=total length in mm) at age of the species *C. alpinus* (A), *C. albellus* (B) *C. steinmanni* (C), *C. fatioi* (D), *C. acrinasus* (E), and *C. profundus* (F) from Lake Thun.

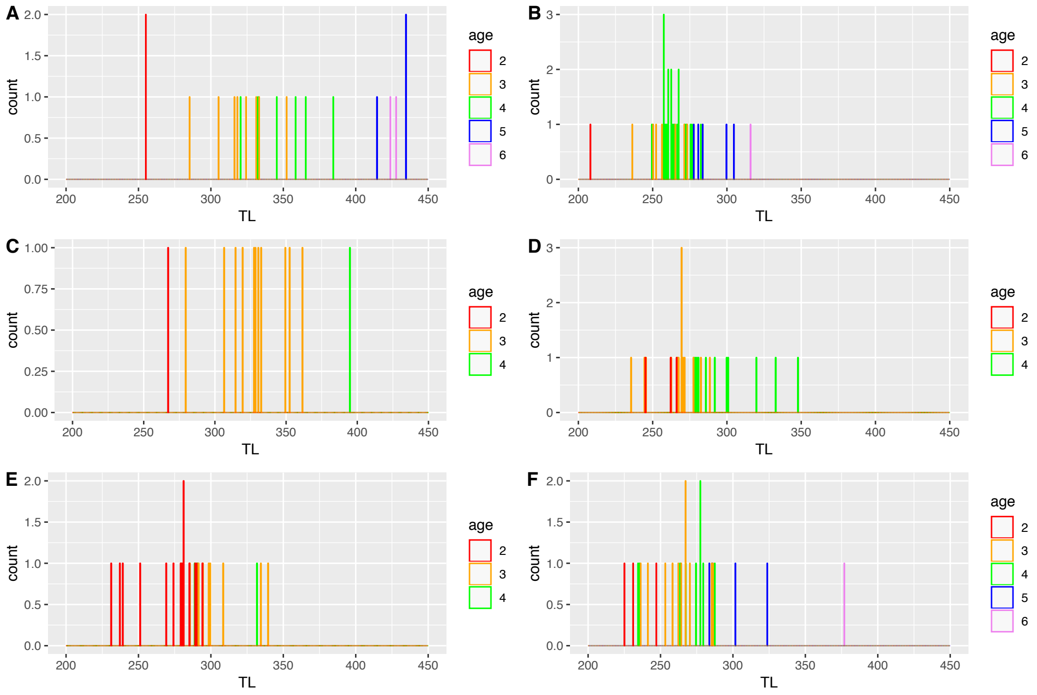

Figure S5. Size (TL = total length in mm) at age of the species *C. alpinus* (A), *C. albellus* (B), *C. brienzii* (C), and *C. fatioi* (D) from Lake Brienz.

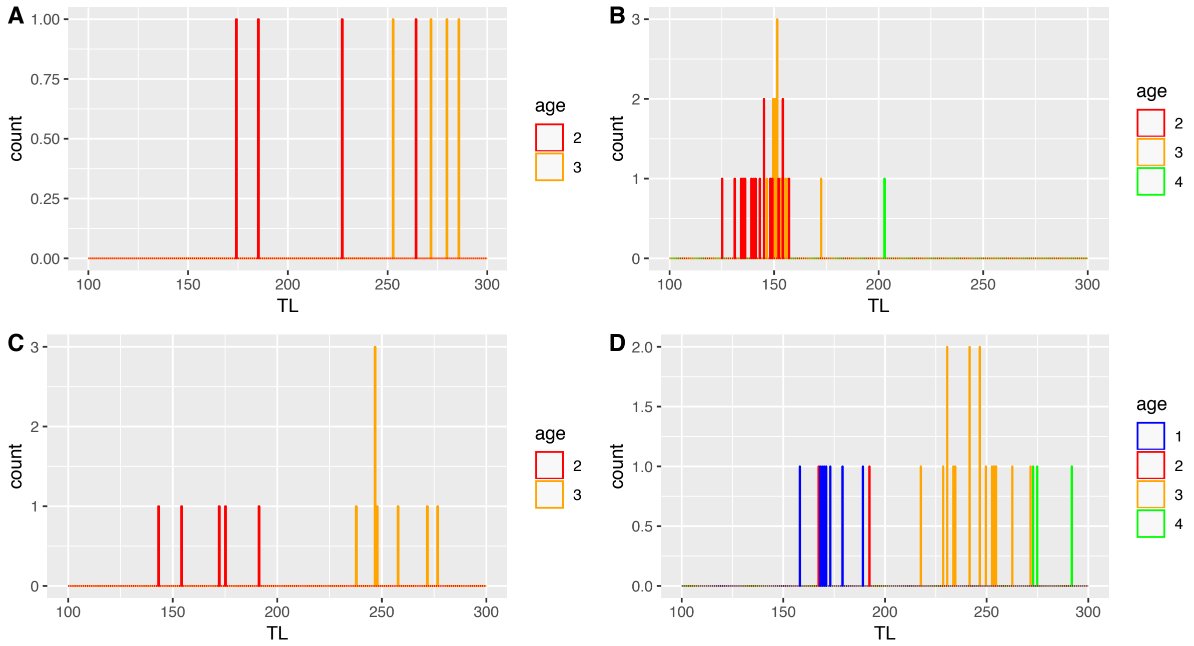

Figure S6. Size (TL = total length in mm) of 3-year-old fish of the species *C. acrinasus* (red), *C. alpinus* (blue), *C. steinmanni* (light blue), *C. albellus* (green), *C. profundus* (orange), *C. fatioi* (violet) and *C. brienzii* (black) from lakes Thun (A) and Brienz (B).

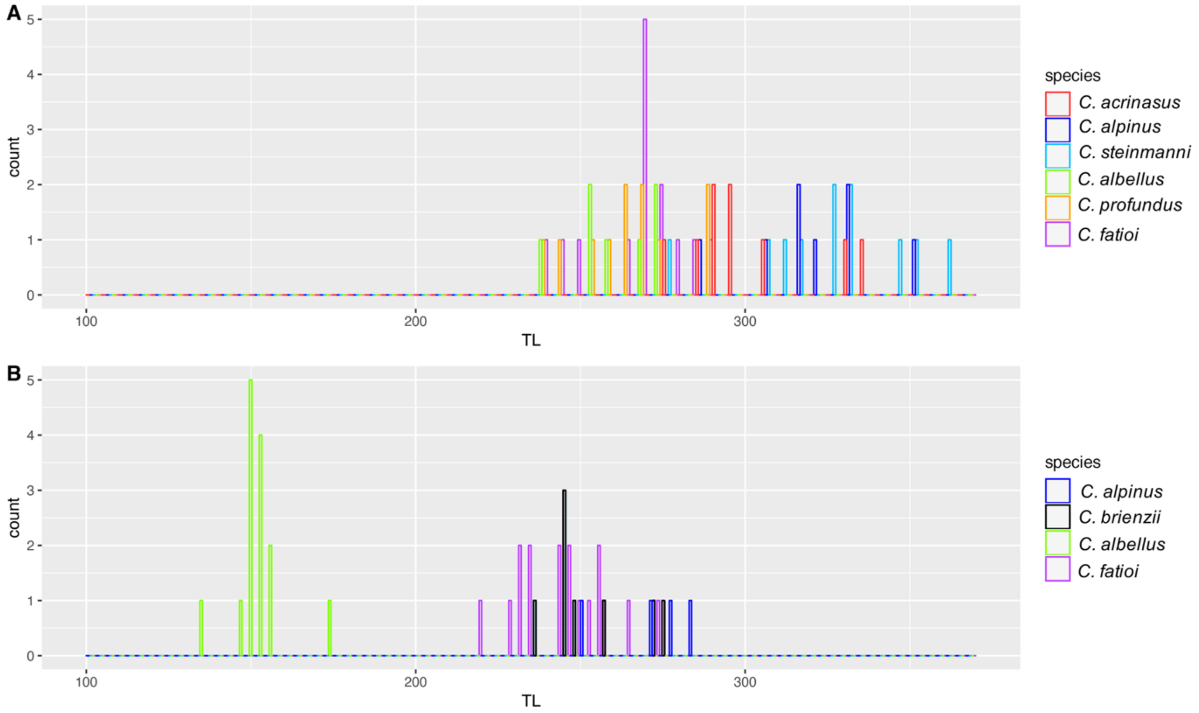

Figure S7. The "cheetah look" of some specimens of *C. albellus* from Lake Brienz with rather large pigmented dots arranged more or less in rows on the upper dorsum.

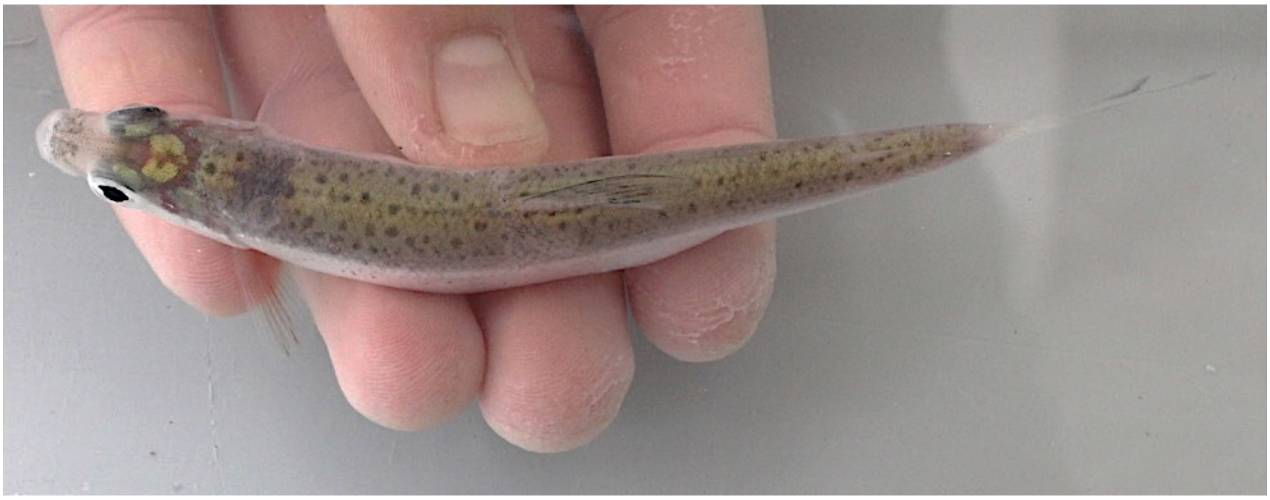

Figure S8. The three main colouration types found on the dorsum and the dorsal part of the flanks above the lateral line in the whitefish species of Lakes Thun and Brienz. Colouration of the species *C. alpinus*, *C. steinmanni*, *C. brienzii*, *C. fatioi,* and *C. acrinasus* have some variation of mostly greenish blue as depicted for a specimen of *C. acrinasus* (left). Some specimens of *C. fatioi* from Lake Thun sometimes show a colouration that resembles that found in *C. albellus* from Lake Thun, which ranges from a pale rose to pale brown as depicted for a specimen of *C. albellus* from Thun (right). Also, some individuals of *C. albellus* from both lakes show a light greenish colouration similar to that of *C. fatioi*. *Coregonus profundus* has a brown-orange colouration and the pectoral fins often have a yellowish colouration as depicted for a specimen of this species (middle). However, the yellowish colouration of the pectoral fins is not unique to *C. profundus* as it can sometimes also be found among specimens of the other species. For details on the colouration of each species see the description in the main text.

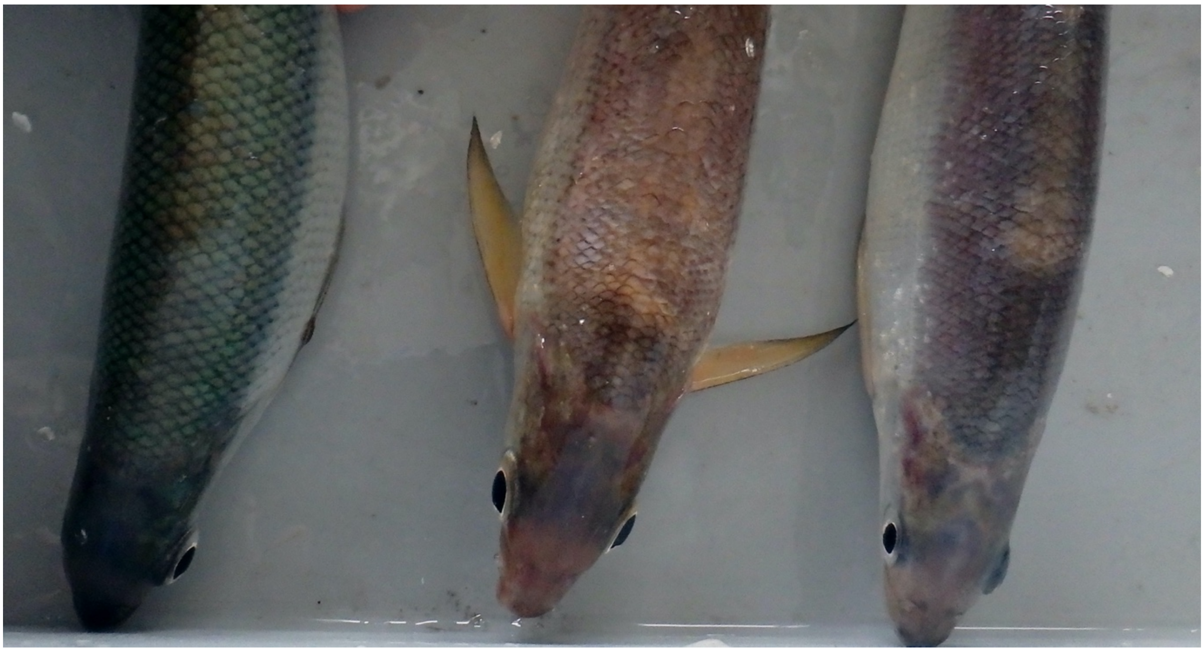

Figure S9. Scatterplot of the first three shape PC axes and the total number of gill rakers for the contemporary whitefish species from Lake Thun and the three contemporary specimens of whitefish from Lake Biel that were caught on the 9th of October 2016 and were genetically assigned (see Materials and methods for details on the genetic assignments) to *C. albellus* (specimens NMBE-1077160 with 84% and NMBE-1077159 with 94% genetic assignment) or *C. profundus* (specimen NMBE-1077158 with 96% genetic assignment). (**A, B**) Shape PCA of the first vs. the second or third PC-axes including a total of 48 measured morphological characters (Table 1). **(C)** The first PC-axes is plotted against the total number of gill rakers. The symbols of the three specimens of whitefish from Lake Biel are enlarged. The proportion of variance explained by each shape PC is given in brackets and the PC-loadings and amount of shape variation explained by size in Table S5. The first three PC-axes explain together 56.15% of the variation in shape.

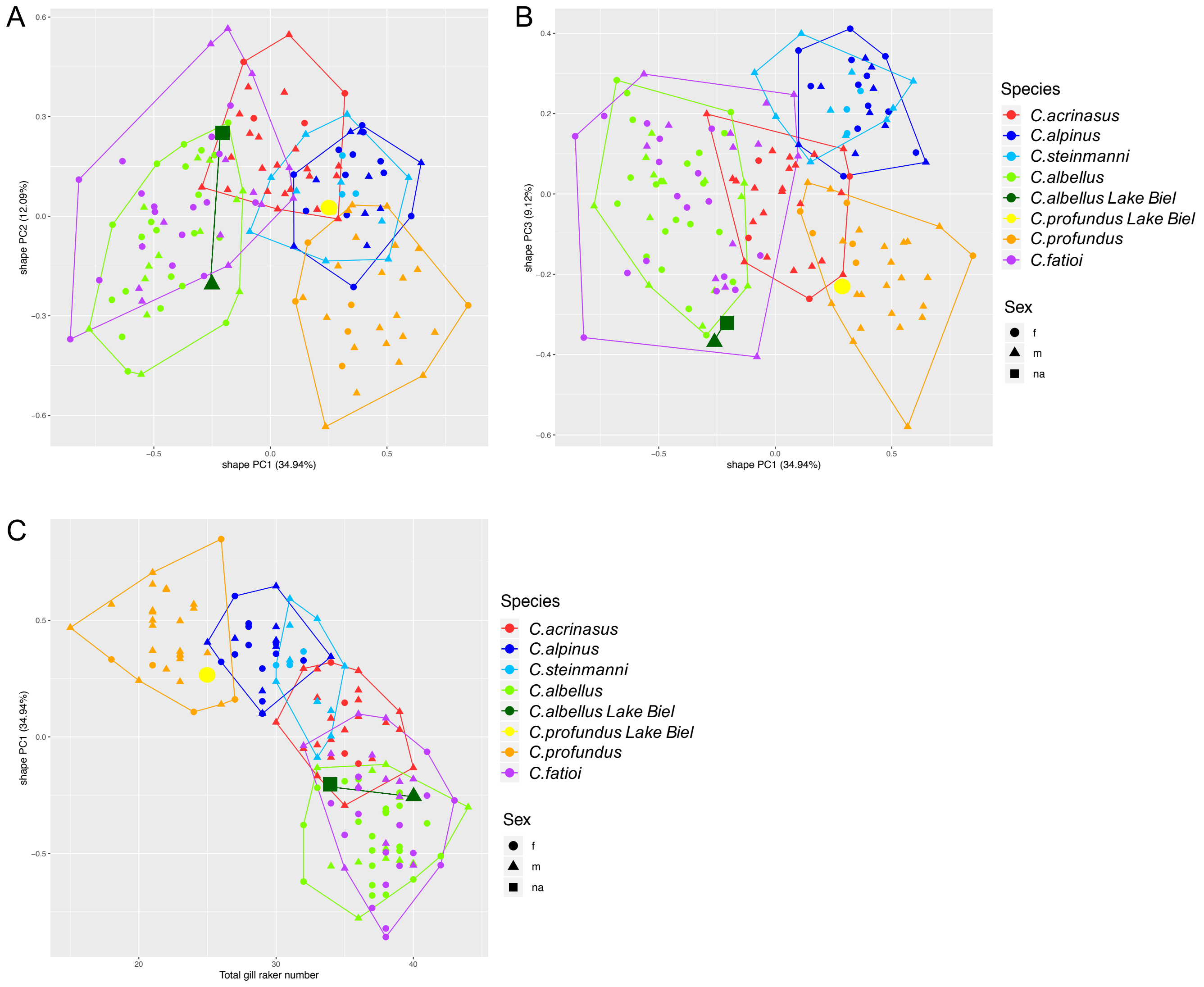

Figure S10. Map of Lake Thun, Brienz, and Biel and the locations where all contemporary specimens of the six species of Lake Thun (*C. alpinus*, *C. steinmanni*, *C. fatioi*, *C. albellus*, *C. acrinasus,* and *C. profundus*) and the four species of Lake Brienz (*C. alpinus*, *C. brienzii*, *C. fatioi*, *C. albellus*) were caught as well as the locations of the contemporary specimens of the 2 whitefish species from Lakes Thun and Brienz that were caught in Lake Biel. The specimens come from three different sampling methods: 1. targeted fishing on known spawning grounds of the different species at the respective spawning season and water depth (single dots), 2. targeted fishing each at one spawning site in lake Thun and Brienz four times during the whole spawning season of all species along a depth-gradient (dots within square) and 3. habitat-stratified fishing of the whole lake during the summer months (single dots). The names of the two native whitefish species of Lake Biel, *C. confusus,* Fatio 1885 and *C. palaea,* Cuvier 1829, are highlighted in Lake Biel.

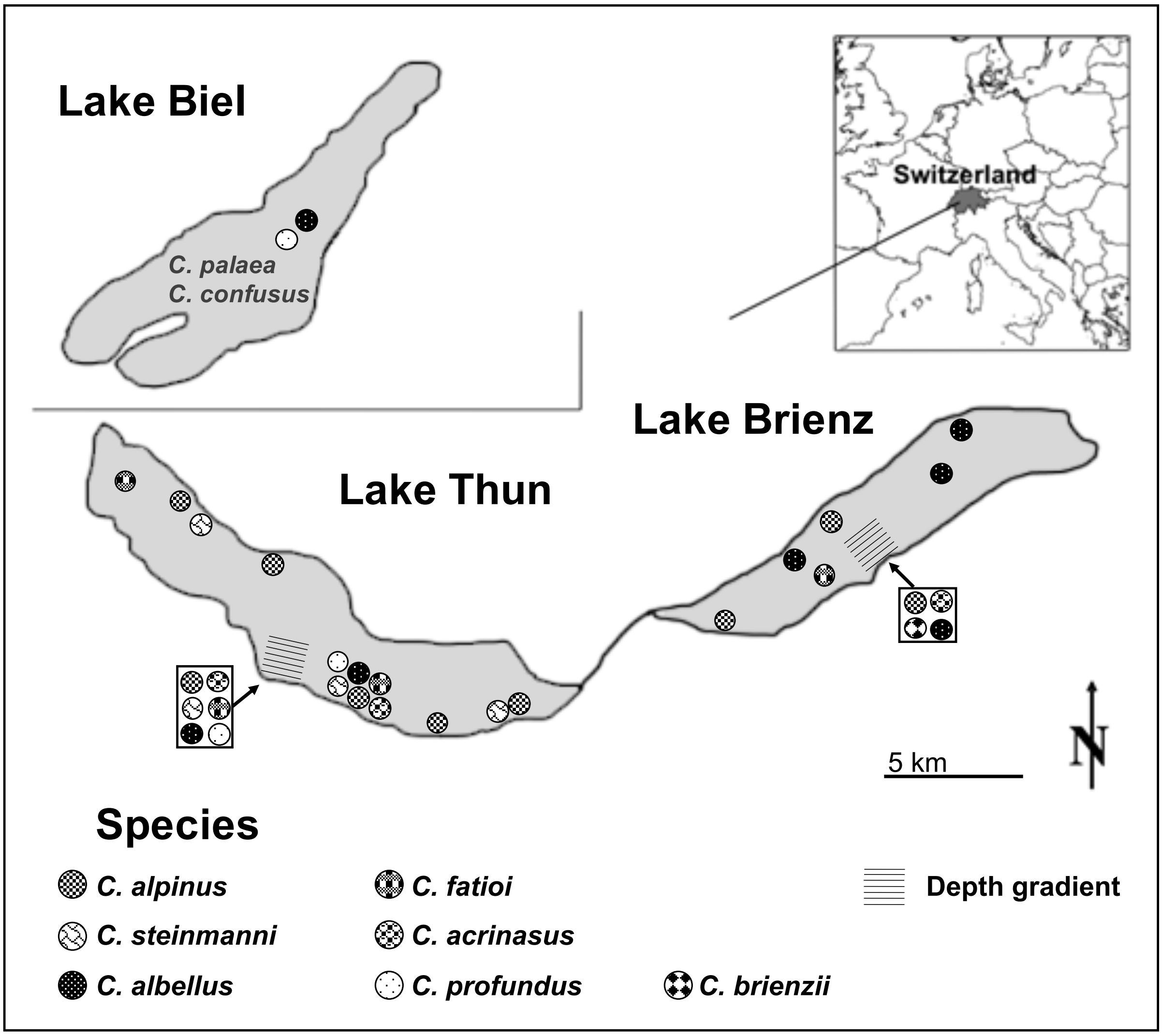

Figure S11. Principal Component Analysis showing that the whitefish specimens caught in the river Aare at the end of the 19th century and mid-twentieth century (locality: either near the city of Bern (specimens: NMBE-1013589 and NMBE-1013603), below the outflow of Lake Thun near the city of Thun (specimens: Eawag-363-1 and Eawag-363-2) or in the Bödeli-Aare between the lakes Thun and Brienz near the city of Interlaken (specimens: Eawag-373-1 and Eawag-373-2) lie within the range or adjacent to the range of the contemporary specimens of *C. fatioi.* (**A, B**) Shape PCA of the first vs. the second or third PC-axes explain together 70.57% of the variation in shape and are based on a subset (Suppl. material 1: Table S8) of 28 out of a total of 48 measured linear morphological characters (Table 1; the remaining 20 characters could not be measured on some specimens. The proportion of variance explained by each shape PC is given in brackets in the axis legend. PC-loadings and amount of shape variation explained by size are reported in Suppl. material 1: Table S8. (**C**) Photos of the fish with the year of capture, size (standard length = SL) and gill raker (GR) information. White scale bars are 1cm.

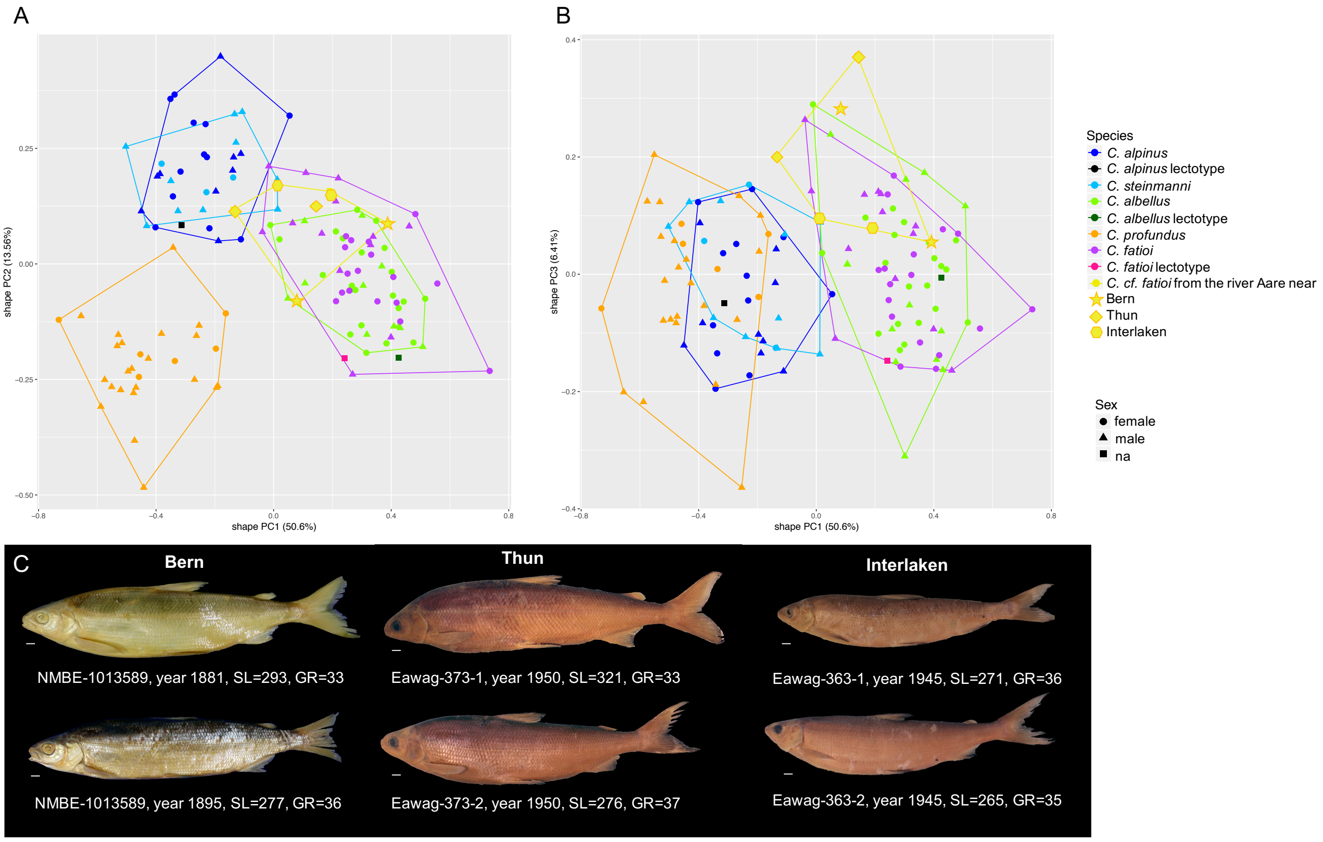

Supplement: Supplementary material 1 — Tables S1-S8, Figures S1-S11 [file zookeys-989-079-s001.pdf]
